# Metabolic feedbacks drive population dynamics and can lead to oscillations among leaf bacteria

**DOI:** 10.1038/s41467-026-73686-w

**Published:** 2026-05-29

**Authors:** Alan R. Pacheco, Giovanni Stefano Ugolini, Simon H. Rüdisser, Andrea Zamuner, Miriam Bortfeld-Miller, Patrick Kiefer, Franziska Oschmann, Samuel G. V. Charlton, Michael Berger, Tommaso Redaelli, Miguel Ángel Salazar, Ilija Dukovski, Jan Roelof van der Meer, Olga T. Schubert, Martin Ackermann, Roman Stocker, Julia A. Vorholt

**Affiliations:** 1 Institute of Microbiology, Department of Biology, ETH Zurich, Zurich, Switzerland; 2 Institute of Environmental Engineering, Department of Civil, Environmental and Geomatic Engineering, ETH Zurich, Zurich, Switzerland; 3 Biomolecular NMR Spectroscopy Platform, Department of Biology, ETH Zurich, Zurich, Switzerland; 4 Scientific IT Services, ETH Zurich, Zurich, Switzerland; 5 Bioinformatics Program, Faculty of Computing and Data Sciences, Boston University, Boston, MA USA; 6 Biological Design Center, Boston University, Boston, MA USA; 7 Center for Advanced Interdisciplinary Research, Ss. Cyril and Methodius University, Skopje, Macedonia; 8 Department of Fundamental Microbiology, University of Lausanne, Lausanne, Switzerland; 9 Department of Environmental Systems Science, ETH Zurich, Zurich, Switzerland; 10 Department of Environmental Microbiology, Swiss Federal Institute of Aquatic Science and Technology (Eawag), Dübendorf, Switzerland; 11 School of Architecture, Civil and Environmental Engineering, Swiss Federal Institute of Technology Lausanne (EPFL), Lausanne, Switzerland

**Keywords:** Microbiome, Bacteria

## Abstract

Metabolic interactions are fundamental to the assembly and function of microbiomes. Yet, our understanding of how specific interaction mechanisms can drive broader ecological outcomes and population dynamics remains limited. Here, we monitor interactions resulting from plant oligosaccharide degradation by leaf-associated bacteria using a microfluidic device that enables direct cell observation and quantitative metabolite detection. This approach enables the identification of key metabolic mediators, revealing recipient-specific patterns of carbon substrate and cofactor complementation. By linking these patterns to emergent dynamics observed between pairs of bacteria, we identify metabolically driven feedbacks that could lead to a variety of ecological outcomes – from outcompetition to coexistence characterized by oscillating population abundances. Investigating these observations with metabolic modeling allows us to systematically assess the impact of specific molecular mediators on population dynamics, yielding predictions of interaction outcomes that we validate experimentally. Our results provide a detailed mapping of metabolic mechanisms to emergent population trajectories among environmental microbes and help inform strategies for designing microbiomes with desired steady states.

## Abstract (web summary)

Plant-associated bacteria engage in complex metabolic interactions. Here authors used microfluidics and computational modeling to identify molecular drivers of these interactions and disentangle their contributions to emergent ecological dynamics.

## Introduction

Metabolic interactions are crucial for shaping the assembly of microbial ecosystems^1,2^. By taking up resources and secreting metabolic byproducts and cellular building blocks, microbes continuously alter their immediate environments and can promote or inhibit the growth of other species. Though advances in experimental systems, high-throughput sequencing, and bioinformatics have produced detailed knowledge on the structure and function of host-associated and environmental microbiomes, it has remained challenging to resolve the mechanisms that underly these interspecies metabolic interactions. The processes that drive these interactions encompass not only the specific organisms and chemical species involved, but also the physiological changes that the organisms experience and any spatiotemporal dynamics that emerge. An ability to link metabolic mediators to changes in community composition would produce a more comprehensive understanding of how complex microbiota assemble. Moreover, as the composition of communities impacts their function, detailed knowledge of metabolic interactions would help advance efforts to engineer microbiota with desired steady states and effects on their host ecosystems.

The phyllosphere, defined as the above-ground parts of plants, is one of the largest surface areas for microbial colonization on the planet^3^. Though it is a largely oligotrophic environment prone to fluctuating resource, moisture, and light conditions, it hosts a rich array of commensal microbial species of which the majority are bacteria^4,5^. These phyllosphere bacteria, in addition to conferring beneficial effects on their hosts such as protection against pathogens and stress tolerance, exhibit a diverse repertoire of metabolic capabilities that forms the basis for complex interspecies interaction networks^6–8^. Though metabolic competition and cross-feeding have been suggested to shape interaction outcomes for phyllosphere bacteria in situ^9^, the physiological causes and effects of these phenomena—as well as their contributions to community function—have only begun to be resolved on an organism-by-organism basis^10^.

Synthetic communities composed of representative microbes from natural environments provide an avenue to systematically explore the factors underlying these interaction outcomes^11,12^. They can reveal causal mechanisms of interactions between organisms and have recently shed light on the effects of carbon flux and cross-feeding among host-associated and environmental microbiota members^13,14^. Despite a growing understanding of trophic exchanges as drivers of ecological patterns, there are additional features of microbial metabolism that must be considered to gain a more complete understanding of their roles in a community setting. In particular, the inability of many organisms to produce essential metabolites—such as vitamins or amino acids—and the potential for others to complement these biosynthetic deficiencies^15,16^, add further components to metabolic interactions that can have profound effects on the structure of a community. As such, the ways in which these individual factors can come together to promote the growth of certain organisms, as well as the spatiotemporal dynamics and steady states that can emerge as a result, depend critically on the substrates available in a given local environment at the microscale.

In the phyllosphere, such substrates include simple sugars such as glucose, fructose, and sucrose that can diffuse or leach through the cuticle, where they serve as primary carbon sources for epiphytic bacteria^17–19^. Xylan, a key component of plant hemicellulose and one of the most abundant biopolymers on Earth^20,21^, may also serve as a carbon source following enzymatic breakdown. Recent work revealed that bacterial competition on healthy *Arabidopsis thaliana* leaves can induce cell wall-degrading enzyme expression: the commensal *Sphingomonas* Leaf257 upregulates multiple xylanases and xylosidases during co-colonization with *Rhizobium* Leaf68, enabling utilization of xylan-derived substrates as an apparent niche-partitioning strategy^22^. In addition, *Xanthomonas* strains from the *Arabidopsis* leaf microbiota constitutively secrete cell wall-degrading enzymes, including xylanases, via their type-2 secretion system^23^. The degradation of xylan by primary consumers could thus generate oligosaccharide intermediates or sugar monomers that support secondary consumers, potentially establishing trophic dependencies among phyllosphere community members. Combined with the widespread vitamin auxotrophies observed in leaf-associated bacteria^15^, such substrate cross-feeding may give rise to multi-layered metabolic interactions.

Here, we explore the mechanisms and dynamics of metabolic interactions emerging from the degradation of xylan among a representative set of leaf-associated bacteria from *A. thaliana*^24^. Our experimental approach is centered on the use of a microfluidic device for controlled loading and direct observation of pairs of species. This approach enables us to monitor organism-specific growth and spatiotemporal population dynamics while simultaneously disentangling the metabolic components underlying the interactions. By coupling this approach to computational models of organism-specific metabolic properties, we investigate how the interplay between carbon sources, vitamin sharing, and growth rates can produce divergent community dynamics, enabling validated predictions of ecological steady states.

## Results

### Metabolic dependencies emerge across phyllosphere microbiota members during growth on xylan

To investigate how xylan degradation may shape metabolic interactions between phyllosphere bacteria, we first selected two members of the Alphaproteobacteria, the most abundant class found on *A. thaliana* leaves^5^. These strains, *Sphingomonas* Leaf257 and *Rhizobium* Leaf68, have been shown to naturally co-localize on leaf surfaces^22^ and interact in a manner that involves upregulation of xylan degradation genes by *Sphingomonas* Leaf257^22^. Despite this role of xylan degradation in shaping interactions in situ, its contribution to bacterial growth dynamics at the microscale has only begun to be explored^23,25^. As genomic analysis indicates that strains from a number of leaf-associated bacterial genera possess genes for cleaving glycosidic linkages in plant cell wall components, including xylan (Supplementary Fig. 1), there may indeed be a broader ecological role for this metabolic mechanism.

**Fig. 1:**
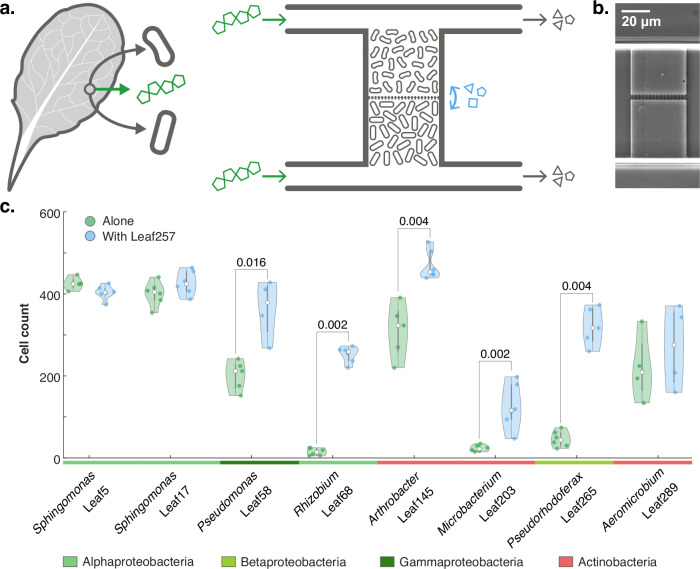
A coupled microfluidic device for monitoring the emergence and development of metabolic interactions. **a** General schematic of the microfluidic device and incorporation of plant-derived xylan and phyllosphere bacteria. Two distinct populations of cells are loaded into the microchambers via parallel channels. Here, two coupled microchambers are shown, which allows the two populations to remain physically separated while exchanging metabolites (blue shapes, center). Constant perfusion of a minimal medium containing xylan (green oligomers, left) is applied, after which metabolites produced by the bacterial populations can be collected for analysis (gray shapes, right). **b** Image of two representative coupled microchambers of a microfluidic device. **c** Identification of interactions driven by *Sphingomonas* Leaf257 in minimal medium containing xylan as the sole carbon source. Cell counts are reported for interactor strains at the end of the observation period (≥20 h, Supplementary Fig. 3), and *p*-values of one-tailed Mann-Whitney $U$ tests are displayed for significant (<0.05) comparisons. Data from *N* ≥ 4 microchambers are shown for each condition (Supplementary Fig. 3). Violin plots display kernel density estimates of cell count distributions with overlays of each data point^64,69^. Boxplots within each violin plot display the median (white circle), interquartile range (gray box), and upper/lower adjacent values (gray whiskers). Source data are provided as a Source Data file.

We hypothesized that degradation of plant-derived xylan alone by *Sphingomonas* Leaf257 could form the basis for a metabolic interaction with *Rhizobium* Leaf68 in vitro. To test this, we loaded the two strains into a microfluidic device that enables real-time observation of two microbial populations separated spatially by an interface^26^ (“Methods”, Fig. 1a, b, Supplementary Fig. 2). This interface can either be continuous (thereby blocking any metabolic exchange between the populations) or consist of a series of nanochannels that enable diffusion of metabolites between them. The strains were cultivated in this device under continuous flow of a minimal medium with 1.32 g/L xylan oligomers (i.e., corresponding to 10 mM of its constituent monomers, “Methods”) as the sole carbon source. When the strains were loaded individually in (pore-less) microchambers that did not allow the populations to interact, we observed the rapid proliferation of *Sphingomonas* Leaf257 and the absence of growth of *Rhizobium* Leaf68 (Supplementary Video 1). When the two populations were allowed to interact, the growth of *Sphingomonas* Leaf257 was followed by the growth of *Rhizobium* Leaf68 (Supplementary Fig. 3, Supplementary Video 2). This growth continued until the chambers containing *Rhizobium* Leaf68 were filled (Fig. 1c, Supplementary Fig. 3), providing evidence of a metabolic dependency for *Rhizobium* Leaf68 in this medium.

We next investigated the degree to which similar dependencies could emerge across a broader set of leaf microbiota members. To do this, we tested the ability of *Sphingomonas* Leaf257 to enable the growth of seven additional leaf bacterial isolates. This set of strains, comprising *Sphingomonas* Leaf5, *Sphingomonas* Leaf17, *Pseudomonas* Leaf58, *Arthrobacter* Leaf145, *Microbacterium* Leaf203, *Pseudorhodoferax* Leaf265, and *Aeromicrobium* Leaf289, represents a taxonomic cross-section of abundant phyllosphere bacteria from *A. thaliana* leaves^24,27,28^ (“Methods”). To determine whether the metabolic activity of *Sphingomonas* Leaf257 could support the growth of these strains, we loaded each into microfluidic devices and again flowed a minimal medium containing xylan. As with *Rhizobium* Leaf68, the growth of each strain was tracked in both microchambers coupled to or uncoupled from those containing *Sphingomonas* Leaf257.

We observed *Microbacterium* Leaf203 and *Pseudorhodoferax* Leaf265 to grow in the presence of xylan only when allowed to interact with *Sphingomonas* Leaf257 (Fig. 1c, Supplementary Fig. 3 and Supplementary Videos 3–6). The remaining strains could independently utilize xylan without *Sphingomonas* Leaf257 (Supplementary Fig. 3), indicating a broader potential for positive metabolic interactions.

Beyond allowing us to identify metabolic dependencies in xylan, our screen also shed light on how the growth of interactor strains could vary with the strength of their coupling to *Sphingomonas* Leaf257. Having observed that the growth of the three strains that strictly depended on *Sphingomonas* Leaf257 (i.e., *Rhizobium* Leaf68, *Microbacterium* Leaf203, and *Pseudorhodoferax* Leaf265) initiated in the region of the microchambers closest to the interface (Supplementary Video 2, Supplementary Video 4 and Supplementary Video 6), we monitored the growth of a representative strain—*Pseudorhodoferax* Leaf265—in microchambers connected to *Sphingomonas* Leaf257 by only 1 to 6 pores (Supplementary Fig. 4). Here, *Pseudorhodoferax* Leaf265 exhibited a radial growth pattern centered on the openings to the growing *Sphingomonas* Leaf257. This area of growth increased with the number of pores (Supplementary Fig. 4), underscoring the dependence of recipient growth on resources from *Sphingomonas* Leaf257.

### Complex metabolic profiles sustain interacting organisms

Because *Sphingomonas* Leaf257 can support the growth of strains from distinct leaf-associated genera, we asked whether the underlying molecular mechanisms could differ, given the varied resource use capabilities of phyllosphere bacteria^9^. We therefore used nuclear magnetic resonance (NMR) spectroscopy to identify the primary carbon sources involved in these interactions, as well as liquid chromatography-mass spectrometry (LC-MS) to identify any vitamins exchanged (“Methods”).

**Fig. 2:**
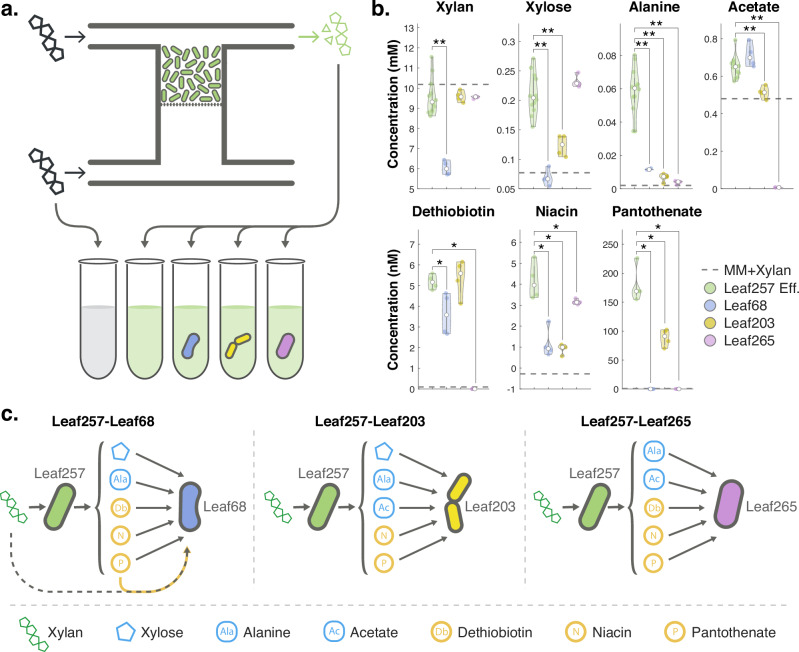
Identification of metabolic components involved in interactions with *Sphingomonas* Leaf257. **a** Experimental schematic. *Sphingomonas* Leaf257 was loaded into chambers on one side of the microfluidic devices, and a constant flow of a minimal medium containing xylan (10 mM of monomeric equivalents) was provided. This minimal medium, as well as the effluent resulting from the growth and metabolic activity of *Sphingomonas* Leaf257, was collected for analysis with NMR and LC-MS. The interactor strains *Rhizobium* Leaf68, *Microbacterium* Leaf203, and *Pseudorhodoferax* Leaf265 were separately cultivated in the harvested effluent. Following their growth (Supplementary Fig. 7), each supernatant was collected for metabolomic analysis. A detailed representation of the device geometry is provided in Supplementary Fig. 2. **b** Statistical comparison of compounds in effluent based on NMR and LC-MS between devices loaded only with MM + xylan (mean of 3 biological replicates for NMR and 4 biological replicates for LC-MS as dashed line, (*) $p < 0.05$, (**) $p < 0.01$, one-tailed Mann-Whitney $U$-tests), devices loaded with *Sphingomonas* Leaf257 in MM + xylan (“Leaf257 Eff.” *N* = 12 measurements for NMR, 4 measurements for LC-MS), and effluents consumed by each interactor strain (*N* = 4 measurements per strain for NMR and LC-MS). NMR spectra comparing compounds in all conditions are shown in Supplementary Figs. 5, 8. Violin plots display kernel density estimates of metabolite abundance distributions with overlays of each data point^64,69^. Boxplots within each violin plot display the median (white circle), interquartile range (gray box), and upper/lower adjacent values (gray whiskers). **c** Strain-specific conceptual model of metabolic complementation for *Arabidopsis* leaf-associated bacteria based on xylan degradation (structures of xylan and xylose are simplified). Source data are provided as a Source Data file.

We quantified changes in metabolite abundances stemming from the growth of *Sphingomonas* Leaf257. We loaded this strain into one side of the microfluidic devices, applied a constant flow of a minimal medium containing xylan, and continuously collected the resulting effluent (Fig. 2a). NMR measurements of the effluent revealed several changes in metabolite abundances (Fig. 2b, Supplementary Fig. 5 and Supplementary Data 1). We observed a reduction in xylan and the appearance of xylose in the effluent, confirming the ability of *Sphingomonas* Leaf257 to degrade xylan into its constituent monomers. Due to the possible underestimation of xylan degradation in the effluent stemming from the continuous replenishment inherent to the microfluidic device, we carried out a separate experiment in a batch culture system that further confirmed this degradation (Supplementary Fig. 6). Furthermore, we identified an increased abundance of acetate in the microfluidic effluent, possibly due to deacetylation of the xylan oligomer and/or secretion by *Sphingomonas* Leaf257 (Fig. 2b). Our measurements also indicated the consistent presence of alanine in the microfluidic effluent, suggesting its leakage or exudation by *Sphingomonas* Leaf257, contributing to a diverse profile of carbon conversion by this strain.

This carbon secretion profile prompted us to ask whether these compounds played a role in the metabolic dependencies observed in the microfluidic devices. We therefore incubated *Rhizobium* Leaf68, *Microbacterium* Leaf203, and *Pseudorhodoferax* Leaf265 separately on the effluents gathered from *Sphingomonas* Leaf257 (Fig. 2a) and sampled their culture supernatants following growth. Though all three strains exhibited growth as quantified by cell density (Supplementary Fig. 7), their metabolite uptake profiles differed (Fig. 2b, Supplementary Fig. 8). Specifically, we observed *Rhizobium* Leaf68 and *Microbacterium* Leaf203, but not *Pseudorhodoferax* Leaf265, to take up xylose. This contrasted with our expectation that xylose exchange would be the main driver of growth in all the recipient strains, given the breakdown of the xylan oligomer. The inability of *Pseudorhodoferax* Leaf265 to utilize xylose as a sole carbon source^9^ pointed to possible roles of acetate and alanine in its facilitation. Indeed, only *Pseudorhodoferax* Leaf265 took up all the acetate present in the effluent, while all three strains exhausted the alanine supply.

Our NMR measurements also indicated that *Rhizobium* Leaf68 could degrade xylan, which it was unable to utilize in the minimal medium, pointing to additional modes of facilitation by *Sphingomonas* Leaf257. We therefore hypothesized that cofactor complementation to *Rhizobium* Leaf68—a known auxotroph for biotin, NAD, and CoA^15,22^ (Supplementary Fig. 9)—could allow it to utilize not only the carbon sources made available by *Sphingomonas* Leaf257, but the oligomer itself. Indeed, LC-MS analysis of the effluent (Fig. 2b) revealed the presence of pantothenate, which was detected at a mean concentration of 180 nM. In addition, niacin and dethiobiotin were detected at mean concentrations of 4 and 5 nM, respectively.

By further analyzing the supernatants of interactor strains cultivated on the effluent of *Sphingomonas* Leaf257, we determined that *Rhizobium* Leaf68 indeed took up dethiobiotin, niacin, and pantothenate to complement its respective auxotrophies (Fig. 2b). Notably, we observed *Rhizobium* Leaf68 to exhaust the supply of pantothenate in the effluent, which suggests this auxotrophy to be limiting^15^ and to potentially underly its incomplete utilization of the carbon sources and remaining two vitamins.

In addition to the vitamins utilized by *Rhizobium* Leaf68, we observed *Microbacterium* Leaf203 and *Pseudorhodoferax* Leaf265 to take up pantothenate and niacin from the *Sphingomonas* Leaf257 effluent, with *Pseudorhodoferax* Leaf265 additionally exhausting the supply of dethiobiotin. As with *Rhizobium* Leaf68, an analysis of the sequenced genomes of *Microbacterium* Leaf203 and *Pseudorhodoferax* Leaf265 revealed the absence of ketopantoate hydroxymethyltransferase (PanB), which, together with separate growth assays in vitamin-deficient media (Supplementary Fig. 9), provided evidence for *Sphingomonas* Leaf257 complementing pantothenate/CoA auxotrophies in *Microbacterium* Leaf203 and *Pseudorhodoferax* Leaf265. Interestingly, though niacin was observed to be taken up by both *Microbacterium* Leaf203 and *Pseudorhodoferax* Leaf265 from the microfluidic-derived effluent, separate growth assays revealed that both strains are NAD prototrophs, suggesting that they can take up niacin to support cofactor biosynthesis without a strict need for exogenous complementation (Supplementary Fig. 9). Lastly, though both *Microbacterium* Leaf203 and *Pseudorhodoferax* Leaf265 lack the same gene that underlies the biotin auxotrophy in *Rhizobium* Leaf68 (dethiobiotin synthase, BioD), the lack of dethiobiotin uptake by *Microbacterium* Leaf203 and the growth of both strains in biotin-deplete medium (Supplementary Fig. 9) challenge a conclusive determination of a biosynthetic deficiency. While a capacity for storing biotin or biotin precursors cannot be ruled out, the exhaustion of *Sphingomonas* Leaf257-derived dethiobiotin by *Pseudorhodoferax* Leaf265 nonetheless supports a beneficial effect of its exchange for this strain. Taken together, this analysis informs recipient-specific metabolic exchange and vitamin complementation from plant-derived xylan (Fig. 2c), which we validated in an additional in vitro experiment (Supplementary Fig. 10).

### Strain-specific interaction dynamics and temporal population fluctuations emerge from xylan degradation

Having determined key metabolic drivers of dependencies between representative leaf bacteria, we sought to explore their contribution to population dynamics between these strains when interacting directly, simulating natural encounters at the microscale^4,29^. We therefore used a fluorescently labeled *Sphingomonas* Leaf257 and co-loaded it with an unlabeled interactor strain into the same side of the microfluidic devices (i.e., into the same microchambers). This setup enabled us to quantify the area within the individual microchambers occupied by each strain over time, providing insights into their interaction dynamics.

**Fig. 3:**
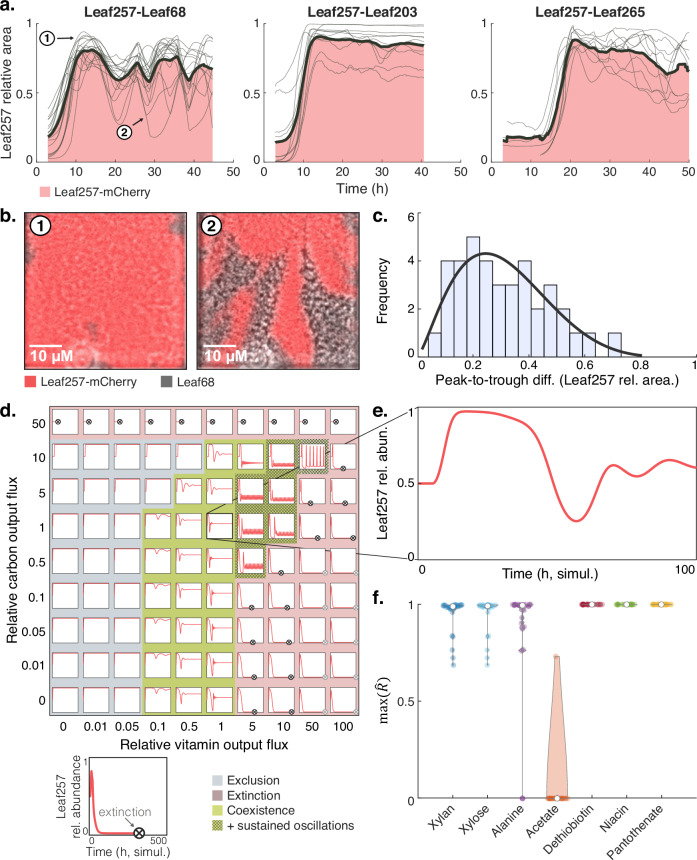
Emergent dynamics for interaction between *Sphingomonas* Leaf257 and interactor strains. **a** Relative microchamber area occupied by fluorescently labeled *Sphingomonas* Leaf257 with xylan as sole carbon source inoculated at equal abundance with *Rhizobium* Leaf68 (left, *N* = 18 microchambers, Supplementary Video 9), *Microbacterium* Leaf203 (middle, *N* = 11 microchambers, Supplementary Video 7), and *Pseudorhodoferax* Leaf265 (right, *N* = 10 microchambers, Supplementary Video 8). Thick lines represent the mean relative area of *Sphingomonas* Leaf257 over time. In all three pairings, growth of each interactor strain begins after the initial filling of the chamber by *Sphingomonas* Leaf257 (i.e., after approximately 10–20 h of growth). **b** Representative individual microchambers containing *Sphingomonas* Leaf257 and *Rhizobium* Leaf68 at time of high (1, left) and low (2, right) relative areas of *Sphingomonas* Leaf257 (Supplementary Video 9). **c** Distribution of peak-to-trough differences in *Sphingomonas* Leaf257 relative area for each fluctuation with *Rhizobium* Leaf68 in panel (**a**). **d** Predicted relative abundance of *Sphingomonas* Leaf257 in metabolic modeling simulations with *Rhizobium* Leaf68 across varied ranges of carbon and vitamin output flux by *Sphingomonas* Leaf257. Rates of carbon and vitamin output are shown as relative to those estimated from NMR and LC-MS data (Fig. 2b, “Methods”). Gray ‘x’ symbols indicate extinction events that occurred outside the displayed time window. **e** Details of representative simulated times course with rates of carbon and vitamin output corresponding to those estimated from NMR and LC-MS data. **f** Distributions of maximum cross-correlation coefficients $\hat{R}$ between *Rhizobium* Leaf68 growth rate and uptake of carbon sources or vitamins in simulations that resulted in coexistence (i.e., 29 simulations denoted with a green background in panel (**d**)). Violin plots display kernel density estimates of maximum cross-correlation coefficient distributions with overlays of each data point^64,69^. Boxplots within each violin plot display the median (white circle), interquartile range (gray box), and upper/lower adjacent values (gray whiskers). Source data are provided as a Source Data file.

We observed the emergence of distinct and reproducible dynamic patterns for each interactor strain population when paired with *Sphingomonas* Leaf257 (Fig. 3a, Supplementary Videos 7–9). Common to all three interactions was the rapid initial growth of *Sphingomonas* Leaf257 as it utilized xylan and expanded within the microchambers. Following this initial growth phase, however, the growth dynamics diverged according to strain. In the case of *Microbacterium* Leaf203, *Sphingomonas* Leaf257 remained dominant, making up on average 84% ( ± 12%, s.d.) of the population by area during approximately 40 h of monitoring. In contrast, *Pseudorhodoferax* Leaf265 and *Rhizobium* Leaf68 made up higher fractions of the population (Fig. 3a), likely due to their higher growth rates when paired with *Sphingomonas* Leaf257 (Supplementary Fig. 3).

The pairing of *Sphingomonas* Leaf257 and *Rhizobium* Leaf68 resulted in consistent fluctuations in the relative areas occupied by each strain (Fig. 3a–c, Supplementary Video 9), an intriguing pattern given the apparent absence of antagonistic interactions other than resource competition between these strains. This pattern, which in some cases saw the *Sphingomonas* Leaf257 population changing by more than half of the chamber area (Fig. 3c), raised the broader question of how such fluctuations—or even sustained stable oscillations—could emerge among environmental strains from metabolic complementation alone.

### Computational simulations reveal carbon and vitamin thresholds for sustained interactions

To determine whether and how metabolic complementation could recapitulate the dynamics observed for *Sphingomonas* Leaf257 and *Rhizobium* Leaf68, we leveraged a computational modeling approach that relies on detailed knowledge of the metabolic capabilities and requirements of each strain. Specifically, we used a set of previously validated genome-scale metabolic models for phyllosphere bacteria that have previously been shown to recapitulate interaction outcomes in situ, and that includes *Sphingomonas* Leaf257 and *Rhizobium* Leaf68^9^. Here, we further refined the models for these two strains according to the additional knowledge of their physiology gained in this study, incorporating their ability to degrade plant-derived xylan, the release of carbon sources and vitamins by *Sphingomonas* Leaf257, as well as the known vitamin auxotrophies for *Rhizobium* Leaf68 (“Methods”). We then used COMETS, a metabolic modeling tool that simulates organism- and resource-specific dynamics^30,31^, to study the establishment and development of the interaction between the two strains.

We first sought to explore the space of interaction dynamics that could emerge between *Sphingomonas* Leaf257 and *Rhizobium* Leaf68 in silico. We estimated the rate of output of carbon (i.e., of xylose, alanine, and acetate) and vitamins (i.e., of pantothenate, niacin, and dethiobiotin) by *Sphingomonas* Leaf257 based on our metabolomic experiments (“Methods”, Fig. 2b). We then simulated the interaction with *Rhizobium* Leaf68 at higher and lower degrees of nutrient exchange by varying the rate of carbon and vitamin output by *Sphingomonas* Leaf257 while keeping the availability of the xylan substrate constant. These simulations revealed three distinct dynamic regimes (Fig. 3d). Low rates of vitamin output by *Sphingomonas* Leaf257 (0-5% of experimental estimates) – led to the dominance of this strain, suggesting a lower bound of vitamin output necessary to sustain the growth of *Rhizobium* Leaf68. In contrast, high rates of carbon and vitamin output by *Sphingomonas* Leaf257 (>10 times experimental estimates) led to population collapse through distinct mechanisms: at high carbon output (50 times the estimated rate), the reallocation of resources to xylose, alanine, and acetate decreased the growth rate of *Sphingomonas* Leaf257, preventing it from overcoming the simulated dilution and providing resources to *Rhizobium* Leaf68. Conversely, high vitamin output resulted in an excess of vitamins, leading to the rapid growth of *Rhizobium* Leaf68 and the outcompetition of *Sphingomonas* Leaf257. This loss of the *Sphingomonas* Leaf257 population was followed by the eventual collapse of the *Rhizobium* Leaf68 population, due to exhaustion of the vitamins necessary for its growth.

Between these outcomes of outcompetition and extinction, our simulations revealed a third dynamic regime that allowed for the coexistence of *Sphingomonas* Leaf257 and *Rhizobium* Leaf68 (Fig. 3d, e). This regime, characterized by rates of carbon and vitamin output at similar orders of magnitude to those estimated from experimental data, comprised interaction dynamics that qualitatively reflected those observed experimentally: an initial growth of *Sphingomonas* Leaf257, a relative decline in *Sphingomonas* Leaf257 as *Rhizobium* Leaf68 began to grow, and subsequent fluctuations between the two population sizes. Moreover, while most of these simulations (22 out of 29, Fig. 3d, e) eventually led to a dampening of the fluctuations, a subset resulted in sustained oscillations of the two populations.

Beyond confirming the possibility that metabolic complementation can drive oscillatory population dynamics between environmental strains, the simulations allowed us to determine the metabolic factors driving these temporal effects. We compared the uptake of each metabolite by *Rhizobium* Leaf68 to its growth in each of the scenarios that resulted in coexistence. We calculated the cross-correlation of the time-resolved biomass flux of *Rhizobium* Leaf68 to its degradation of xylan, as well as to its uptake of xylose, alanine, acetate, dethiobiotin, niacin, and pantothenate derived from *Sphingomonas* Leaf257. This analysis indicated that, in agreement with our experimental observations, the growth of *Rhizobium* Leaf68 relies on all these carbon sources, except acetate (Fig. 3f). Despite these dependencies, we noticed an even stronger coupling of the growth of *Rhizobium* Leaf68 to the vitamins or vitamin precursors supplied by *Sphingomonas* Leaf257—with all simulations resulting in cross-correlation coefficients of 1.0 between the biomass flux of *Rhizobium* Leaf68 and its uptake of dethiobiotin, niacin, and pantothenate, respectively. This in silico analysis, therefore, suggests that while there is flexibility in the carbon sources that *Rhizobium* Leaf68 can use, its coexistence with *Sphingomonas* Leaf257 is tightly coupled to the cofactors it receives.

Taken together, these simulations shed light on specific time-dependent mechanisms that may underly the observed fluctuations between *Sphingomonas* Leaf257 and *Rhizobium* Leaf68 in vitro. The initial growth of *Sphingomonas* Leaf257 and the resulting output of carbon and—critically—vitamins enable *Rhizobium* Leaf68 to begin growing. *Rhizobium* Leaf68 then outgrows *Sphingomonas* Leaf257, leading to the decline of the latter. However, this reduction in *Sphingomonas* Leaf257 leads to exhaustion of vitamins, slowing of growth of *Rhizobium* Leaf68 and allowing the *Sphingomonas* Leaf257 population to resurge. This coexistence outcome is largely robust to initial conditions (Supplementary Fig. 11). This system thus provides evidence of specific carbon and vitamin-driven feedbacks between the two strains, which can enable their coexistence and shape community growth dynamics. These findings align with recent work demonstrating that metabolic cross-feeding can drive self-sustained population oscillations in engineered microbial communities^32^, further suggesting that similar dynamical principles – such as resource-mediated feedback between producer and consumer populations – can emerge among environmental microbes.

### Exogenous provision of metabolic mediators interrupts interactions

**Fig. 4:**
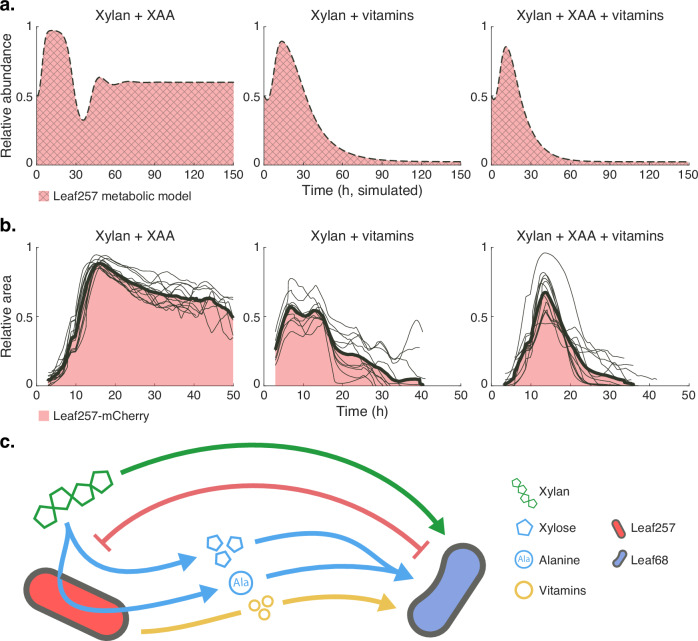
Prediction and validation of population steady states in mixed chambers between *Sphingomonas* Leaf257 and *Rhizobium* Leaf68. **a** Predicted relative abundance of *Sphingomonas* Leaf257 and *Rhizobium* Leaf68 from metabolic modeling simulations in three medium conditions: xylan + D-xylose, L-alanine, and acetate (XAA), xylan + pantothenate, niacin, and biotin (“vitamins”), and xylan + XAA + vitamins. Time-resolved profiles of extracellular metabolites in each of the corresponding simulations, as well as for the xylan-only condition (Fig. 3e), are reported in Supplementary Fig. 12. **b** Experimentally observed relative microchamber area of fluorescently labeled *Sphingomonas* Leaf257 inoculated at equal abundance with *Rhizobium* Leaf68 in four medium conditions: xylan + XAA (left, *N* = 13 microchambers, Supplementary Video 10), xylan + vitamins (middle, *N* = 11 microchambers, Supplementary Video 11), and xylan + XAA + vitamins (right, *N* = 13 microchambers, Supplementary Video 12). Thick lines represent the mean relative area of *Sphingomonas* Leaf257 over time. In all three pairings, growth of *Rhizobium* Leaf68 began after the initial filling of the chamber by *Sphingomonas* Leaf257. **c** Conceptual model of xylan-derived metabolic exchange and dynamic feedback between *Sphingomonas* Leaf257 and *Rhizobium* Leaf68, illustrating complementation (blue and yellow arrows), and mutual suppression (red flat-capped arrows). Source data are provided as a Source Data file.

The above-described simulations of interaction dynamics (Fig. 3d) indicated that modulation of specific metabolite abundances could alter ecological outcomes and stable states. We therefore extended our application of metabolic models to simulate distinct scenarios in which different molecular mediators were provided exogenously in addition to xylan: (1) equal amounts of xylose, alanine, and acetate (hereafter “XAA”), (2) biotin, niacin, and pantothenate, and (3) XAA with these three vitamins (Fig. 4). In doing so, we aimed to determine whether decoupling *Sphingomonas* Leaf257 and *Rhizobium* Leaf68 via specific components could alter the course of their interaction.

These simulations predicted the emergence of two distinct steady states. First, the exogenous supply of XAA (i.e., the carbon sources made available by *Sphingomonas* Leaf257) caused an initial increase of *Sphingomonas* Leaf257, followed by its decline and stabilization at an approximate relative abundance of 0.5, suggesting a possibility of sustained coexistence with *Rhizobium* Leaf68 (Fig. 4a). When vitamins were supplied, however, *Rhizobium* Leaf68 dominated and excluded *Sphingomonas* Leaf257 from the system. This outcome occurred irrespective of the exogenous supply of additional carbon sources, which, together with an analysis of the predicted concentration of these metabolites throughout the simulations (Supplementary Fig. 12), suggested that supplying vitamins could fully decouple the strains and interrupt the interaction.

To validate these predictions experimentally, we introduced the corresponding medium conditions into microfluidic devices loaded with *Rhizobium* Leaf68 and a fluorescently labeled *Sphingomonas* Leaf257. The three resulting observed steady states (Fig. 4b, Supplementary Videos 10–12) aligned with the simulations, highlighting cofactor complementation as a critical determinant for the emergence of coexistence between these strains in the context of oligosaccharide degradation (Fig. 4c).

## Discussion

Resolving the factors that underly the emergence and dynamics of microbial interactions remains challenging for host-associated and environmental ecosystems. Coupled with a synthetic community approach, microfluidic technologies represent powerful tools to identify the molecular mechanisms that drive interactions^25,33–38^. The device applied here enabled a space- and time-resolved monitoring of interaction outcomes otherwise unattainable through standard in vitro techniques, making it a flexible tool to identify metabolites critical to interactions between environmental microbes. The incorporation of an interface to separate two different populations allowed us to alleviate the need for fluorescent labeling of the organisms to discover interactions, as well as to attribute the proliferation of the interactor strains to diffusible metabolic mechanisms.

By applying this platform to explore metabolic exchanges stemming from plant oligosaccharide degradation, we had expected to discover trophic cascades mediated chiefly via the release of sugar monomers. While the degradation of xylan indeed led to increased abundance of xylose by the representative strain *Sphingomonas* Leaf257, its additional provision of alanine and acetate to recipient strains underscored a more complex pattern of carbon flux. Moreover, although the primary structure of the xylan molecule we used is representative of that found in *Arabidopsis*^20^, it lacks additional sidechains (e.g., glucuronic acid) that may provide even more opportunities for metabolic exchange in situ. Nonetheless, the complementation of vitamin auxotrophies—which, in the case of *Rhizobium* Leaf68, enabled it to utilize the oligomer itself—revealed further metabolic complexities that challenge a simple model of linear trophic chains between phyllosphere bacteria. Indeed, the high prevalence of vitamin auxotrophies in *Arabidopsis* leaf-associated bacteria^15^ paired with the widespread ability to digest complex plant-derived molecules (Supplementary Fig. 1) suggests that the phyllosphere may harbor relatively few strictly hierarchical trophic relationships between its members. Such a structure would contrast with those described for other host-associated and environmental microbial communities^13,16,39^, and possibly arises from the high degrees of spatial heterogeneity *in planta* and the role of plant-specific metabolites in structuring the microbiota^40–42^.

By leveraging information on the organism-specific metabolic exchange profiles we observed in our experiments, we used computational models to contextualize the emergence of fluctuating dynamics and metabolic feedback mechanisms. While oscillatory patterns have previously been described for microorganisms engaging in predator-prey (following classical Lotka-Volterra principles) or cheat-cooperator interactions^43,44^, as well as for engineered microbiota^32,45^, the possibility that such dynamics could arise purely from metabolic complementation among environmental microbes has remained unresolved. Our modeling approach recapitulated these oscillatory dynamics, showing how the combination of growth rates and complementation of carbon and vitamins could result in drastically different population steady states. These simulations further allowed us to quantify the sensitivity of the growth of *Rhizobium* Leaf68 to the flux of resources from *Sphingomonas* Leaf257, highlighting the impact of time delays between the activity and growth of the two strains in enabling lasting coexistence^46,47^. Though it remains challenging to precisely recapitulate the experimentally observed period and amplitude of the fluctuations in silico, these insights have allowed us to frame and validate predictions on the effects of modulating specific interaction features, highlighting the vulnerability of species coexistence to environmental composition^48–50^. More broadly, it provides a framework for understanding how multiple components of microbial interactions give rise to emergent dynamics and community stability—critical factors for engineering beneficial microbial communities to support host and environmental health^51^.

Our results on vitamin complementation also prompt questions about the broader ecological role of organisms like *Sphingomonas* Leaf257 in the phyllosphere. As a resource generalist that provides non-specific benefits to interacting bacteria, while simultaneously experiencing a reduction in its abundance in situ from the interactors^9,22^—*Sphingomonas* Leaf257 may function as a modulator of community composition. This strain and other *Sphingomonas* spp. have been shown to colonize *Arabidopsis* leaves consistently in community contexts^27,28^, and members of this genus have been shown to impact community assembly and structure^28^. As such, it may be that the persistence of *Sphingomonas* Leaf257 is safeguarded by a widespread dependency of other microbes on the metabolic products it generates. Furthermore, as suggested by an additional analysis (Supplementary Fig. 13) and previous work^15^, secretion of these products at rates that can sustain an interactor may not necessarily impose a high fitness cost on the producing organism. Indeed, as only sub-micromolar concentrations of vitamins could support the growth of the interactor strains in our study, even low-rate mechanisms such as passive leakage or cell lysis may suffice to sustain such an interaction when the interacting strains are in close spatial proximity. This possibility highlights the ecological importance of organisms capable of both degrading complex plant-derived molecules (Supplementary Fig. 1) and providing essential cofactors in shaping microbial communities. Identifying additional organisms with such a combination of traits is thus likely to provide further insights into the complexity of environmental microbiomes and of the interaction networks that shape their composition.

Lastly, though our study focused exclusively on commensal bacteria, the nutritional strategies we observed also parallel those employed by phytopathogens. While the commensal *Sphingomonas* strain in our study has previously been shown to access such molecules through competition-induced enzyme production^22^, opportunistic pathogens—such as those of the genus *Xanthomonas*—can constitutively secrete cell-wall degrading enzymes and can exploit weakened host defenses^23^. Beyond their ability to degrade xylan via type II secretion systems^52–55^, a recent study showed that a member of this genus can also deploy type III effectors that reprogram host transcription to release cell wall-bound sugars^56^. This convergence suggests that accessing plant cell wall carbohydrates may represent a fundamental bacterial strategy spanning the commensal-pathogen spectrum, highlighting the complex roles of these molecules in metabolic interactions.

## Methods

### Microfluidic device design and fabrication

The microfluidic device design is based on existing architectures^25,26,34^. It features an array of 200 units, each of which comprise two microchambers (each with a height of 1 µm and width of 40 or 50 µm) that are connected by an array of nanochannels 0.4 µm in width, 3 µm in length and with 2 µm spacing (Supplementary Fig. 2). A chamber height of 1 µm was chosen to enable entry and trapping of average-sized bacteria, while the chamber width and length of 40 or 50 µm was determined to be large enough to capture a small population of cells while minimizing metabolite gradients that could introduce significant non-uniformities^57,58^. Each side of the units is connected by independent, larger channels (100 µm width and 20 µm height) for cell injection and constant perfusion of media. This constant perfusion of media leads to a steady state of metabolites within the chambers and acts to continuously wash out any cells pushed out of each chamber as a result of population growth^25,34^.

Microfluidic devices were fabricated by standard photolithography and replica molding methods. Briefly, layers of SU8-2001 and SU8-2025 (Microchem) were spun on a clean silicon wafer, baked and exposed through direct laser writing (Heidelberg DWL66+). Patterned silicon wafers were then used as molds to cast polydimethylsiloxane (PDMS, Dow Corning) and fabricate the devices. PDMS was mixed in a 10:1 ratio with curing agent, degassed for 10 min, and poured on patterned silicon wafers. After curing for 1 h at 80 °C, PDMS layers were peeled off the silicon mold, cut, and punched for inlets and outlets with 1.5 mm-diameter biopsy punchers. Glass slides and PDMS layers were briefly cleaned with isopropanol and exposed to air plasma for 30 s in a plasma oven (Diener Electronic GmbH). After bringing surfaces into contact, bonded devices were heated to 80 °C for 1 min and stored until use.

### Selection and pre-cultivation of phyllosphere strains

Bacterial isolates from the *Arabidopsis thaliana* phyllosphere^24^ were selected to generate a representative phylogenetic cross-section based on high individual relative abundances in community settings ( ≥ 10%: *Rhizobium* Leaf68 and *Microbacterium* Leaf203, ≥ 1%: *Arthrobacter* Leaf145 and *Pseudorhodoferax* Leaf265, ≥ 0.1%: *Sphingomonas* Leaf5 and *Aeromicrobium* Leaf289)^27,28^ and/or previously observed interactions in situ (*Sphingomonas* Leaf5, *Rhizobium* Leaf68, *Arthrobacter* Leaf145, *Microbacterium* Leaf203, *Pseudorhodoferax* Leaf265, and *Aeromicrobium* Leaf289)^28^. In addition to engaging in a previously observed interaction in situ with Leaf68^22^, *Sphingomonas* Leaf257 was selected as a confirmed xylan degrader^22^ and a representative member of the Sphingomonadaceae, a common bacterial family on *A. thaliana* leaves whose members have plant beneficial functions^8,24,59–61^. *Sphingomonas* Leaf17 was selected as an additional member of the *Sphingomonas* genus, and *Pseudomonas* Leaf58 was selected as a representative member of the Gammaproteobacteria.

Bacterial isolates were streaked from glycerol stocks stored at −80 °C onto R-2A agar (Sigma Aldrich) supplemented with 0.5% (v/v) methanol and incubated for three days at 22 °C. Colonies were then suspended (1 µL loopful) into 2 mL of liquid R-2A broth (Neogen) supplemented with 0.5% (v/v) methanol in 10 mL culture tubes and incubated for 18 h at 28˚C with shaking at 180 rpm and tubes angled at 45˚. Cultures were washed three times by centrifuging for 3 min at 6000 x *g* at 22 °C and resuspending in a minimal medium with no carbon source. One liter of the minimal medium contained 2.4 *g* K_2_HPO_4_ (AppliChem), 1.08 *g* NaH_2_PO_4_·2H_2_O (Sigma Aldrich), 1.62 *g* NH_4_Cl (Sigma Aldrich) and 0.2 *g* MgSO_4_·7H_2_O (Sigma Aldrich). The medium was supplemented with the following trace elements: 15 mg Na_2_EDTA·2H_2_O, 3 mg FeSO_4_·7H_2_O, 4.5 mg ZnSO_4_·7H_2_O, 3 mg CoCl_2_·6H_2_O, 0.64 mg MnCl_2_, 1 mg H_3_BO_3_, 0.4 mg Na_2_MoO_4_·2H_2_O, 0.3 mg CuSO_4_·5H_2_O, and 3 mg CaCl_2_·2H_2_O. All media components were prepared with Milli-Q quality water (Millipore). The bacteria were then inoculated into a minimal medium containing 1.32 g/L xylan (Roth, ≥95% xylooligosaccharides from corncob, MW: (132)_n_ g/mol, catalog no.: 8659, lot no.: 089281125) (hereafter MM + xylan) and incubated for 24 h at 28 °C with shaking at 180 rpm and tubes angled at 45˚. The OD_600_ of each culture was then normalized to 0.2 before inoculation into the microfluidic devices.

### Loading and time-lapse imaging of bacterial growth in microfluidic device

Cell suspensions were injected into the corresponding inlet of the microfluidic device. For assaying co-culture dynamics, cells were mixed at a 1:1 ratio immediately before injection into the same inlet of the microfluidic device. After injection, the inlet wells were washed with fresh MM + xylan and Tygon tubings were connected to supply a constant perfusion of medium at 0.1 mL/hr through a syringe pump (Harvard Apparatus). For experiments in which additional carbon sources or vitamins were supplied, the medium consisted either of 1.32 g/L xylan with 10 mM each of D-xylose, L-alanine, and acetate (Xylan + XAA), 1.32 g/L xylan with 1.05 µM D-pantothenic acid hemi calcium salt, 0.41 µM biotin, 1.19 µM thiamine HCl, and 1.22 µM nicotinic acid (Xylan + vitamins), or 1.32 g/L xylan with the aforementioned carbon sources and vitamins (Xylan + XAA + vitamins).

For all experiments, microfluidic devices were loaded onto the stage of a Nikon Ti2 microscope and imaged at 40x magnification at 28 °C. Illumination was applied through an LED light source for transmitted light microscopy or a Nikon Intensilight fluorescent lamp for fluorescent microscopy. Unless otherwise stated, image acquisition was performed every 10 min through a photocamera (Hamamatsu) set at 30 ms and 100 ms exposure time for transmitted and fluorescent light, respectively. Any instance in which de-bonding of the microfluidic device was observed was considered a device failure, and any associated data was discarded.

### Image analysis

Images were cropped, rotated, and threshold adjusted as necessary to isolate one microchamber using ImageJ. For quantification of cell counts in the interaction screen, MIDAP^62^ was used to segment and detect individual bacteria. To determine the relative area occupied by the fluorescently labeled *Sphingomonas* Leaf257 in each chamber, image stacks were converted to HDF5 format using a custom Python script. Here, image segmentation was performed using ilastik (v1.0)^63^. The “simple pixel classification” model was trained on at least three fields of view selected from five randomly chosen chambers. The model was trained to detect fluorescent cells. Following training, the predefined workflow in ilastik was applied to batch-process and binarize the extracted chamber image stacks using “simple segmentation.” Finally, the cell area fraction was calculated using a custom Python script. Distributions of cell counts (Fig. 1c) were visualized using violin plots^64^. The variation in relative area stemming from the three rounds of model training is shown in Supplementary Fig. 14.

### Collection of effluent from microfluidic device and cultivation of interactor strains

Suspensions of *Sphingomonas* Leaf257 were pre-cultured as above and loaded into one side of 12 separate microfluidic devices. Syringe filters (0.22 µm) were fitted to the outlet tubing of each device, and a constant flow-through of MM + xylan was applied at 0.1 mL/hr at 28 °C. Effluent was collected into sterile 50 mL tubes and either used immediately for cultivation of interactor strains or stored at −20 °C for NMR and LC-MS analysis. Interactor strains (*Rhizobium* Leaf68, *Microbacterium* Leaf203, and *Pseudorhodoferax* Leaf265) were pre-cultured as above and inoculated at an OD_600_ of 0.05 into 10 mL culture tubes containing 2 mL of either MM + xylan or *Sphingomonas* Leaf257-treated microfluidic effluent. Cultures were incubated for 36 h at 28 °C with shaking at 180 rpm and tubes angled at 45˚. At 17 and 36 h post inoculation, 500 µL of the culture was removed and centrifuged at 6000 x *g* for 3 min. The supernatant was aspirated, filter sterilized (0.22 µm), and stored at −20 °C for NMR and LC-MS analysis.

### NMR spectroscopy and analysis

Samples consisting of 300 μL of culture media were mixed with D_2_O (99.8%, Merck, 617385-1-107KG) containing 1 mM DSS-D6 (98%, CIL DLM-8206-1). This mixture resulted in a final DSS proton concentration of 100 μM. The prepared solutions were transferred to 3 mm NMR tubes, suitable for use with the SampleJet system (Z168406, Bruker Inc.).

NMR data were acquired at 298.0 K at a 600 MHz Bruker AVIII Avance HD NMR spectrometer equipped with a CP-QCI-1H/19F-13C/15N-2H 05 Z cryogenically cooled probe. The system included a SampleJet (Bruker Inc.) sample handling robot for automation, with the samples stored at 278 K between the experiments. 1D ¹H experiments were acquired with 65536 complex data points and 2048 transients. The pre-scan relaxation delay was set to 3.5 s. The solvent signal was suppressed by applying pre-saturation during the pre-scan delay and by applying a short (10 ms) NOE mixing period followed by a z-gradient before the final π/2 pulse. Data were zero-filled to 131072 points, and a squared cosine window function was applied. The resulting spectra were baseline-corrected before peak integration.

Data acquisition and processing were performed using TopSpin 3.6 (Bruker Inc.). Peak integrals of metabolites were normalized to the integral of DSS at 0 ppm for concentration determination. Metabolite identification was conducted by manual comparison to metabolite standards for D-xylose and L-alanine, as well as by using AssureNMR 2.2 (Bruker Inc.) in conjunction with the BBIOREFCODE SBASE metabolite spectral database (Bruker Inc., version 2.01) for other metabolites. Peak integrals and metabolite concentration calculations are reported in Supplementary Data 1, and NMR data are available through the ETH Zurich Research Collection at doi.org/10.3929/ethz-b-000737298. Distributions of detected metabolites (Fig. 2b) were visualized using violin plots^64^.

### LC-MS measurement and analysis

Standard solutions of biotin, dethiobiotin, niacin, pantothenate, and thiamine were prepared in a minimal medium containing 1.32 g/L xylan, at 20 different vitamin concentrations ranging from 500 nM to 0.01 nM. Microfluidic effluent and batch cultivation samples were measured using two different dilution approaches: 1:10 in Milli-Q grade water followed by 1:5 in acetonitrile (1:50), and 1:5 in acetonitrile (1:5).

Standard solutions and samples (5 μL for 1:50 diluted samples and 20 µL for the 1:5 diluted samples) were measured within the same acquisition batch in a randomized order. Measurements were performed using a Thermo Ultimate 3000 UHPLC system (Thermo Fisher Scientific). Separation was achieved using an Atlantis Premier BEH Z-HILIC column (2.1 mm × 100 mm, 1.7 µm particle size with VanGuard, Waters). Mobile phase A was 15 mM ammonium bicarbonate at pH 9 in 40/60 acetonitrile/water (v/v), and mobile phase B was 15 mM ammonium bicarbonate at pH 9 in 90/10 acetonitrile/water (v/v). The following gradient was applied: first, the flow rate was set to 0.5 mL/min. From 0 to 5 min, B was decreased in a slightly concave manner (curve = 6) from 82% to 37%. From 5 to 7 min, B was hold at 37%. From 7 to 7.5 min B was increased back to the initial condition (82%). From 7.5 to 8.5 min, the flow rate was increased to 0.8 mL/min and kept until 12.5 min. From 12.5 to 12.6 min the flow rate was decreased back to the initial condition (0.5 mL/min), which was maintained until the end of the gradient at 13 min. For mass analysis, the LC instrument was coupled to a Thermo QExactive Plus instrument (Thermo Fisher Scientific), which was operated in the positive mode using a PRM approach with the following parameters: mass resolution: 17,500; AGC target: 5 × 10^6^; Isolation window: 0.6 m/z; maximum injection time: 100 ms (0–2 min), 500 ms (2-8 min). Heated electro-spray ionization probe was used applying the following source parameters: vaporizer 250 °C; aux gas 20; ion spray voltage +3.00 kV, sheath gas 50; sweep gas 0; radio frequency level 50.0; capillary temperature 275 °C.

LC-MS data were analyzed using custom Python scripts within the emzed3 environment^65^. Measurements of vitamin standards at different concentrations were used to generate calibration curves for each compound using a linear fit based on the peak areas of the extracted ion chromatograms. These calibration curves were used to estimate absolute vitamin concentrations in each sample. Compounds whose estimated concentrations fell below the lowest measurable concentration of the corresponding standard were considered to be undetected. Quantification results are reported in Supplementary Data 2, and LC-MS data and analysis scripts are available through Zenodo^66^. Distributions of detected metabolites (Fig. 2b) were visualized using violin plots^64^.

### Metabolic modeling

Genome-scale metabolic models for *Sphingomonas* Leaf257 and *Rhizobium* Leaf68^9^ were further curated to reflect the physiological properties (i.e., vitamin auxotrophies, growth rates, and carbon conversion capabilities) examined in this study. In addition, gene-protein-reaction (GPR) rules for central carbon metabolism were manually curated in both models to ensure accurate representation of enzyme complexes. Specifically, the model for *Sphingomonas* Leaf257 was modified to be able to hydrolyze xylan extracellularly, to secrete D-xylose, L-alanine, and acetate, and to produce and leak pantothenate, niacin, and dethiobiotin to simulate the appearance of these vitamins in the medium. Output rates for the three carbon sources were approximated by first setting an upper bound of 0.31 hr^−1^ to the biomass reaction based on the maximum growth rate of Leaf257 assayed in vitro (Supplementary Fig. 15). A simulated medium with xylan as sole carbon source was then applied, and ratio reactions were added to couple the secretion of D-xylose, L-alanine, acetate, niacin, pantothenate, and dethiobiotin at ratios of 2: 10, 1: 10, 3: 10, 4 × 10^−5^: 10, 180 × 10^−5^: 10, and 5 × 10^−5^: 10, respectively to the uptake of xylan-derived xylose monomers, in order to reflect the output ratios observed experimentally (Fig. 2c). Growth optimization was then carried out with flux balance analysis using the COBRA Toolbox^67^ in MATLAB R2021a (Mathworks), which yielded predicted rates of secretion for each of these metabolites. These were incorporated into the model as lower bounds of secretion for each metabolite, namely 1.62 mmol/gDW/hr for D-xylose, 0.81 mmol/gDW/hr for L-alanine, 2.43 mmol/gDW/hr for acetate, 1.46 µmol/gDW/hr for pantothenate, 32.4 nmol/gDW/hr for niacin, and 4.05 nmol/gDW/hr for dethiobiotin. The upper bound on the growth rate and the ratio reactions for each secreted metabolite were then removed. The model for *Rhizobium* Leaf68 was similarly modified to be able to hydrolyze xylan to xylose, and vitamin auxotrophies were introduced based on previous studies^15,22^ either by eliminating genes relevant to their biosynthesis (pantothenate synthase PanC and ketopantoate hydroxymethyltransferase PanB for CoA, and quinolinate synthase NadA for NAD) or, in the case of biotin, adding biotin as a biomass precursor and confirming the absence of dethiobiotin synthase BioD. Secretion of metabolic products not observed experimentally was blocked for each model. Simulation of growth via flux balance analysis confirmed the inability of the *Rhizobium* Leaf68 model to produce biomass in a medium containing xylan but lacking vitamins.

The genome-scale models were then loaded into COMETS^31^ (v2.11.3) to assess emergent temporal dynamics. Here, a continuous culture without spatial structure was simulated, in which a constant influx of media was applied, and biomass was diluted via a constant death rate of 0.1 hr^−1^ for each model. Simulations were initialized with an initial biomass of 1.875 × 10^−10^ g, 10 mM xylan, and a medium refresh rate of 5 × 10^−9^ mmol/hr to approximate conditions in the microfluidic devices. Interactions were simulated with a time step of 0.05 hr, at each of which the biomass and extracellular metabolite abundances $M$ were updated based on maximum metabolite uptake rates ${V}_{\max }$, defined as

1 $${V}_{\max }={V}_{\max,d}\left[\frac{M}{M+{K}_{m}{W}^{3}}\right]$$

where ${V}_{\max,d}$ is the default maximum uptake rate for each metabolite (1000 mmol/gDW/hr), ${K}_{m}$ is the Michaelis constant (20 mmol/cm^3^), and ${W}^{3}$ is the volume of the simulation container (1 × 10^−6^ cm^3^). These values were chosen to operate within the linear range of Michaelis-Menten kinetics and to ensure physiologically realistic rates of xylose uptake (10^0^ – 10^1 ^mmol/gDW/hr) as simulated previously for monosaccharides^30,31^. Where applicable, the initial medium composition was modified to include 10 mM each of D-xylose, L-alanine, and acetate, and/or 1 µM each of pantothenate, niacin, and biotin. In each simulation, the time-resolved biomass quantities, fluxes, and extracellular metabolite abundances were collected. To explore alternative steady states between *Sphingomonas* Leaf257 and *Rhizobium* Leaf68 stemming from varied rates of carbon and vitamin output, a lower bound of CO_2_ secretion of 20 mmol/gDW/hr was first applied to the *Sphingomonas* Leaf257 to ensure physiologically realistic rates of respiration and reduce the potential for secretion of additional metabolic byproducts not observed experimentally. The secretion rates of D-xylose, L-alanine, acetate, pantothenate, niacin, and dethiobiotin by *Sphingomonas* Leaf257 were then fixed at different values (ranging from 0-100 times the above-named bounds for each metabolite) before running simulations in COMETS. The files for simulating and plotting population dynamics in COMETS, as well as simulation data, are available through Zenodo^68^.

### Reporting summary

Further information on research design is available in the Nature Portfolio Reporting Summary linked to this article.

## Supplementary information

Supplementary Information

Descriptions of Additional Supplementary Files

Supplementary Data 1

Supplementary Data 2

Supplementary Videos 1-12

Reporting Summary

Transparent Peer Review file

## Source data

Source Data

## Data Availability

The genome-scale metabolic models and computational simulation data generated in this study are available through Zenodo [https://zenodo.org/records/19187535]^68^. NMR data have been deposited in the ETH Zurich Research Collection at 10.3929/ethz-b-000737298. LC-MS data are available through Zenodo [https://zenodo.org/records/19188132]^66^. Source data are provided with this paper.

## Code availability

The files for simulating and plotting population dynamics in COMETS, as well as simulation data, are available through Zenodo [https://zenodo.org/records/19187535]^68^. Scripts for analyzing LC-MS data are available through Zenodo [https://zenodo.org/records/19188132]^66^.
