# Supplementary material for: Metabolic feedbacks drive population dynamics and can lead to oscillations among leaf bacteria
Source: Nat Commun. 2026 May 29;17:6983. doi: 10.1038/s41467-026-73686-w (PMC13392072; doi:10.1038/s41467-026-73686-w)
Supplement: Supplementary file 1 — Supplementary Information [file 41467_2026_73686_MOESM1_ESM.pdf]

**Supplementary Information for**

# Metabolic feedbacks drive population dynamics and can lead to oscillations among leaf bacteria

Alan R. Pacheco, Giovanni Stefano Ugolini, Simon H. Rüdisser, Andrea Zamuner, Miriam Bortfeld-Miller, Patrick Kiefer, Franziska Oschmann, Samuel G.V. Charlton, Michael Berger, Tommaso Redaelli, Miguel Ángel Salazar, Ilija Dukovski, Jan Roelof van der Meer, Olga T. Schubert, Martin Ackermann, Roman Stocker, and Julia A. Vorholt

Supplementary Figures

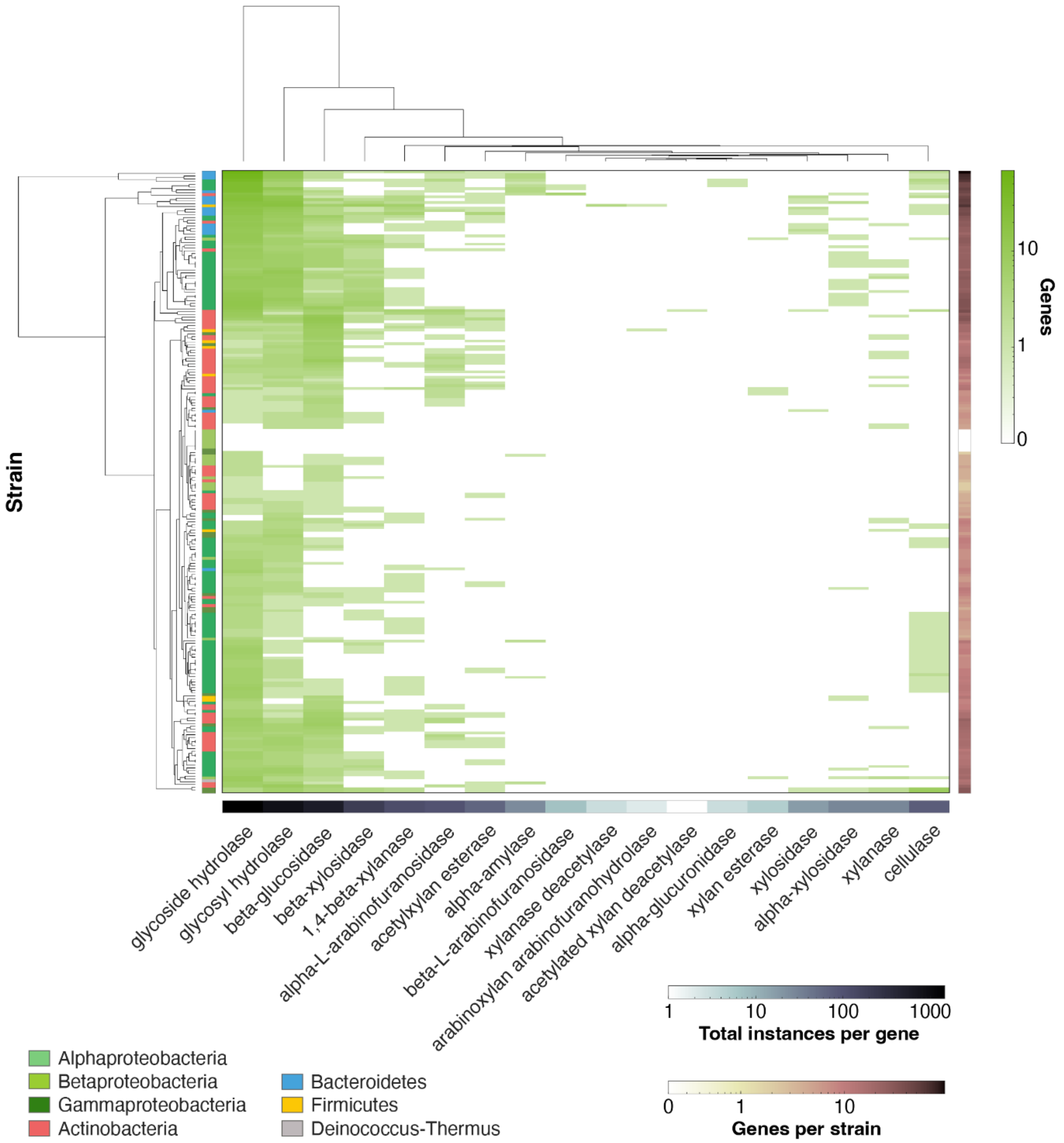


Supplementary Figure 1. Abundance of genes related to plant oligo- and polysaccharide degradation in representative *Arabidopsis* phyllosphere bacteria. Reported are the number of genes containing selected relevant keywords (e.g., ‘xylanase,’ ‘xylosidase,’ ‘acetylxylan,’ glucosidase,’ etc.) within genome annotations for 223 bacterial isolates in the *At*-LSPHERE collection[1] (NCBI BioProjects PRJNA297956 and PRJEB47672). Clustering was performed on the gene abundance matrix using the ‘clustergram’ function in MATLAB. The phylum/class of each strain is displayed on the y-axis.

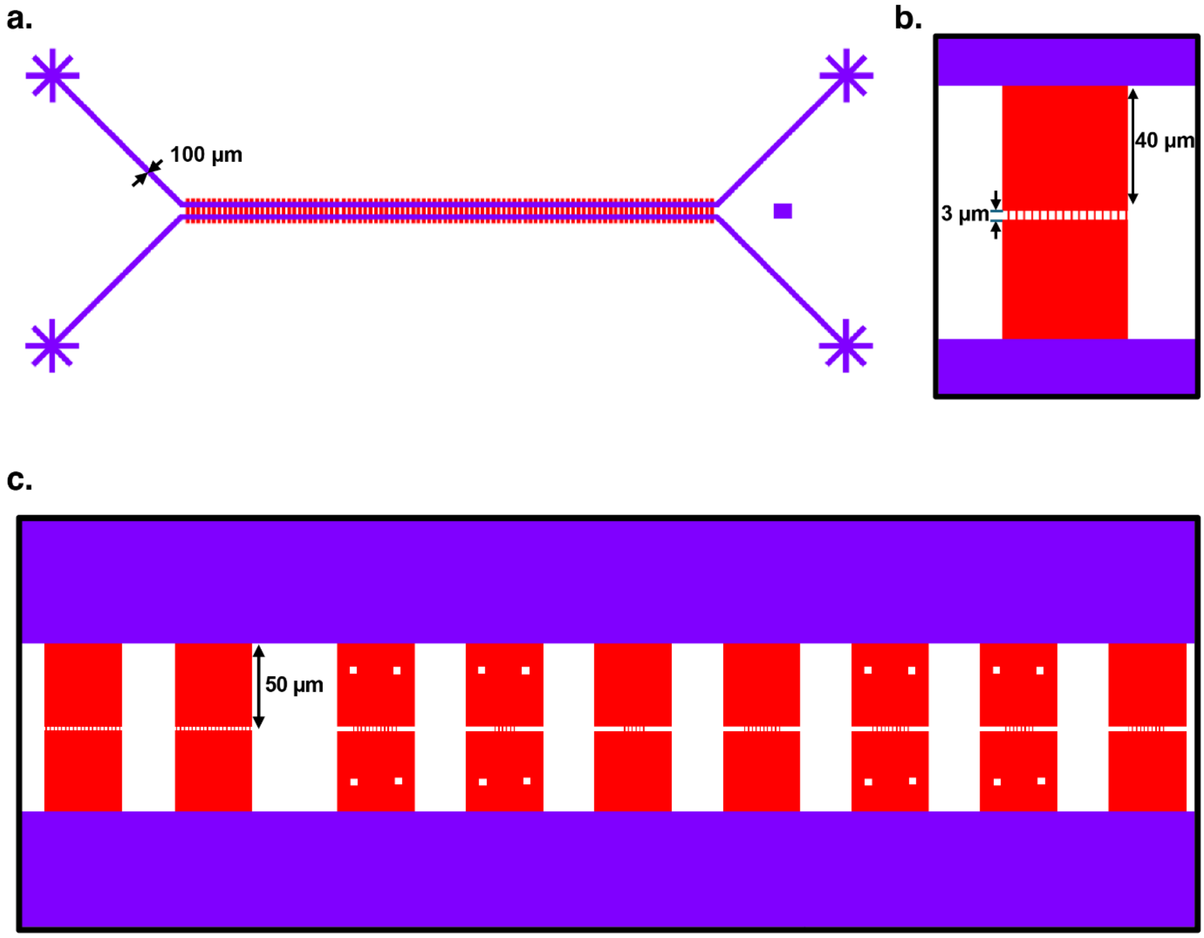


Supplementary Figure 2. General design of microfluidic device. **a.** An array of 200 adjacent microchambers (red, each with a height of 1 µm and width of 40 or 50 µm) is placed between two main channels (violet, 100 µm width, 20 µm height). A chamber height of 1 µm was chosen to enable loading and trapping of bacteria, while the width and length enabled capturing a small population of cells while minimizing resource gradients. **b.** Screening of interactions between *Sphingomonas* Leaf257 and other *A. thaliana* phyllosphere bacterial isolates was performed in devices featuring 40 × 40 × 1 µm microchambers connected by an array of nanochannels 0.4 µm in width, 3 µm in length, and with 2 µm spacing. **c.** Subsequent experiments featuring three selected interacting strains (*Rhizobium* Leaf68, *Microbacterium* Leaf203, and *Pseudorhodoferax* Leaf265) were performed using a device geometry consisting of 50 × 50 × 1 µm microchambers. Here, chambers with pores spanning the entire length of the chambers alternate with chambers featuring only limited number of pores (namely, 1, 2, 3, 4, 6, 10, or 12 pores). Square pillars (6 µm length × 6 µm width) were added to every other pair of chambers to prevent sagging of the chamber ceiling and collapse of the microchambers. Data was collected from chambers with and without these pillars, as they did not impact biological results. We collected data from chambers with 2, 4, and 6 pores to verify dependency of partner growth on diffusive compounds (Supplementary Figure 4) while we collected data from chambers with 2 or 3 pores for the experiments involving co-culture dynamics of interacting strains (Figure 3, Figure 4).

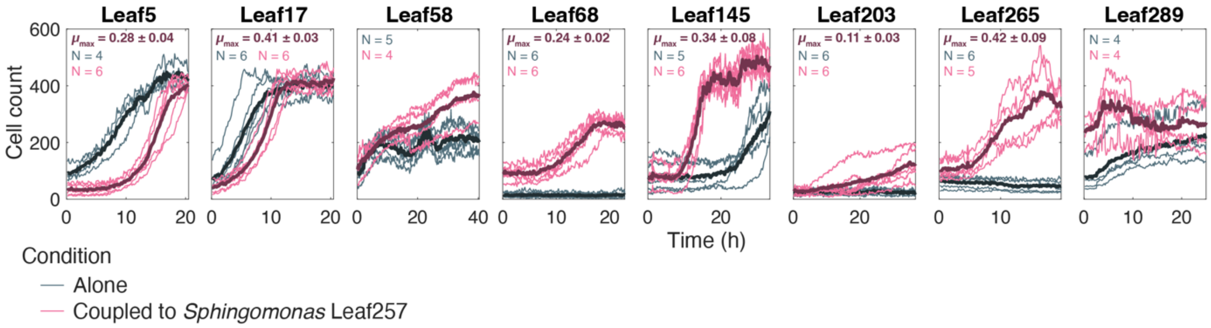


Supplementary Figure 3. Growth of *Arabidopsis* phyllosphere bacterial strains in microchambers uncoupled from ("Alone") or coupled to *Sphingomonas* Leaf257. Thick darker lines represent mean cell counts over time for each condition. Growth rates ($h^{-1}$) for strains coupled to *Sphingomonas* Leaf257 were calculated, where appropriate, by fitting each growth curve to a modified Gompertz growth model[2] using the Dashing Growth Curves tool[3] (Gompertz-tight). Quantity displayed denotes means and standard deviations of maximum growth rates across the replicate microchambers.

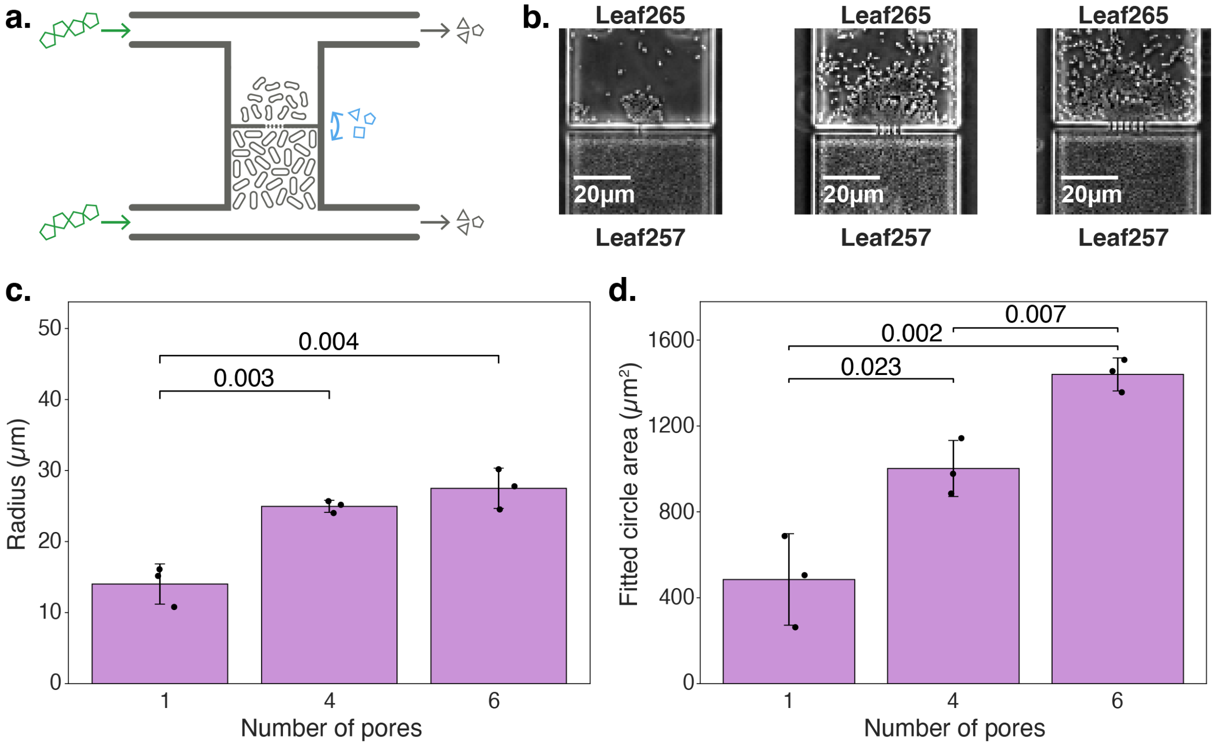


Supplementary Figure 4. Growth of *Pseudorhodoferax* Leaf265 is dependent on the degree of metabolic exchange with *Sphingomonas* Leaf257. **a.** Schematic of microfluidic device with narrower interface, which comprises fewer pores between the microchambers containing each strain. **b.** Representative interactions between the two bacterial populations (*Pseudorhodoferax* Leaf265, top; *Sphingomonas* Leaf257, bottom) at steady state (defined by the radius of the *Pseudorhodoferax* Leaf265 colony no longer expanding) for interfaces with one (left), four (middle) and six (right) pores, respectively. **c, d.** Estimated radii **(c)** and areas **(d)** of *Pseudorhodoferax* Leaf265 colonies at steady state according to number of pores. Estimates were generated by analyzing 10 frames in timelapses of colonies at steady state for three chambers per number of pores. Here, ilastik[4] was used to detect cells and define a contiguous region of growth. The optical flow of each contiguous region was calculated across all 10 frames, and the maximum distance of the detected profile of growing cells from the center of the pores was used to define colony radius. For area estimates, the smallest semicircle that covered the largest detected contiguous patch of cells was fitted to all colonies. Displayed are the means and standard deviations across 3 biological replicates, p-values of two-tailed t-tests are displayed for significant (< 0.05) comparisons.

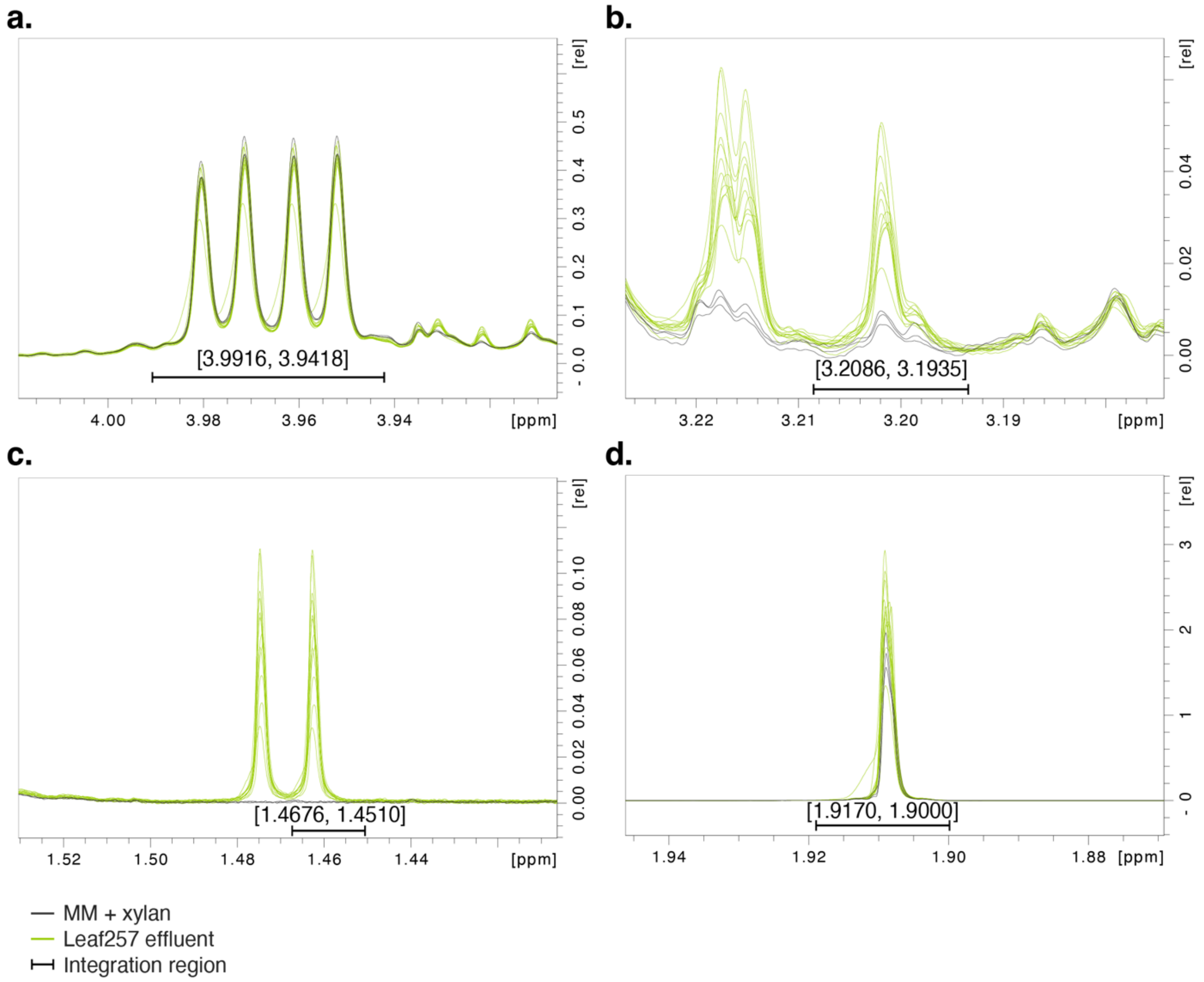


Supplementary Figure 5. Comparison of NMR spectra of compounds between the growth medium (MM + xylan, N = 3 biological replicates) and the effluent of *Sphingomonas* Leaf257 (N = 12 biological replicates) cultivated in the microfluidic device (Figure 2b). Shown are spectra of relevant integration regions for xylan **(a)**, xylose **(b)**, alanine **(c)**, and acetate **(d)**. Integration regions and integrals for all detected compounds are detailed in Supplementary Data 1, and all NMR spectra are provided through the ETH Zurich Research Collection at doi.org/10.3929/ethz-b-000737298.

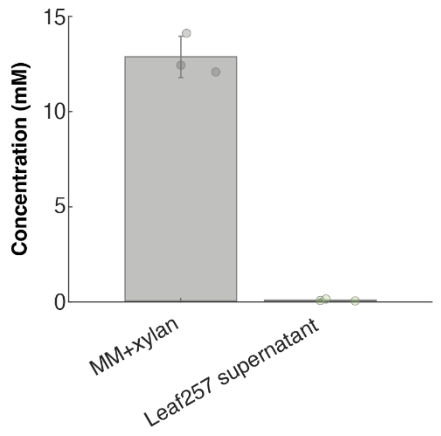


Supplementary Figure 6. NMR analysis of xylan degradation by *Sphingomonas* Leaf257. Samples were derived from filtered supernatants of *Sphingomonas* Leaf257 cultivated in minimal medium with 1.32 g/L xylan (10 mM of constituent monomers) for 24 hours (N = 3 biological replicates, bars denote mean ± s.d.). *Sphingomonas* Leaf257 cultures reached an $OD_{600}$ of 1.50 ± 0.05 (mean ± s.d.) at time of supernatant collection.

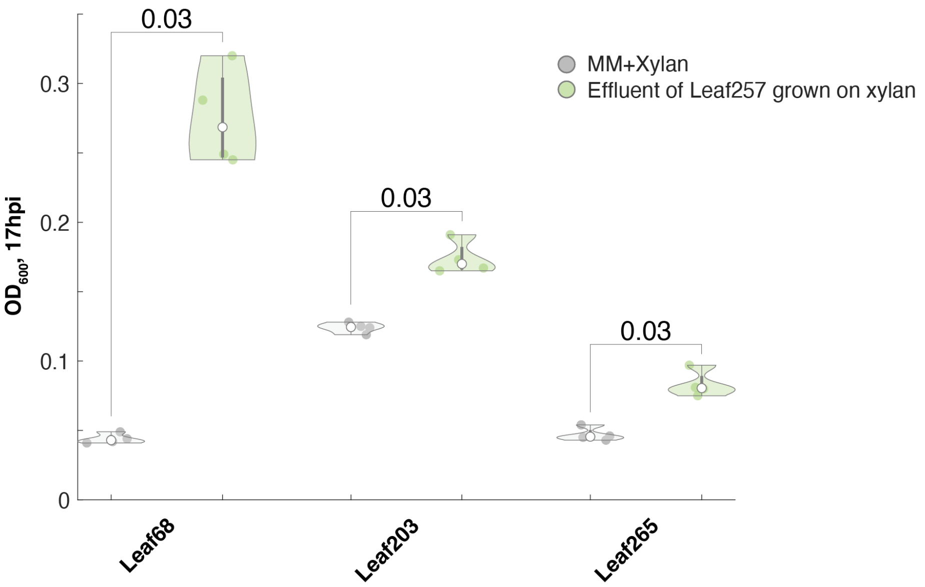


Supplementary Figure 7. Growth of interactor strains on *Sphingomonas* Leaf257 effluent as measured by optical density, 17 hours post inoculation. Four uninoculated effluents were cultivated simultaneously as negative controls, with final OD600 values of 0.0003 ± 0.0005 (mean ± s.d.). Violin plots display kernel density estimates of OD600 distributions with overlays of each data point[5,6]. Boxplots within each violin plot display the median (white circle), interquartile range (gray box), and upper/lower adjacent values (gray whiskers). P-values of one-tailed Mann-Whitney *U* tests are displayed for significant (< 0.05) comparisons across 4 biological replicates.

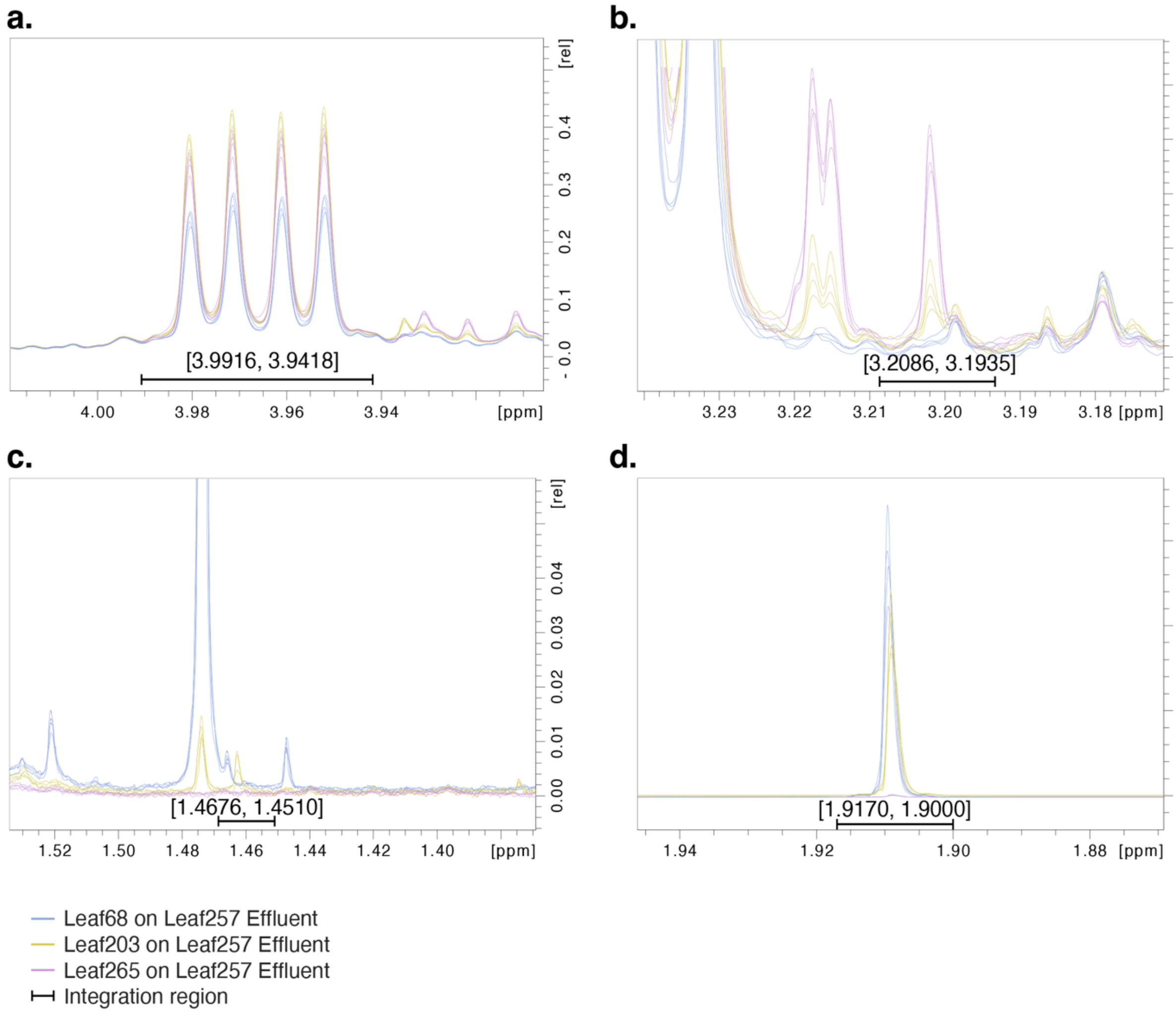


Supplementary Figure 8. NMR spectra of compounds in culture supernatants of *Rhizobium* Leaf68, *Microbacterium* Leaf203, and *Pseudorhodoferax* Leaf265 (N = 4 biological replicates for each) cultivated on the effluent of *Sphingomonas* Leaf257 harvested from microfluidic devices (Figure 2b). Shown are spectra of relevant integration regions for xylan **(a)**, xylose **(b)**, alanine **(c)**, and acetate **(d)**. Integration regions and integrals for all detected compounds are detailed in Supplementary Data 1, and all NMR spectra are provided through the ETH Zurich Research Collection at doi.org/10.3929/ethz-b-000737298.

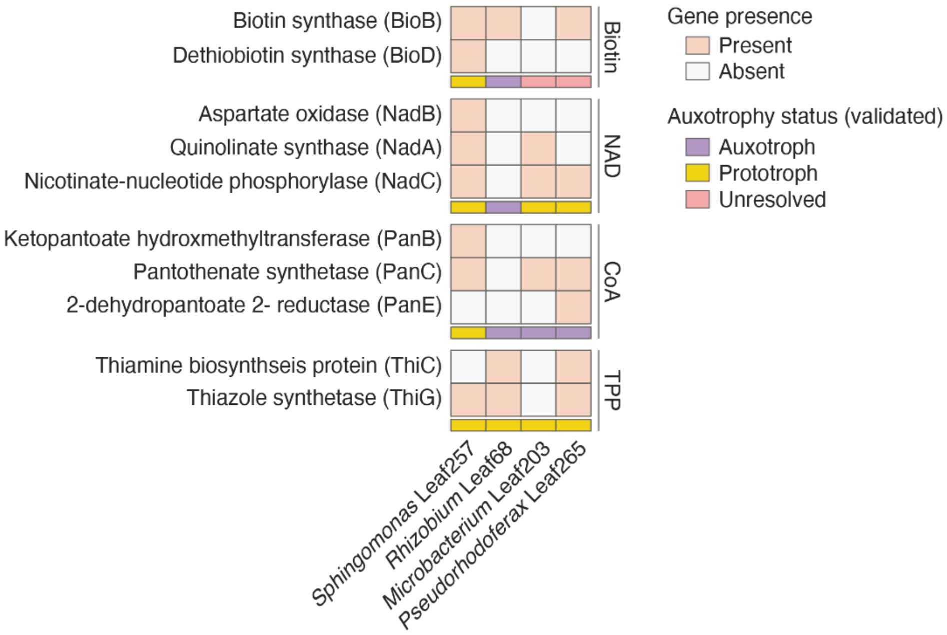


Supplementary Figure 9. Cofactor biosynthesis capabilities of interactor strains based on gene content and validation *in vitro*. Experimental assessment was carried out by pre-culturing each strain and washing the cultures (Methods), followed by a 10-fold dilution spotting of a 4 µL suspension of bacteria in 10 mM $MgCl_2$ onto solid media in biological quadruplicate. The agar plates were composed of 1.5% noble agar and a minimal medium composition (Methods), with either 1.32 g/L xylan or a mixture of 42 carbon sources (all sugars, amino acids, and organic acids described previously[7] except for methylamine, methanol, and coniferyl alcohol). The plates were additionally supplemented with either 1.05 µM D-pantothenic acid hemi calcium salt, 0.41 µM biotin, 1.19 µM thiamine HCl, or 1.22 µM nicotinic acid[8]. The presence or absence of a colony after a 96-hour incubation at 22˚C was used to determine each auxotrophy for each strain. Biotin auxotrophies for *Microbacterium* Leaf203 and *Pseudorhodoferax* Leaf265 remain unresolved given the appearance of colonies in this screen despite their lack of dethiobiotin synthase (BioD). This raises the possibility that, like *Rhizobium* Leaf68[8], they could divide for multiple generations without exogenous biotin or dethiobiotin. The residual growth of *Microbacterium* Leaf203 in minimal medium with xylan outside of the microfluidic device (Supplementary Figure 7) may be in line with this hypothesis, despite it not taking up dethiobiotin from *Sphingomonas* Leaf257 (Figure 2).

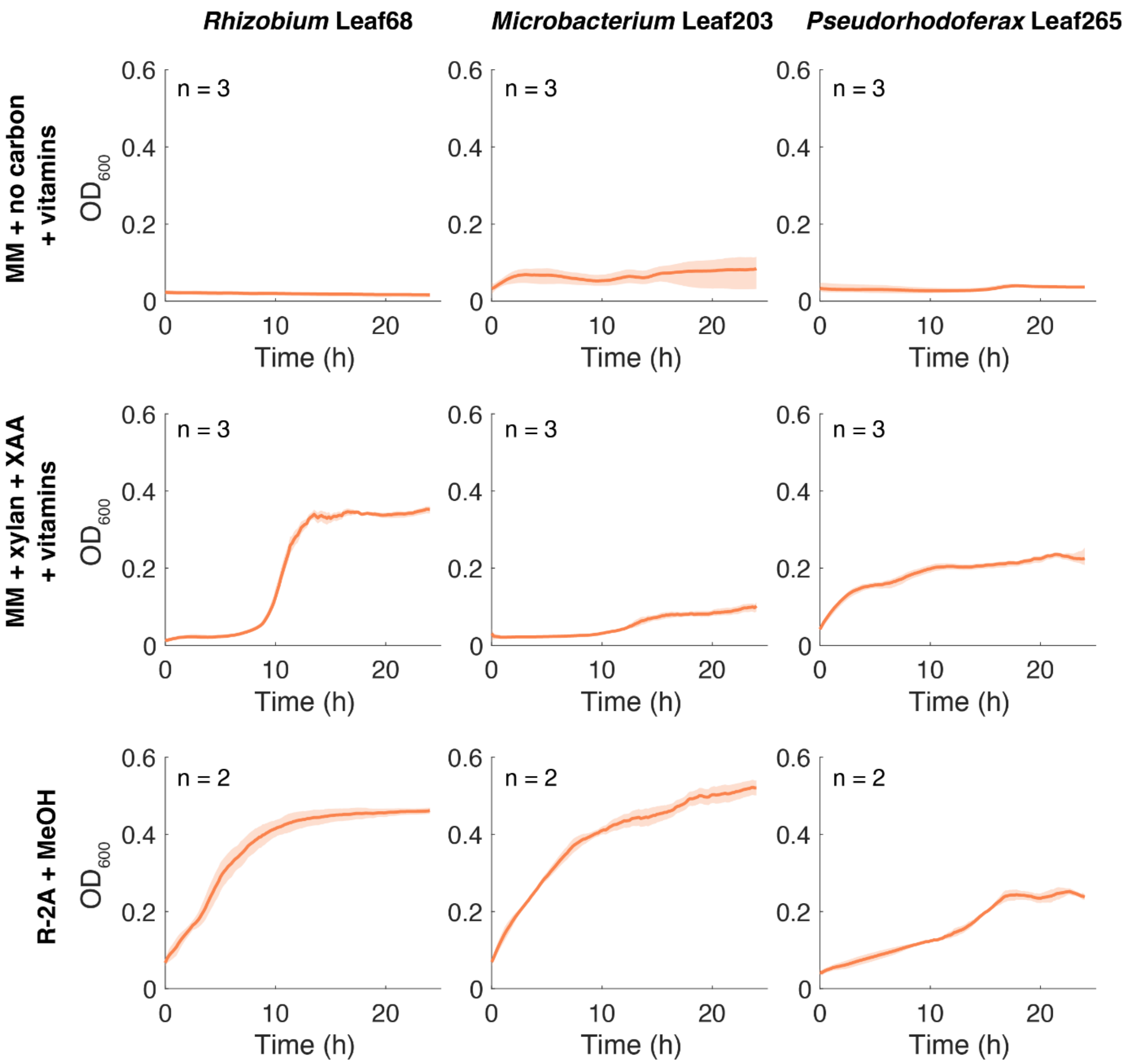


Supplementary Figure 10. Assessment of growth capabilities of interactor strains in metabolites stemming from xylan degradation by *Sphingomonas* Leaf257 in plate reader spectrophotometer (minimum and maximum displayed in light orange, mean growth displayed in dark orange). $OD_{600}$ values displayed were corrected using a blank measurement. Strains were cultivated at 28˚C with orbital shaking on a minimal medium containing either no carbon ("MM + no carbon," top), 1.32 g/L xylan with 10 mM each of D-xylose, L-alanine, and acetate, and vitamins (pantothenate, niacin, biotin, and thiamine) ("MM + xylan + XAA + vitamins," middle), or in R-2A broth supplemented with 0.5% (v/v) methanol ("R-2A + MeOH," bottom) as a standard rich medium for leaf bacterial isolates.

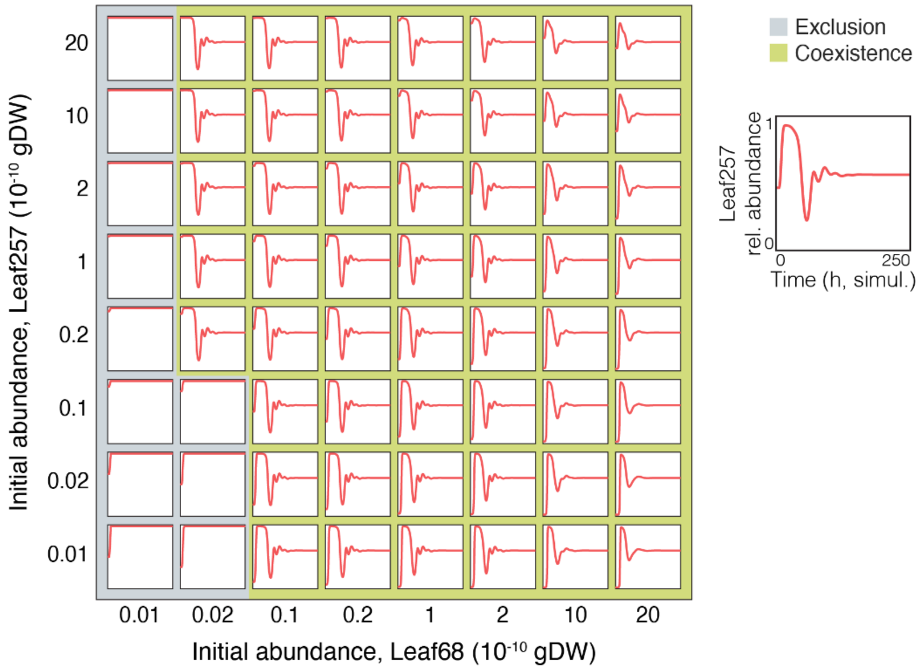


Supplementary Figure 11. Impact of varying initial abundances of *Sphingomonas* Leaf257 and *Rhizobium* Leaf68 in a simulated medium containing xylan as sole carbon source with no vitamins. Simulations were carried out for 250 h, where initial abundances of $2 \times 10^{-10}$ gDW correspond to the starting abundances used for simulations in Figure 3 and Figure 4.

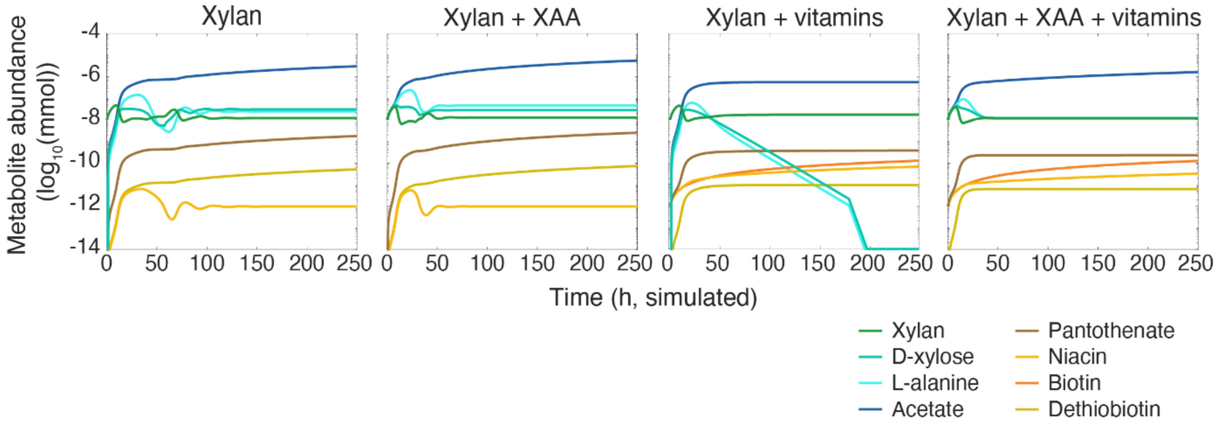


Supplementary Figure 12. Abundance of extracellular metabolites in metabolic modeling simulations of *Sphingomonas* Leaf257 with *Rhizobium* Leaf68 in four medium conditions: xylan, xylan + D-xylose, L-alanine, and acetate (XAA), xylan + pantothenate, niacin, and biotin ("vitamins"), and xylan + XAA + vitamins. The corresponding relative abundances of *Sphingomonas* Leaf257 are found in Figure 3e for the xylan condition, and in Figure 4a for xylan+XAA, xylan+vitamins, and xylan+XAA+vitamins.

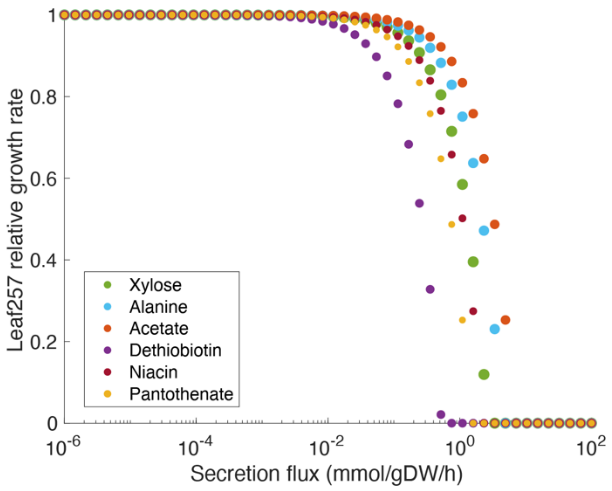


Supplementary Figure 13. Flux variability analysis of output of carbon sources and vitamins by the *Sphingomonas* Leaf257 metabolic model in a simulated medium with xylan as sole carbon source. A maximum xylan uptake flux of 0.66 mmol/gDW/hr was first set in order to constrain the biomass flux to 0.31 $h^{-1}$ (Supplementary Figure 15). The secretion fluxes of xylose, alanine, acetate, dethiobiotin, niacin, and pantothenate were then individually fixed at increasing values ranging from $1 \times 10^{-6}$ to $1 \times 10^{2}$ mmol/gDW/hr, and the resulting growth rates as calculated by flux balance analysis were recorded.

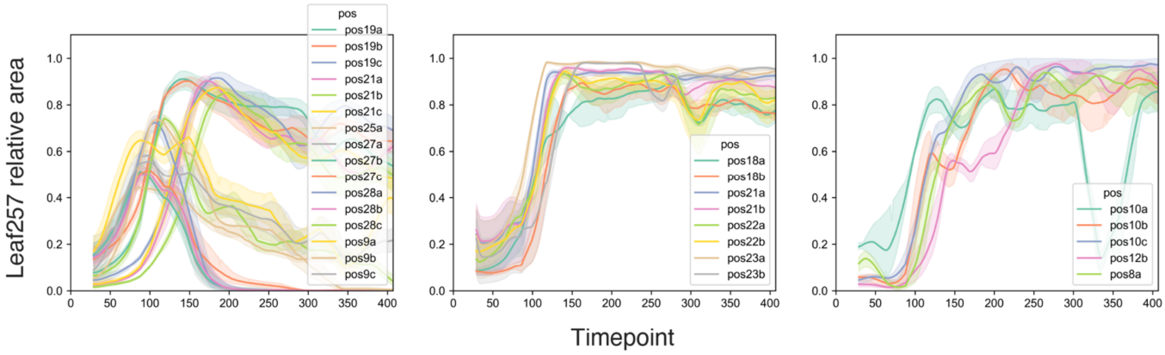


Supplementary Figure 14. Variation in estimated relative area occupied by *Sphingomonas* Leaf257 when paired with *Rhizobium* Leaf68 (left), *Microbacterium* Leaf203 (middle), and *Pseudorhodoferax* Leaf256 (right) across three rounds of training. The positions correspond to those referenced in Source Data 3a and 4b, the dark lines represent the mean relative area, and the shaded regions represent the standard deviation of relative area. The variation in relative area is no higher than 0.05 compared across three independently trained models per dataset.

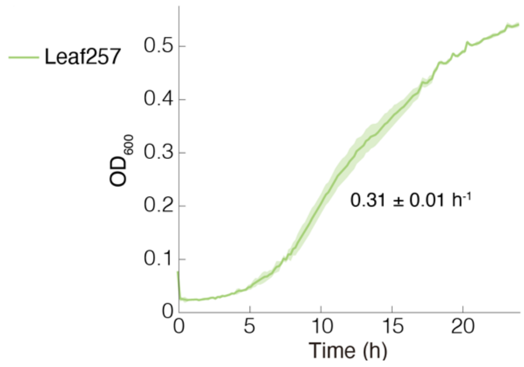


Supplementary Figure 15. Quantification of maximum growth rate of *Sphingomonas* Leaf257 in plate reader spectrophotometer (N = 3 biological replicates, minimum and maximum displayed in light green, mean growth displayed in dark green). $OD_{600}$ values displayed were corrected using a blank measurement. Strain was cultivated at 28˚C with orbital shaking on a minimal medium containing 1.32 g/L xylan as in the microfluidic experiments. The maximum growth rate was calculated by fitting the growth curve to a modified Gompertz growth model[2] using the Dashing Growth Curves tool[3] (Gompertz-tight). Quantity displayed denotes mean and standard deviation of maximum growth rates across the three replicates.

# References


1. Bai, Y. *et al.* Functional overlap of the *Arabidopsis* leaf and root microbiota. *Nature* **528**, 364–369 (2015).

2. Ghenu, A.-H., Marrec, L. & Bank, C. Challenges and pitfalls of inferring microbial growth rates from lab cultures. *Front. Ecol. Evol.* **11**, (2024).

3. Reiter, M. A. & Vorholt, J. A. Dashing Growth Curves: a web application for rapid and interactive analysis of microbial growth curves. *BMC Bioinformatics* **25**, 67 (2024).

4. Berg, S. *et al.* ilastik: interactive machine learning for (bio)image analysis. *Nat. Methods* **16**, 1226–1232 (2019).

5. Bechtold, B. Violin Plots for Matlab. github.com/bastibe/Violinplot-Matlab (2016).

6. Hintze, J. L. & Nelson, R. D. Violin Plots: A Box Plot-Density Trace Synergism. *Am. Stat.* **52**, 181 (1998).

7. Schäfer, M. *et al.* Metabolic interaction models recapitulate leaf microbiota ecology. *Science* **381**, eadf5121 (2023).

8. Ryback, B., Bortfeld-Miller, M. & Vorholt, J. A. Metabolic adaptation to vitamin auxotrophy by leaf-associated bacteria. *ISME J.* **16**, 2712–2724 (2022).
