# Supplementary material for: Metabolic feedbacks drive population dynamics and can lead to oscillations among leaf bacteria
Source: Nat Commun. 2026 May 29;17:6983. doi: 10.1038/s41467-026-73686-w (PMC13392072; doi:10.1038/s41467-026-73686-w)
Supplement: Supplementary file 2 — Descriptions of Additional Supplementary Files [file 41467_2026_73686_MOESM2_ESM.pdf]

Description of Additional Supplementary Files

Supplementary Data 1. Quantification of AUC values for NMR spectra of minimal media, of Sphingomonas Leaf257 effluents, and of effluents incubated with Rhizobium Leaf68, Microbacterium Leaf203, and Pseudorhodoferax Leaf265.

Supplementary Data 2. Quantification results of LC-MS measurements of vitamin standards, Sphingomonas Leaf257 effluents, and consumption profiles by interactor strains

Supplementary Video 1. Representative timelapse of Rhizobium Leaf68 in microfluidic chamber decoupled from Sphingomonas Leaf257, in a minimal medium with 1.32 g/L xylan as sole carbon source and no vitamins.

Supplementary Video 2. Representative timelapse of Rhizobium Leaf68 in microfluidic chamber coupled to Sphingomonas Leaf257, in a minimal medium with 1.32 g/L xylan as sole carbon source and no vitamins.

Supplementary Video 3. Representative timelapse of Microbacterium Leaf203 in microfluidic chamber decoupled from Sphingomonas Leaf257, in a minimal medium with 1.32 g/L xylan as sole carbon source and no vitamins.

Supplementary Video 4. Representative timelapse of Microbacterium Leaf203 in microfluidic chamber coupled to Sphingomonas Leaf257, in a minimal medium with 1.32 g/L xylan as sole carbon source and no vitamins.

Supplementary Video 5. Representative timelapse of Pseudorhodoferax Leaf265 in microfluidic chamber decoupled from Sphingomonas Leaf257, in a minimal medium with 1.32 g/L xylan as sole carbon source and no vitamins.

Supplementary Video 6. Representative timelapse of Pseudorhodoferax Leaf265 in microfluidic chamber coupled to Sphingomonas Leaf257, in a minimal medium with 1.32 g/L xylan as sole carbon source and no vitamins.

Supplementary Video 7. Representative timelapse of Microbacterium Leaf203 with Sphingomonas Leaf257-mCherry in mixed microfluidic chambers in a minimal medium with 1.32 g/L xylan as sole carbon source and no vitamins.

Supplementary Video 8. Representative timelapse of Pseudorhodoferax Leaf265 with Sphingomonas Leaf257-mCherry in mixed microfluidic chambers in a minimal medium with 1.32 g/L xylan as sole carbon source and no vitamins.

Supplementary Video 9. Representative timelapse of Rhizobium Leaf68 with Sphingomonas Leaf257-mCherry in mixed microfluidic chambers in a minimal medium with 1.32 g/L xylan as sole carbon source and no vitamins.

Supplementary Video 10. Representative timelapse of Rhizobium Leaf68 with Sphingomonas Leaf257-mCherry in mixed microfluidic chambers in a minimal medium with 1.32 g/L xylan, and 10 mM each of Dxylose, L-alanine, and acetate with no vitamins.

Supplementary Video 11. Representative timelapse of Rhizobium Leaf68 with Sphingomonas Leaf257-mCherry in mixed microfluidic chambers in a minimal medium with 1.32 g/L xylan and vitamins (pantothenate, niacin, biotin, and thiamine).

Supplementary Video 12. Representative timelapse of Rhizobium Leaf68 with Sphingomonas Leaf257-mCherry in mixed microfluidic chambers in a minimal medium with 1.32 g/L xylan, 10 mM each of D-xylose, L-alanine, and acetate, and vitamins (pantothenate, niacin, biotin, and thiamine).
