# Supplementary material for: Metabolic feedbacks drive population dynamics and can lead to oscillations among leaf bacteria
Source: Nat Commun. 2026 May 29;17:6983. doi: 10.1038/s41467-026-73686-w (PMC13392072; doi:10.1038/s41467-026-73686-w)
Supplement: Supplementary file 7 — Transparent Peer Review file [file 41467_2026_73686_MOESM7_ESM.pdf]

nature portfolio

Peer Review File

Metabolic feedbacks drive population dynamics and can lead to oscillations among leaf bacteria

Corresponding Author: Professor Julia Vorholt

This file contains all reviewer reports in order by version, followed by all author rebuttals in order by version.

Attachments originally included by the reviewers as part of their assessment can be found at the end of this file.

Version 0:

Reviewer comments:

Reviewer #1

(Remarks to the Author)
The manuscript by Pacheco et al. "Metabolic feedbacks drive population dynamics and can lead to oscillations among leaf bacteria" presents an insightful investigation into the metabolic interactions shaping microbiome assembly and function. By employing a microfluidic device for direct cell tracking and metabolite detection, the authors provide a detailed account of how plant polysaccharide degradation influences microbial interactions among leaf-associated bacteria. Their combination of experimental and computational approaches effectively identifies key metabolic mediators, revealing recipient-specific patterns of carbon substrate and cofactor complementation. Furthermore, the integration of metabolic modeling strengthens the study, allowing for systematic predictions of ecological outcomes, which are then validated experimentally. Overall, the study offers a valuable contribution to microbial ecology by elucidating how metabolic exchanges drive population dynamics and informing strategies for microbiome design. However there are areas which need improvements and clarifications. Here are my comments on some important points that have to be clarified or fixed before we can proceed and a positive action can be taken.

1. Line 375: The microfluidic device's design is based on previous architectures, but the rationale for selecting the specific chamber and channel dimensions is not provided. How do these dimensions optimize bacterial interaction studies?

2. Line 408: The Time-lapse Imaging of Bacterial Growth in Microfluidic Device was acquired by loading on the stage of a Nikon Ti2 microscope. I suggest the authors put additional details on exposure settings and image processing.

3. Line 416: The segmentation process is described using ilastik, but no validation is provided. Were manually counted bacterial cells compared with automated segmentation results to assess accuracy? Further, the ilastik model was trained on three fields of view from five randomly selected chambers. While this provides some robustness, it would be beneficial to report statistical variability across multiple training datasets.

4. Line 424: Were there any sterilization controls for effluent collection (e.g., plating aliquots on agar to check any contamination)? Also, It would be valuable to include some control experiments or conditions to demonstrate that the observed microbial interactions are indeed due to metabolic exchange and not due to physical confinement in the microchambers. For example, did the authors test for any possible artifacts due to the design of the device, such as microchamber crowding or diffusion limitations?

5. The process of cells being displaced from the microchambers and washed out through the channels is mentioned briefly. A more detailed explanation of this process would help clarify the procedure. For example, what mechanisms are in place to ensure that only displaced cells are washed out and not other particulates or metabolites? Could the washout step have affected the results in any way?

6.The selection of Sphingomonas Leaf257 and Rhizobium Leaf68 as the first pair of bacterial strains to study is an interesting choice, particularly given their known co-localization on Arabidopsis leaves. However, it would be helpful to clarify why these specific strains were chosen for this study beyond their co-localization. In addition to xylan-derived metabolic exchange, what are the other reasons related to the broader goal of understanding metabolic interactions? A brief explanation of their selection criteria would add context to the experiment.

7. The manuscript mentions that the co-colonization of Sphingomonas Leaf257 and Rhizobium Leaf68 led to a metabolic shift in Sphingomonas, particularly an increased expression of xylan degradation genes. It would be beneficial to provide more detail on gene expression on the nature and timing of this metabolic shift. Additionally, were any other metabolic shifts observed in Sphingomonas Leaf257, and how do these shifts compare with similar interactions in other bacterial species?

8. While the experiments are well-designed, it would be useful to include additional control experiments. Specifically, it would be helpful to demonstrate baseline growth conditions for each strain without the presence of xylan or the other interacting strain. This would establish a clearer context for the observed interactions and provide further support for the conclusion that xylan is driving the metabolic dependency.

9. The screen for metabolic dependencies across a broader set of leaf-associated bacteria is a valuable addition. However, it would be helpful to provide a more detailed analysis of why certain strains were unable to grow in the presence of xylan without Sphingomonas Leaf257. What specific metabolic pathways or capabilities might explain this dependency? Additionally, what role does xylan degradation play in shaping bacterial community structure on leaf surfaces in a natural conditions?

10. While the manuscript does a great job of explaining the metabolic interactions between strains, it would be beneficial to address whether similar interactions could be observed between other species, particularly within the broader context of leaf microbiomes. Are the metabolic interactions you've identified exclusive to these strains, or could they be generalized across other microbial communities?

Reviewer #2

(Remarks to the Author)
Pacheco et al. use a microfluidic approach to investigate interactions between leaf-associated bacterial isolates at the single-cell level. The microfluidic platform is based on published designs that trap bacteria within micron-sized chambers to allow (or not allow) cell-cell communication via the exchange of metabolites. The collection of effluent from the device is an especially nice idea. Please find below specific comments related to the microfluidics aspect of the work.

Major Comments:

- It is stated that the microfluidic device design is "based on" previous architectures (Line 375). Please can the authors provide the exact device design(s) (i.e., footprint) in the SI, and specify any changes made from the referenced designs (with justifications; it is not clear whether published designs are used without further modification).

- Lines 67-68: It is not immediately clear what is meant by "pores can either span the entire length of the interface between the opposing microchambers or be confined to the center". An overview of the complete device design (and different versions of the designs used) would help aid understanding of the nature of the experimental set up. Please also specify which design was used to collect the data for each figure, as several different designs appear to have been used.

- Can the authors comment on the starting numbers of bacteria in each of the microchambers. Is this controlled, i.e., 1 bacterial cell per chamber, and if not, how was this accounted for in the data analysis? For example, Fig. 1c quotes cell count on the y-axis, therefore presumably absolute cell numbers are reported (not normalised)?

- What is the maximum number of cells that fill a chamber (and are all of the bacterial isolates similar in size)? Bacteria can also be seen overflowing out of the chambers, for example in SI video 3. Could the authors comment on how this might influence the results?

- Can the authors comment on the rate of transfer of xylan into/out of the microchambers? Could this dilute or remove, for example, the metabolites exchanged between bacterial strains?

- Can the authors be 100% confident that bacteria do not cross the pores (have control experiments been performed)? For example, in SI video entitled "pos15a_203_257_segregated.avi" it appears there are bacteria emerging from pore region of the device (where there should be no bacteria). It has been reported that E. coli can change their morphology to morph through nanopores [Mannick et al, PNAS, 2009 106:14861; https://doi.org/10.1073/pnas.0907542106] – have the authors considered this?

- There are also devices with square pillars located in the microchannel (videos in the folder Supplementary Videos 3) – what are the purpose of these? Is this a different device design, with longer channels? This links to points raised above about providing further clarity on the exact device designs used, which is essential for ensuring reproducibility of data.

- Authors should also specify which version of the design was used for coculture of different strains with fluorescently tagged Sphingomonas Leaf257 (line 215). SI Video 3 (Leaf68Leaf257_Xylan+Vits.avi) has been cropped but in certain frames pores seem to be visible. If a device with pores was used for these experiments (always the same device?), can the authors comment on the effect nutrient diffusion through these pores might have on the population dynamics.

- Additionally, for the co-culture experiments, it is stated that the cells were mixed in a 1:1 ratio immediately before injection into the device. However, presumably this does not guarantee a 1:1 trapping ratio in the chambers; please can the authors comment on whether the starting ratios in the microchambers were guaranteed to be 1:1? If there are discrepancies in starting ratios this would presumably influence the reported results.

- Referencing of Supplementary Videos should be improved, as currently it is non-specific. For example, “Supplementary Video 1” refers to several movies contained within several sub-folders. Each video should have its own dedicated name and be reference individually to avoid confusion.

- In lines 131-132, the authors refer to a “radial growth pattern” where the “area of growth increased with the number of pores”, shown in Supplementary Figure 3. Representative images only are included in this figure, and the authors should quantify this radial growth pattern and correlate it with the number of pores in the chamber. An analysis of xylan rate of transfer across the pores could further consolidate this data.

- The first results section, entitled “A microfluidic platform for studying interactions between environmental microbes” do not detail any results (Lines 62-73). This is a repetition of the methods, where no new finding(s) nor data are presented, and should therefore be removed.

- Authors use should make use of the results section to explain the data more clearly and describe the results obtained. There are also instances, such as for Figure 2b, in which only part of a panel’s results are described and only several paragraphs later are the remaining data from the same graph described. This can be very confusing.

- Authors should reconsider the choice of data presented in Figure 1 since the second results paragraph focuses on results shown in SI Figures 1-3 and the main Figure 1 is not mentioned until line 112 (4th paragraph of this results section).

- The methods section for the microfluidic device design and fabrication could be improved; there is not enough detail provided here for the work to be reproduced. For example, what ratio of base:curing agent was employed in PDMS preparation, what was the exact plasma treatment used etc etc.

Minor comments:

- Please refer to data in Figures at the appropriate point in text; often SI figures are not referred to in order, SI figures 4, 5 and 6 are referred to in the opposite order.

- The microchamber depth is reported to be 1 um, which can quite deep for bacteria and result in loss of monolayer confinement due to the flexibility of PDMS (detrimental for imaging). Can the authors justify this height employed? Also, the quality of the TL videos in the SI is not optimal, where there is difficulty monitoring individual bacteria.

- In Supplementary Figure 2, the average line is represented by a thicker line of the same colour, but this can be hard to read in some of the panels. Can the authors use a darker colour to label the average line instead, for example.

- Line 238 (ref9) needs correcting.

Reviewer #3

(Remarks to the Author)
I co-reviewed this manuscript with one of the reviewers who provided the listed reports. This is part of the Nature Communications initiative to facilitate training in peer review and to provide appropriate recognition for Early Career Researchers who co-review manuscripts.

Reviewer #4

(Remarks to the Author)
In this study, the authors present a microfluidic device setup designed to track metabolites and, through extensive data analysis, gain insights into plant polysaccharide degradation by leaf-associated bacteria. The methodological approach is very much appreciated. What worried the reviewer the most are detailed aspects regarding the biochemical and biological relevance. Specifically, two main areas require further scrutiny: (1) the biological relevance of the study, particularly the use of extracted and uncharacterized xylan with potentially unrelated structures and the actual availability of xylan on leaf surfaces, and (2) the difficulty in connecting data to key statements due to the lack of essential NMR and MS data in the manuscript.

1) Biological and Biochemical Relevance of Xylan

The biological relevance of the xylan used in this study remains unclear. Xylan is presented in an oversimplified manner, whereas in reality, it is a highly complex polysaccharide with diverse chain lengths and substitution patterns (e.g., sidechains, acetylation). In plants, xylan is largely insoluble and forms an integral network with cellulose and lignin in secondary cell walls. This raises several concerns:

The study employs xylan from Roth. What is the chemical nature of these xylan molecules? Are they substituted? What is their average chain length? Two sources are mentioned (Beechwood and corncob from Roth)—which one was used?

How well do these xylan molecules represent those in Arabidopsis? If there are major structural differences between the model xylan and Arabidopsis xylan, how might this impact the study's observations and conclusions?

The authors mention a 10 mM xylan concentration. Given xylan's heterogeneous and polymeric nature, how was this concentration quantified?

The schematic illustrations are useful, but the tetramer representation does not accurately reflect xylan's structure. If oligomers were used, this should be clarified in the maintext to prevent misleading readers into assuming polysaccharides were utilized.

The presence of acetate is mentioned. Are the xylan molecules acetylated? In dicots, approximately half of the xylosyl residues are O-acetylated at C-2 or C-3, influencing xylan solubility and microbial degradation.

The absence of glucuronic acid discussion is notable, as it is a key xylan sidechain in Arabidopsis and hardwood species.

In line 370, the authors state, "Though the bioavailability of complex polymers like xylan may vary across the leaf surface…." This is problematic since xylan is not typically available on leaf surfaces—it is a major secondary cell wall component. This significantly impacts the discussion's key arguments and should be carefully reconsidered.

2) NMR and MS Data:

Solution NMR was used to identify key metabolic compounds necessary for xylan-dependent growth of Sphingomonas strains. Given its importance in tracking metabolite concentrations, the absence of NMR spectra in the main text or supplementary materials is concerning. It is essential to include spectra that support key claims, such as the presence of xylose in the effluent and significant metabolite abundance changes.

Additionally, the Methods section describes metabolite identification through manual comparison with standards, yet key spectra should be provided in the supplementary materials for transparency.

Data Availability: The original NMR and MS datasets should be deposited in public repositories to ensure accessibility.

In lines 164-165, the authors mention, "...their resource and vitamin uptake profiles, measured respectively by NMR and LC-MS, differed (Figure 2b)." However, the manuscript does not clarify how these molecular concentrations were determined, nor does Figure 2b or its caption explain the analytical methods. This lack of clarity makes it difficult to interpret the findings. Additionally, it is important to mention if there are potential discrepancies between NMR and LC-MS quantification.

Reviewer #5

(Remarks to the Author)
Summary

Pacheco et al. presents an interesting mixed computational/experimental manuscript that seeks to use metabolic models to explain community dynamics in a plant associated minimal co-culture. In principle the work approaches this question using the right system (defined media, reactor setup amenable to analysis, quantitative metabolomics, detailed metabolic models that are constrained using measurements, etc). Unfortunately, there are several shortcomings with the models and simulations that make it challenging to adjudicate the work. These should be addressed prior to evaluating the scientific merit of the work.

Major comments

Lines 234 - 243 motivate the use of genome-scale metabolic models in this work. The authors refined previously published models from their group, and made use of computational modeling that heavily relies on accurate, detailed metabolic capabilities of each strain as captured by the models. I downloaded the models from linked Github repo (commit 2435661), and attempted to test the quality of the models, since the simulation results depend on this. Due to time constraints, I only performed spot checks on metabolically important reactions and basic simulation "sanity tests" of the two models.

The results are summarized below:

1. The ATP synthase reaction ("R_ATPS4rpp") for Leaf68 only has two genes associated with it (to the best of my knowledge 8 proteins are required for the complex to be functional). On the other hand, Leaf257 had no genes associated with this reaction. Likewise, for both models the NADH dehydrogenase reaction ("R_NADH5", "R_NADH9", "R_NADH10") has multiple isozymes, but only one or two genes associated with each isozyme. In E. coli this complex requires 16 subunits to function.

Please comment on the biological realism of these reduced complexes.

Alternatively, if the models are not well curated it is recommended that the authors curate all reactions in both models to ensure that the gene reaction rules are correct. This is feasible to do given the number of authors on the paper. Curation of gene reaction rules is typically a required step when reconstructing a genome-scale metabolic model (otherwise why use such a model to begin with?), even if an automatic reconstruction tool has been used. Reactions without genetic evidence should ideally not form part of the final model, unless there is compelling experimental evidence for its existence. See https://www.nature.com/articles/nprot.2009.203 for guidelines on the construction of metabolic models. The removal of e.g. the ATP synthase in Leaf257 would dramatically alter the simulation results, emphasizing the importance of curating the models used in this work.

2. Next, I inspected the default bounds of reactions in the models. For the Leaf257 model the formate, H2O, and CO2 exchanges ("R_FORt2", "R_FORtex", "R_FORt", "R_H2Otex", "R_CO2tex") were artificially bounded. Please motivate why these bounds are necessary, how the actual bound values were calculated/estimated, and explain what the effect of removing them has on the simulation results.

3. For the other manually set bounds in the model, please show the calculations used to find these values. It was not clear how the authors went from the results in Figure 2.b (concentration measurements) to bound limits (flux limits). Since all the simulations were generated by varying these bounds, it seems important that they are better explained.

4. I had some problems connecting the actual genomes of the organisms with the accession numbers listed in the gene reaction rules. The manuscript references Schäfer et al., which links to BioProjects PRJNA297956 and PRJEB47672. I downloaded the predicted protein sequences for Leaf68 (https://www.ncbi.nlm.nih.gov/protein?term=txid1736231[Organism]) and Leaf257 (https://www.ncbi.nlm.nih.gov/protein?term=txid1736309[Organism]). There seems to be a mismatch between all of the model proteins in Leaf68 and the online resource, and some mismatches for Leaf257 (e.g. I could find "G_WP_056433836_1" but not "G_WP_007404217_1"). Was I looking at the wrong files, or are the models incorrect?

Lines 246 - 322 deal with the simulation results.

5. The authors made use of COMETS to simulate their models. This is a very sophisticated simulation tool, combining dFBA with, possibly spatial, differential equations. As such, I believe it is necessary that the authors mathematically describe (with equations) the exact system(s) they are integrating. Does it include spatial constraints, which equations are used to model uptake/secretion rates, diffusion, etc. Currently it is unclear, and the methods section does not provide enough detail. It is not enough to simply supply a settings file used to run COMETS, as this obscures the reasoning behind the simulation construction effort.

6. The authors describe that they constrained the growth rates of the organisms in their models. This is very unusual for constraint-based simulations, as typically a limiting nutrient uptake rate is fixed. Moreover, since they are using a dFBA-like tool to simulate their models, I would expect that it would be more natural to include xylan flux/concentration as the limiting constraint. This has the benefit that it can be directly measured in their setup. Why did the authors fix maximum growth rates? This might "bake in" the co-existence or oscillation results, and should be investigated.

7. Further, the authors measured the maximum growth rate of each organism in Figure S7, but they did not use the same media. This is not a fair comparison. Since Leaf68 is necessarily growth limited by vitamins (e.g. Fig S4), it would never reach the maximum growth rate calculated in their results, because the media is not representative of the culture conditions (Fig S2 seems far more appropriate, where are the growth rates calculated from these plots?). For this reason, I don't think currently used growth rate constraints are appropriate for this system. A Michaelis-Menten-like description of the growth rate vs. vitamin concentration seems more reasonable, especially given that dFBA actually models extracellular metabolite concentrations. Please justify.

8. The authors also find that vitamin auxotrophies are the primary determinant of community formation (lines 284 - 290 + 310 - 317). Is this not obvious from the outset, especially given access to the metabolic models which can be directly probed for vitamin, amino acid, etc. auxotrophies (cf. lines 118 - 121 which points towards a vague "xylan-based metabolic interactions")? Since Leaf68 is auxotrophic for vitamins, how can its growth not depend on the secretion of vitamins by Leaf257? A more important question is, why does Leaf257 secrete these costly products, how does it benefit?

9. The authors claim their simulations demonstrate oscillations. However, this is difficult to ascertain from Fig 3c (which itself is too compact) as the oscillations seem much more like a differential equation solver encountering numerical issues. Did the authors substantially (e.g. 2 orders of magnitude) reduce the step size to check that this is not just a numerical artifact? The period of oscillation also seems very short (or long, it is impossible to see in the figure), is this biologically realistic? How does it compare to the oscillations of Fig 3a? Additionally, Fig 3a (Leaf257-Leaf68) shows that Leaf257 has a high/oscillating abundance, but the oscillating simulations show a low abundance for Leaf257. Do the models incorrectly predict the abundances?

10. Fig 4a and 4b aim to demonstrate concordance between simulation results and experimental observations. In the trivial cases where the faster growing organism has all the required nutrients the data match (sort of), but for the case where only carbon sources are present the data do not match at all. For example, the time-scale is completely different (by an order of magnitude), the abundance time course profile is not similar (the initial conditions do not even match the experimental data),

and steady state is not reached for the experimental setup, making it hard to compare the results. Since the purpose of the work is to use models to investigate metabolic connection points in a co-culture, it stands to reason that the models should broadly match observations. Please justify the suitability of the simulations given this.

11. Do the initial abundances make a difference for the predicted dynamics?

12. The simulation output data supplied by the authors on Github is not presented in a format amenable to simple inspection. Could they please save their results as CSV files (e.g. biomassLog, fluxLog, etc.) that can be loaded into a tabular format? Additionally, could they please upload all the data used to make the figures (e.g. Fig 3c), and clearly explain how to reproduce their figures using the CSV files? The current state of the Github repo is not sufficient for this purpose.

Version 1:

Reviewer comments:

Reviewer #4

(Remarks to the Author)
The authors have appropriately addressed my comments. For those points that could not be fully resolved within the current experimental framework, they have provided clarifications. I would like to emphasize that xylan decorations and sidechains are critical determinants of xylan's chemicophysical properties and represent a key distinguishing feature among xylans from different plant species. The additional clarifications related to xylan sidechains included in the revised manuscript are therefore important.

Reviewer #5

(Remarks to the Author)
Please see the attached pdf for my comments.

Reviewer #6

(Remarks to the Author)
At the request of the editor, I have reviewed the authors' responses to Reviewer #1's comments and re-evaluated the manuscript.

First, the authors have addressed each of the points raised by Reviewer #1, particularly those related to experimental details. However, other aspects still require improvement. For instance, points six, nine, and ten concerning the rationale for strain selection and their ecological significance could be elaborated further in the Introduction.

While the authors adopt the phyllosphere as their research focus, the experiments only use leaf metabolites as a nutrient medium. This raises the question: Do healthy plant leaves genuinely secrete oligosaccharides? The scientific premise should clarify whether the study aims to explore microbial interactions in the healthy phyllosphere or the dynamics during leaf senescence and pathogen infection. This distinction is critical, as it determines whether the study aligns more with plant microbiome research on leaf health or ecological studies on litter decomposition. The authors should explicitly define and discuss this scientific context, as this point—previously highlighted by Reviewer #1—remains unaddressed in the current revision.

The experimental design, which relies solely on metabolites rather than living leaves, suggests that the authors may be leaning toward the latter scenario (litter decomposition). However, as Reviewer #1 noted, the study currently lacks a coherent scientific background to support this approach, creating a disconnect between the stated research framework and the actual experiments conducted.

I recommend relocating the background information presented in Result 1 to the Introduction. This would help streamline the Results section and improve its focus.

In Lines 144–146, the authors state: "Due to the likely overrepresentation of undegraded xylan in the medium stemming from the continuous flow inherent to the microfluidic device, we carried out a separate experiment in a batch culture system that further confirmed this degradation." However, the results related to the undegraded xylan are not presented. The authors should include these findings.

Additionally, Sections 1, 2, and 4 of the Results could be condensed and summarized more effectively to enhance readability.

Reviewer #7

(Remarks to the Author)

The authors responded appropriately to the comments raised.

Version 2:

Reviewer comments:

Reviewer #5

(Remarks to the Author)
The authors have appropriately addressed my concerns.

Open Access This Peer Review File is licensed under a Creative Commons Attribution 4.0 International License, which permits use, sharing, adaptation, distribution and reproduction in any medium or format, as long as you give appropriate credit to the original author(s) and the source, provide a link to the Creative Commons license, and indicate if changes were made.
In cases where reviewers are anonymous, credit should be given to 'Anonymous Referee' and the source.
The images or other third party material in this Peer Review File are included in the article's Creative Commons license, unless indicated otherwise in a credit line to the material. If material is not included in the article's Creative Commons license and your intended use is not permitted by statutory regulation or exceeds the permitted use, you will need to obtain permission directly from the copyright holder.
To view a copy of this license, visit https://creativecommons.org/licenses/by/4.0/

We sincerely thank the reviewers for their careful reading of our manuscript and their helpful and constructive comments. We have addressed these in the revised manuscript file (please note that the line numbers refer to the manuscript file with tracked changes), and have provided a point-by-point response to each below:

Reviewer: 1

The manuscript by Pacheco et al. "Metabolic feedbacks drive population dynamics and can lead to oscillations among leaf bacteria" presents an insightful investigation into the metabolic interactions shaping microbiome assembly and function. By employing a microfluidic device for direct cell tracking and metabolite detection, the authors provide a detailed account of how plant polysaccharide degradation influences microbial interactions among leaf-associated bacteria. Their combination of experimental and computational approaches effectively identifies key metabolic mediators, revealing recipient-specific patterns of carbon substrate and cofactor complementation. Furthermore, the integration of metabolic modeling strengthens the study, allowing for systematic predictions of ecological outcomes, which are then validated experimentally. Overall, the study offers a valuable contribution to microbial ecology by elucidating how metabolic exchanges drive population dynamics and informing strategies for microbiome design. However there are areas which need improvements and clarifications. Here are my comments on some important points that have to be clarified or fixed before we can proceed and a positive action can be taken.

We thank the reviewer for the positive assessment of our work and for the valuable suggestions on how to improve its clarity.

1. Line 375: The microfluidic device's design is based on previous architectures, but the rationale for selecting the specific chamber and channel dimensions is not provided. How do these dimensions optimize bacterial interaction studies?

   Thank you for pointing out this area for clarification. To improve clarity regarding the specifics of the microfluidics system, we have revised the Methods section (Line 388) and added a new Supplementary Figure (Supplementary Figure 2), describing the chosen microfluidic geometries in greater detail in relation to the size of the bacteria studied (see figure caption). Briefly, a chamber height of 1 µm was chosen to enable entry and trapping of average-sized bacteria, while the chamber width and length of 40 or 50 µm was determined to be large enough to capture a small population of cells while minimizing metabolite gradients that would introduce significant non-uniformities, based on previous experience and published work (Dal Co et al.*, 2019, DOI: 10.1098/rstb.2019.0080; Dal Co* et al.*, 2019, DOI: 10.1098/rsif.2019.0182).*

2. Line 408: The Time-lapse Imaging of Bacterial Growth in Microfluidic Device was acquired by loading on the stage of a Nikon Ti2 microscope. I suggest the authors put additional details on exposure settings and image processing.

   We agree that it will be useful to other users to have this information and have now expanded the Methods section by adding technical details on exposure and image processing for the microscopy (Line 448).

3. Line 416: The segmentation process is described using ilastik, but no validation is provided. Were manually counted bacterial cells compared with automated segmentation results to assess accuracy? Further, the ilastik model was trained on three fields of view from five randomly selected chambers. While this provides some robustness, it would be beneficial to report statistical variability across multiple training datasets.

   *Thank you for this comment. We first clarify that these data are based not on counts of individual cells, but on the quantification of the areas covered by the mCherry-*Sphingomonas *Leaf257 and*

by the untagged interactor populations during co-culture in single chambers (Figure 3a), using automatic segmentation through ilastik. To the reviewer's point, in order to quantify the variability across training datasets, we carried out three rounds of training on three different datasets per condition, shown in the figure below, now included as Supplementary Figure 13:

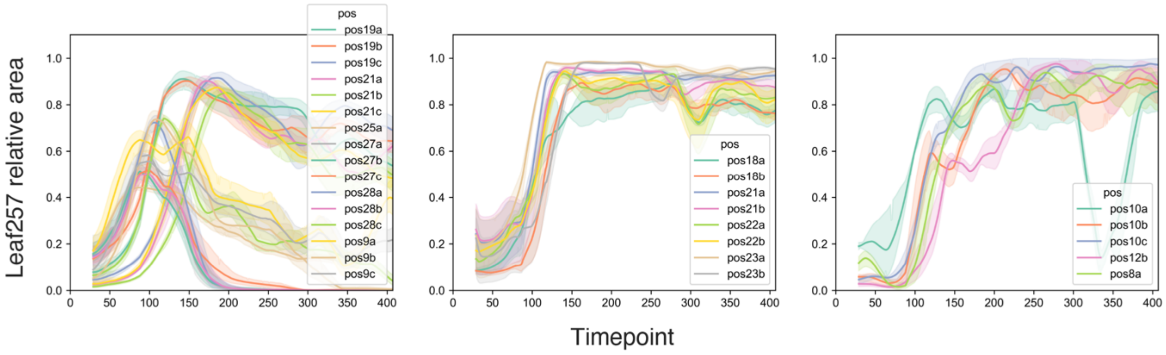

As in Figures 3a and 4b, we quantify the relative area occupied by Sphingomonas *Leaf257. Here, we display the variation in this quantity when Leaf257 is paired with* Rhizobium *Leaf68 (left),* Microbacterium *Leaf203 (middle), and* Pseudorhodoferax *Leaf256 (right) across three rounds of training. The positions correspond to those referenced in Source Data 3a and 4b, the dark lines represent the mean relative area, and the shaded regions represent the standard deviation of relative area. The variation in relative area is no higher than 0.05 compared across three independently trained models per dataset, allowing us to assess the robustness of this approach.*

4. Line 424: Were there any sterilization controls for effluent collection (e.g., plating aliquots on agar to check any contamination)? Also, It would be valuable to include some control experiments or conditions to demonstrate that the observed microbial interactions are indeed due to metabolic exchange and not due to physical confinement in the microchambers. For example, did the authors test for any possible artifacts due to the design of the device, such as microchamber crowding or diffusion limitations?

   The effluents were continuously filter-sterilized upon collection from the microfluidic device (Line 471). These were either immediately frozen at -20˚C for analysis with NMR and LC-MS, or inoculated with each interactor strain. These cultivation experiments, for which the growth of each strain is reported in Supplementary Figure 7, were carried out with uninoculated controls (i.e., filtered Leaf257 effluent), in which we measured no residual bacterial growth (OD_{600} of 0.0003 ± 0.0005 (mean ± s.d. across 3 replicates). We have added this information to the caption of Supplementary Figure 7.

   *As for the nature of the interactions, we agree that it is crucial to demonstrate that they are due to metabolic exchange. We would like to point the reviewer to the experiments shown in Figure 2, where we supplied recipient strains with supernatant of the xylan-degrading strain (*Sphingomonas *Leaf257). These experiments rule out physical confinement between the interacting strains as a potential confounding factor and demonstrate that growth of the recipients is enabled by metabolites derived from xylan degradation by Leaf257.*

5. The process of cells being displaced from the microchambers and washed out through the channels is mentioned briefly. A more detailed explanation of this process would help clarify the procedure. For example, what mechanisms are in place to ensure that only displaced cells are washed out and not other particulates or metabolites? Could the washout step have affected the results in any way?

   Thank you for pointing out this point for clarification. Each chamber within the microfluidic device acts in a similar way to a chemostat, in which fresh medium (e.g., containing xylan) is provided via the flanking channels and leads to a steady state of metabolites within the chambers. The availability of the medium components allows the bacterial populations within the chambers

to grow, and they are continuously washed out via the flanking channel once the cells fill the chamber. As such, the continuous overflow of cells from the chambers into the flanking channels is due to the inherent growth of the bacterial populations and is part of the normal functioning of such microdevices (see, e.g., Dal Co et al.*, 2020, DOI: 10.1038/s41559-019-1080-2; D'Souza* et al.*, 2021, DOI: 10.1038/s41396-021-00953-7). Importantly, it is not a separate step but rather produces a steady state in which a growing population of cells can fill and occupy a chamber without systematically biasing the observed interaction patterns. We have rephrased our description of the microfluidic device (Line 80), clarified this point in the Methods (Line 396), and added an additional figure (Supplementary Figure 2) that provides a more detailed description of the device's geometry and operation.*

6. The selection of Sphingomonas Leaf257 and Rhizobium Leaf68 as the first pair of bacterial strains to study is an interesting choice, particularly given their known co-localization on Arabidopsis leaves. However, it would be helpful to clarify why these specific strains were chosen for this study beyond their co-localization. In addition to xylan-derived metabolic exchange, what are the other reasons related to the broader goal of understanding metabolic interactions? A brief explanation of their selection criteria would add context to the experiment.

   We have added details to the Methods – "Selection and pre-cultivation of phyllosphere strains" – to clarify our choice of isolates (Line 410). We have also expanded our discussion of the ecological role of Sphingomonas *Leaf257 at the end of the manuscript (Line 369).*

7. The manuscript mentions that the co-colonization of Sphingomonas Leaf257 and Rhizobium Leaf68 led to a metabolic shift in Sphingomonas, particularly an increased expression of xylan degradation genes. It would be beneficial to provide more detail on gene expression on the nature and timing of this metabolic shift. Additionally, were any other metabolic shifts observed in Sphingomonas Leaf257, and how do these shifts compare with similar interactions in other bacterial species?

   This metabolic shift, reported in Hemmerle et al.*, 2022 (DOI: 10.1038/s41467-022-30469-3), involved the significant regulation of 55 proteins by* Sphingomonas *Leaf257 in response to co-colonization with* Rhizobium *Leaf68* in planta. *In addition to induction of polysaccharide degrading enzymes (e.g., a xylosidase/arabinosidase (ASF14_10230 5.2-fold), a xylan 1,4-beta-xylosidase (ASF14_16825, 5.2-fold), a glucan 1,4-beta-glucosidase (ASF14_10240, 4.2-fold), and another xylanase (ASF14_16480, 3.1-fold)), these included:*

   - *Induction of dipeptidyl peptidase-4, which cleaves X-proline dipeptides from the N-terminus of polypeptides (ASF14_01870, 6.4-fold), and is relevant for cleaving glycoproteins in plant cell walls (Kavi Kishor,* et al.*, 2015, DOI: 10.3389/fpls.2015.00544).*
   - *Induction of 6-phosphogluconolactonase (ASF14_10560, 2.3-fold), relevant for metabolizing glucuronic acid which forms part of xylan sidechains in* Arabidopsis *(Mortimer* et al.*, 2015, DOI: 10.1111/tpj.12898).*

   We nonetheless note that, as this shift has already been described in Hemmerle et al.*, we sought to limit our discussion of it in this work for the sake of conciseness. However, we recognize that it could be misinterpreted as a new finding in this study since it is mentioned at the start of the Results section. To clarify this, we have modified the wording in the Results (Line 69).*

8. While the experiments are well-designed, it would be useful to include additional control experiments. Specifically, it would be helpful to demonstrate baseline growth conditions for each strain without the presence of xylan or the other interacting strain. This would establish a clearer context for the observed interactions and provide further support for the conclusion that xylan is driving the metabolic dependency.

Thank you for this assessment of our experiments and for pointing out this area for improvement. We have now included results from a control experiment performed in a plate reader spectrophotometer, where we cultivated the interactor strains in a minimal medium containing either no carbon, xylan plus the carbon sources and vitamins released by Sphingomonas *Leaf257, and a standard rich medium for comparison (Supplementary Figure 10). This experiment confirmed that none of the strains grew without carbon, or supplementation of resources by Leaf257. These results are additionally now referenced in the text (Line 201).*

9. The screen for metabolic dependencies across a broader set of leaf-associated bacteria is a valuable addition. However, it would be helpful to provide a more detailed analysis of why certain strains were unable to grow in the presence of xylan without Sphingomonas Leaf257. What specific metabolic pathways or capabilities might explain this dependency? Additionally, what role does xylan degradation play in shaping bacterial community structure on leaf surfaces in a natural conditions?

*Of the strains that we screened, only the three that we analysed in more detail (*Rhizobium *Leaf68,* Microbacterium *Leaf203, and* Pseudorhodoferax *Leaf265) were unable to grow in the minimal medium with xylan as a sole carbon source. As provision of dethiobiotin, niacin, and pantothenate by Leaf257 enabled Leaf68 to utilize the xylan in the medium, we looked deeper into the vitamin requirements of the other two strains. This analysis, reported in Supplementary Figure 9, revealed that both Leaf203 and Leaf265 are auxotrophs for pantothenate and – possibly – for biotin as well. However, neither strain utilized the xylan in the medium once provided with these vitamins (Figure 2b). This result, as the reviewer notes, likely points to a more complex molecular basis for their inability to grow on the minimal medium with xylan.*

Our analysis of the genomes of the strain collection (Supplementary Figure 1, see inset below for details on the strains in this study) showed that Leaf265 lacks a glycosyl hydrolase. This gene is present in all other strains in our study that can grow on xylan as the sole carbon source, except for Aeromicrobium *Leaf289. As this latter strain was nonetheless able to grow on the minimal medium (Figure 1c), we cannot conclusively attribute the ability to grow on xylan to the presence or absence of this or any other single gene.*

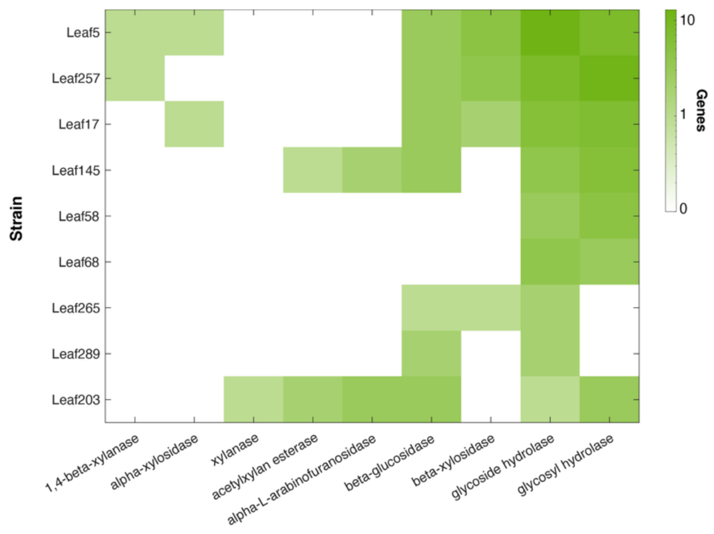

Despite this inconsistency, our results do shed light on the importance of a strain's ability to both degrade plant polysaccharides and provide vitamins to other community members. As such – and in following the reviewer's suggestion – we have expanded our discussion of the ecological role of xylan and of strains like Sphingomonas *Leaf257 in the Discussion (Lines 339 and 369).*

10. While the manuscript does a great job of explaining the metabolic interactions between strains, it would be beneficial to address whether similar interactions could be observed between other species, particularly within the broader context of leaf microbiomes. Are the metabolic interactions you've identified exclusive to these strains, or could they be generalized across other microbial communities?

    Thank you for this point. We have modified the manuscript to better link the results from our experiments to a broader ecological context. Beyond clarifying the selection criteria for the strains (Line 410), we have also clarified the ecological potential of other xylan degraders (Line 110). As mentioned above, we have expanded the Discussion to provide a clearer link to our analysis of strains with genes related to plant polysaccharide degradation (Supplementary Figure 1) and their connection to vitamin production.

Reviewer: 2

Pacheco et al. use a microfluidic approach to investigate interactions between leaf-associated bacterial isolates at the single-cell level. The microfluidic platform is based on published designs that trap bacteria within micron-sized chambers to allow (or not allow) cell-cell communication via the exchange of metabolites. The collection of effluent from the device is an especially nice idea. Please find below specific comments related to the microfluidics aspect of the work.

We thank the reviewer for the thoughtful comments and suggestions on how to improve our manuscript.

Major comments:

1. It is stated that the microfluidic device design is "based on" previous architectures (Line 375). Please can the authors provide the exact device design(s) (i.e., footprint) in the SI, and specify any changes made from the referenced designs (with justifications; it is not clear whether published designs are used without further modification).

   We have expanded the Methods section with more specifics on the chosen microfluidic design (Line 388) and have added a new Supplementary Figure that clarifies its geometry (Supplementary Figure 2). Briefly, a chamber height of 1 µm was chosen to enable entry and trapping of average-sized bacteria, while the chamber width and length of 40 or 50 µm was determined to be large enough to capture a small population of cells while minimizing metabolite gradients that would introduce significant non-uniformities, based on previous experience and published work (Dal Co et al.*, 2019, DOI: 10.1098/rstb.2019.0080; Dal Co* et al.*, 2019, DOI: 10.1098/rsif.2019.0182).*

2. Lines 67-68: It is not immediately clear what is meant by "pores can either span the entire length of the interface between the opposing microchambers or be confined to the center". An overview of the complete device design (and different versions of the designs used) would help aid understanding of the nature of the experimental set up. Please also specify which design was used to collect the data for each figure, as several different designs appear to have been used.

   We thank the reviewer for pointing this out. We have now provided a detailed description of the designs in Supplementary Figure 2, whose caption details which specific geometries were used for each experiment. We also direct the reviewer to the results in Supplementary Figure 4, which describe experiments conducted in chambers with narrower interfaces (i.e., in which only the central part of the interface between the two opposing microchambers has pores, as opposed to the entire interface).

3. Can the authors comment on the starting numbers of bacteria in each of the microchambers. Is this controlled, i.e., 1 bacterial cell per chamber, and if not, how was this accounted for in the data analysis? For example, Fig. 1c quotes cell count on the y-axis, therefore presumably absolute cell numbers are reported (not normalised)?

   Although the initial number of bacteria per microchamber was not strictly controlled, it remained within a similar range (± 30 cells (s.d.) on average across all conditions in Figure 1c and in Supplementary Figure 3) across replicate chambers. The cell numbers reported in Figure 1c and in Supplementary Figure 3 are indeed absolute, without correction, providing an indication of the initial cell number and its variability in conditions where no growth of the recipient is observed.

4. What is the maximum number of cells that fill a chamber (and are all of the bacterial isolates similar in size)? Bacteria can also be seen overflowing out of the chambers, for example in SI video 3. Could the authors comment on how this might influence the results?

Thank you for this important point. Continuous overflow of cells from the chambers into the channel is indeed due to growth of the populations and is part of the normal functioning of such microdevices (see, e.g., Dal Co et al.*, 2020, DOI: 10.1038/s41559-019-1080-2; D'Souza* et al.*, 2021, DOI: 10.1038/s41396-021-00953-7). Notably, it is not a separate step but rather a result of continuous influx of medium into the chambers. This dynamic results in a steady state – akin to a chemostat – in which a growing population of cells can stably fill and occupy a chamber and does not systematically bias the observed interaction patterns. To clarify this point, we have rephrased our description of the microfluidic device (Line 80), added more detail on the device functioning to the Methods (Line 396), and added an additional figure (Supplementary Figure 2) that provides a more detailed description of the device's geometry and operation.*

The natural isolates of the At-*LSPHERE collection do vary in size, leading to potential differences in the total number of cells per chamber (Supplementary Figure 3). However, we do not expect this to impact the interpretation of our results, as potential interactor strains were all screened against the same strain –* Sphingomonas *Leaf257. This strain was kept constant across all experiments, for which we estimate that ~200–300 cells typically accumulate in a chamber at steady state.*

5. Can the authors comment on the rate of transfer of xylan into/out of the microchambers? Could this dilute or remove, for example, the metabolites exchanged between bacterial strains?

   The constant flow rate in the flanking channel acts as a boundary condition that provides a constant concentration of xylan at the entrance to the microchambers. Similarly, this boundary condition acts as a sink for metabolites produced in the chamber. As also discussed in the previous comment, this constant flow of fresh medium enables the emergence of a steady state for the populations within the chambers. We have clarified this point in the Methods (Line 396), as well as added an additional figure (Supplementary Figure 2) with more details on the device design.

6. Can the authors be 100% confident that bacteria do not cross the pores (have control experiments been performed)? For example, in SI video entitled "pos15a_203_257_segregated.avi" it appears there are bacteria emerging from pore region of the device (where there should be no bacteria). It has been reported that E. coli can change their morphology to morph through nanopores [Mannick et al, PNAS, 2009 106:14861; https://doi.org/10.1073/pnas.0907542106] – have the authors considered this?

   We agree with the reviewer that this is an important aspect. Indeed, on some occasions (likely due to a weaker bonding of the device to the glass slide) cells were able to penetrate the connecting region and invade the adjacent chambers. We now clarify that we considered these events a device failure and discarded all data related to them (Line 453). Furthermore, we have visually re-checked all the timelapses for the data included in the manuscript, and in no case was such a cross-over event observed.

   We also clarify that the video mentioned by the reviewer does not represent such an event. While bacteria may seem to "emerge" from the pore region this is likely due to their vicinity to the pores, as no bacteria can be seen inside any of the pores and, furthermore, the different morphologies of Leaf203 and Leaf257 appear well-segregated in the chambers without intrusion of one strain in the opposite chamber.

7. There are also devices with square pillars located in the microchannel (videos in the folder Supplementary Videos 3) – what are the purpose of these? Is this a different device design, with longer channels? This links to points raised above about providing further clarity on the exact device designs used, which is essential for ensuring reproducibility of data.

   Following this and other suggestions, we now revised the manuscript adding a description of the designs in Supplementary Figure 2. With respect to the specific point raised here, we did include

square pillars in every other chamber as a means of avoiding sagging of the chamber during bonding procedures. The small surface area occupied by the pillars did not alter the growth of the populations (Supplementary Figure 3).

8. Authors should also specify which version of the design was used for coculture of different strains with fluorescently tagged Sphingomonas Leaf257 (line 215). SI Video 3 (Leaf68Leaf257_Xylan+Vits.avi) has been cropped but in certain frames pores seem to be visible. If a device with pores was used for these experiments (always the same device?), can the authors comment on the effect nutrient diffusion through these pores might have on the population dynamics.

   We have now provided details on which designs correspond to each experiment in Supplementary Figure 2. Regarding the experiments involving co-culture dynamics of interacting strains, we collected data from chambers with 2 or 3 pores for consistency, however, we did not observe significant changes in the outcomes of population dynamics in nearby chambers with a different number of pores. The presence of pores at the back of the chambers also has the beneficial effect of providing nutrients from the opposite side of the chamber, thus providing a more uniform nutrient field.

9. Additionally, for the co-culture experiments, it is stated that the cells were mixed in a 1:1 ratio immediately before injection into the device. However, presumably this does not guarantee a 1:1 trapping ratio in the chambers; please can the authors comment on whether the starting ratios in the microchambers were guaranteed to be 1:1? If there are discrepancies in starting ratios this would presumably influence the reported results.

   Thank you for this insightful comment. Although we mixed the strains at a 1:1 ratio before injection, the reviewer is correct that this does not guarantee a 1:1 colonization of each individual chamber. For this reason, we rely on multiple replicate chambers to assess any population dynamics (Figure 3a, Figure 4b).

   Nonetheless, it remains important to assess whether different starting abundances could have an effect on the ensuing dynamics. To determine this, we carried out an additional set of metabolic modelling simulations, in which we varied the initial abundances of Sphingomonas *Leaf257 and* Rhizobium *Leaf68 in a medium containing xylan as sole carbon source with no vitamins. The results of these simulations, described in Supplementary Figure 11, highlight that an outcome of coexistence – following population abundance fluctuations – is largely robust to the initial population sizes. This is with the exception of very small initial abundances of Leaf68, in which the population is unable to establish. We have now added a discussion of this result to the text (Line 296).*

10. Referencing of Supplementary Videos should be improved, as currently it is non-specific. For example, "Supplementary Video 1" refers to several movies contained within several sub-folders. Each video should have its own dedicated name and be reference individually to avoid confusion.

    We appreciate this point and have now labelled each Supplementary Video with its dedicated name, referencing them accordingly throughout the text.

11. In lines 131-132, the authors refer to a "radial growth pattern" where the "area of growth increased with the number of pores", shown in Supplementary Figure 3. Representative images only are included in this figure, and the authors should quantify this radial growth pattern and correlate it with the number of pores in the chamber. An analysis of xylan rate of transfer across the pores could further consolidate this data.

    We agree with the reviewer that a more quantitative approach would strengthen this point. We have therefore estimated the radius of active growth with respect to the number of pores via an optical flow image analysis, which we now report in Supplementary Figure 4.

12. The first results section, entitled “A microfluidic platform for studying interactions between environmental microbes” do not detail any results (Lines 62-73). This is a repetition of the methods, where no new finding(s) nor data are presented, and should therefore be removed.

    We have removed this section and have added a substantially abridged description of the device to the next (now first) section of the Results (Line 80).

13. Authors use should make use of the results section to explain the data more clearly and describe the results obtained. There are also instances, such as for Figure 2b, in which only part of a panel’s results are described and only several paragraphs later are the remaining data from the same graph described. This can be very confusing.

    We thank the reviewer for this comment and have sought to balance the clarity of the narrative with the data presented in each figure. To do this, we have (1) abridged the first section of the Results to prioritize description of the chosen strains and emergent interactions, (2) ensure that the figures and supplementary materials are referenced in the correct order, and (3) labelled and referenced each Supplementary Video individually.

    To clarify our observation of population fluctuations between Sphingomonas *Leaf257 and* Rhizobium *Leaf68, we have now carried out an additional analysis that quantifies the amplitude changes (measured by peak-to-trough difference of relative chamber area) experienced by the Leaf257 population. This is now included as Figure 3c.*

14. Authors should reconsider the choice of data presented in Figure 1 since the second results paragraph focuses on results shown in SI Figures 1-3 and the main Figure 1 is not mentioned until line 112 (4th paragraph of this results section).

    We agree that Figure 1 should have been cited earlier, which we now do in the section that describes the dependence of the growth of Leaf68 on Leaf257 (Line 82).

15. The methods section for the microfluidic device design and fabrication could be improved; there is not enough detail provided here for the work to be reproduced. For example, what ratio of base:curing agent was employed in PDMS preparation, what was the exact plasma treatment used etc etc.

    Thank you. We have now included additional details in the Methods section on PDMS curing, preparation and bonding (Line 402).

Minor comments:

16. Please refer to data in Figures at the appropriate point in text; often SI figures are not referred to in order, SI figures 4, 5 and 6 are referred to in the opposite order.

    Thank you for pointing this out. The order of the SI figures has been corrected.

17. The microchamber depth is reported to be 1 um, which can quite deep for bacteria and result in loss of monolayer confinement due to the flexibility of PDMS (detrimental for imaging). Can the authors justify this height employed? Also, the quality of the TL videos in the SI is not optimal, where there is difficulty monitoring individual bacteria.

    We agree with this consideration and have provided further detail and justification for the design specifics chosen. When carrying out a screening of several environmental strains, it is unlikely to find a single height value that matches the size of all strains. Indeed, as the strains we screened vary in size, a given chamber height can lead either to one strain not being able to populate the chambers (too thin) and another forming packed aggregates of cells after filling the chamber (too

high). We thus opted for a chamber height that allowed most strains to enter and populate the chambers – even though this resulted in the loss of single-cell resolution for some strains. As such, our work beyond the interaction screen relied more on population-level measurements (e.g., relative area of Sphingomonas *Leaf257 in mixed chambers) rather than single-cell level quantifications. We included part of this explanation in Supplementary Figure 2 where we describe the microfluidic device geometries in further detail.*

18. In Supplementary Figure 2, the average line is represented by a thicker line of the same colour, but this can be hard to read in some of the panels. Can the authors use a darker colour to label the average line instead, for example.

    We have modified the figure accordingly (now Supplementary Figure 3).

19. Line 238 (ref9) needs correcting.

    We had included 'ref' in parentheses to avoid confusion in the draft, as the reference immediately followed the numerical characters in 'Leaf68.' It has now been modified (Line 238).

Reviewer: 3

I co-reviewed this manuscript with one of the reviewers who provided the listed reports. This is part of the Nature Communications initiative to facilitate training in peer review and to provide appropriate recognition for Early Career Researchers who co-review manuscripts.

We thank the reviewer for the attention paid to our manuscript and for contributing to the helpful comments.

Reviewer: 4

In this study, the authors present a microfluidic device setup designed to track metabolites and, through extensive data analysis, gain insights into plant polysaccharide degradation by leaf-associated bacteria. The methodological approach is very much appreciated. What worried the reviewer the most are detailed aspects regarding the biochemical and biological relevance. Specifically, two main areas require further scrutiny: (1) the biological relevance of the study, particularly the use of extracted and uncharacterized xylan with potentially unrelated structures and the actual availability of xylan on leaf surfaces, and (2) the difficulty in connecting data to key statements due to the lack of essential NMR and MS data in the manuscript.

We thank their reviewer for their detailed reading of our manuscript and for pointing out important areas for clarification.

1. Biological and Biochemical Relevance of Xylan:

   The biological relevance of the xylan used in this study remains unclear. Xylan is presented in an oversimplified manner, whereas in reality, it is a highly complex polysaccharide with diverse chain lengths and substitution patterns (e.g., sidechains, acetylation). In plants, xylan is largely insoluble and forms an integral network with cellulose and lignin in secondary cell walls. This raises several concerns:

   a. The study employs xylan from Roth. What is the chemical nature of these xylan molecules? Are they substituted? What is their average chain length? Two sources are mentioned (Beechwood and corncob from Roth)—which one was used?

      We employ xylan derived from corncob (Roth), which is composed of ≥95 % xylooligosaccharides ($(C_5H_8O_4)_n$), each with a molecular weight of 132 g/mol. This characterization is based both on the manufacturer's website (Catalogue no. 8659) and the certificate of analysis for the specific product we used (Lot no. 089281125), which we have now clarified in the Methods (Line 432). As the manufacturer's definition of oligosaccharides can span from 3 to 9 monomeric subunits, we relied on the supplied molecular weight to calculate an equivalent concentration of xylose monomers (i.e., our medium contained 1.32 g/L xylan). This is congruent with the method we used to quantify the concentration of xylan via NMR, which relies on a portion of the spectrum corresponding to one of its constituent xylose monomers.

      However, we recognize that the wording '10 mM xylan' that we have used in the manuscript may be confusing. We have thus also changed how we describe the medium in the revised manuscript, either stating 1.32 g/L xylan or clarifying that it corresponds to 10 mM of its constituent monomers.

   b. How well do these xylan molecules represent those in Arabidopsis? If there are major structural differences between the model xylan and Arabidopsis xylan, how might this impact the study's observations and conclusions?

      Thank you – we have clarified the text on key aspects of the xylan we used in the manuscript (Line 432). Though the xylan we employed is not the native molecule found in Arabidopsis*, its main subunits (i.e., β-1,4-linked-D-xylopyranosyl residues) are by definition the same as those reported for* Arabidopsis *(e.g., Mortimer* et al.*, 2015, DOI: 10.1111/tpj.12898). With this key similarity in mind, we decided to proceed with this xylan as it not only allowed us to study the degradation of the molecule into its constituent monomers by Leaf257, but was also a commercially available source that helped ensure reproducibility in our study. We further note that the representativeness of this same source of xylan had been previously validated in the context of an interaction between* Sphingomonas *Leaf257 and* Rhizobium *Leaf68 (Hemmerle* et al.*, 2022, DOI: 10.1038/s41467-022-30469-3).*

Nonetheless, we recognize that the corncob xylan oligomers that we used can vary from those found in Arabidopsis *both in their length and – as the reviewer mentions in points 1e and 1f below – in their sidechains. For example, while our NMR measurements of the xylan point to it being acetylated, we did not detect any release of glucuronic acid as part of its degradation by Leaf257, suggesting that it is missing this sidechain found in* Arabidopsis*. Given the importance of acetate in the interaction with* Pseudorhodoferax *Leaf265 (Figure 2), we cannot exclude that release of glucuronic acid may also play an ecological role for strains interacting with Leaf257 on living plants. We added a note in reference to these considerations in the Discussion (Line 339).*

c. The authors mention a 10 mM xylan concentration. Given xylan's heterogeneous and polymeric nature, how was this concentration quantified?

We have clarified the amount of xylan used in the medium as part of our reply to point 1a above.

d. The schematic illustrations are useful, but the tetramer representation does not accurately reflect xylan's structure. If oligomers were used, this should be clarified in the maintext to prevent misleading readers into assuming polysaccharides were utilized.

We have changed all mentions of 'polysaccharide' to 'oligosaccharide' and 'polymer' to 'oligomer' throughout the manuscript where applicable to improve clarity. Along with replacing the visual icon representations of alanine and acetate in Figure 2c, we have also added a note to the caption clarifying that the xylan and xylose molecules are simplified.

e. The presence of acetate is mentioned. Are the xylan molecules acetylated? In dicots, approximately half of the xylosyl residues are O-acetylated at C-2 or C-3, influencing xylan solubility and microbial degradation.

Our NMR data suggests that the xylan molecules we used are acetylated, which we point to in the Results section (Line 147). While the data did not allow us to disentangle whether the increase in acetate in the effluent of Sphingomonas *Leaf257 was due solely to acetylation or also due to secretion, the presence of this molecule was nonetheless a key factor in sustaining the interaction with* Pseudorhodoferax *Leaf265.*

To explore this further, we carried out an additional computational analysis that simulated the impact of secreting acetate (among other molecules, see Supplementary Figure 12) on the growth rate of Leaf257. This analysis suggested that a basal secretion of acetate could be sustained without severely impacting its growth, pointing to secretion as a possible complementary source of acetate in the effluent.

f. The absence of glucuronic acid discussion is notable, as it is a key xylan sidechain in Arabidopsis and hardwood species.

We thank the reviewer for this point and have clarified the absence of additional sidechains in the Discussion (Line 339).

g. In line 370, the authors state, "Though the bioavailability of complex polymers like xylan may vary across the leaf surface…." This is problematic since xylan is not typically available on leaf surfaces—it is a major secondary cell wall component. This significantly impacts the discussion's key arguments and should be carefully reconsidered.?

We have modified this portion of the Discussion, which removes this statement and reframes the ecological role of Sphingomonas *Leaf257 (Line 369).*

2. NMR and MS Data:

   Solution NMR was used to identify key metabolic compounds necessary for xylan-dependent growth of Sphingomonas strains. Given its importance in tracking metabolite concentrations, the absence of NMR spectra in the main text or supplementary materials is concerning. It is essential to include spectra that support key claims, such as the presence of xylose in the effluent and significant metabolite abundance changes.

   We have now included two new Supplementary Figures (Supplementary Figures 5 and 8), which show the NMR spectra comparing key metabolites in all conditions shown in Figure 2b. We have referenced these figures in the text (Lines 142 and 158) and in the corresponding main figure, which points to the calculations of the integrals we had provided in Supplementary Table 1.

   Additionally, the Methods section describes metabolite identification through manual comparison with standards, yet key spectra should be provided in the supplementary materials for transparency.

   The spectra for xylose, alanine, and for the minimal medium with xylan have been deposited together with all collected data in the ETH Zurich Research Collection, available at doi.org/10.3929/ethz-b-000737298.

   Data Availability: The original NMR and MS datasets should be deposited in public repositories to ensure accessibility.

   We have now deposited the NMR and LC-MS datasets in public repositories. NMR data can be found at doi.org/10.3929/ethz-b-000737298, and LC-MS data and analysis scripts at github.com/VorholtLab/MicrofluidicLCMS.

   In lines 164-165, the authors mention, "...their resource and vitamin uptake profiles, measured respectively by NMR and LC-MS, differed (Figure 2b)." However, the manuscript does not clarify how these molecular concentrations were determined, nor does Figure 2b or its caption explain the analytical methods. This lack of clarity makes it difficult to interpret the findings. Additionally, it is important to mention if there are potential discrepancies between NMR and LC-MS quantification.

   We now provide more information on how the concentrations of compounds were calculated for NMR (Supplementary Table 1) and LC-MS features (Supplementary Table 2 and github.com/VorholtLab/MicrofluidicLCMS). We note that we used LC-MS to measure vitamins mainly because we had not been able to measure them using an NMR approach due to their low concentration (sub-μM range). To achieve a high degree of sensitivity for the vitamins, we undertook an LC-MS measurement approach that specifically targeted these molecules. As such, we are not able to compare any possible discrepancies between metabolites between the two measurement methodologies. We now further clarify the higher sensitivity of the LC-MS approach in the Results (Line 174).

Reviewer: 5

Summary

Pacheco et al. presents an interesting mixed computational/experimental manuscript that seeks to use metabolic models to explain community dynamics in a plant associated minimal co-culture. In principle the work approaches this question using the right system (defined media, reactor setup amenable to analysis, quantitative metabolomics, detailed metabolic models that are constrained using measurements, etc). Unfortunately, there are several shortcomings with the models and simulations that make it challenging to adjudicate the work. These should be addressed prior to evaluating the scientific merit of the work.

We thank the reviewer for the careful reading of the manuscript and thorough evaluation of the models and simulations.

Major comments

Lines 234 - 243 motivate the use of genome-scale metabolic models in this work. The authors refined previously published models from their group, and made use of computational modeling that heavily relies on accurate, detailed metabolic capabilities of each strain as captured by the models. I downloaded the models from linked Github repo (commit 2435661), and attempted to test the quality of the models, since the simulation results depend on this. Due to time constraints, I only performed spot checks on metabolically important reactions and basic simulation "sanity tests" of the two models.

The results are summarized below:

1. The ATP synthase reaction ("R_ATPS4rpp") for Leaf68 only has two genes associated with it (to the best of my knowledge 8 proteins are required for the complex to be functional). On the other hand, Leaf257 had no genes associated with this reaction. Likewise, for both models the NADH dehydrogenase reaction ("R_NADH5", "R_NADH9", "R_NADH10") has multiple isozymes, but only one or two genes associated with each isozyme. In E. coli this complex requires 16 subunits to function. Please comment on the biological realism of these reduced complexes. Alternatively, if the models are not well curated it is recommended that the authors curate all reactions in both models to ensure that the gene reaction rules are correct. This is feasible to do given the number of authors on the paper. Curation of gene reaction rules is typically a required step when reconstructing a genome-scale metabolic model (otherwise why use such a model to begin with?), even if an automatic reconstruction tool has been used. Reactions without genetic evidence should ideally not form part of the final model, unless there is compelling experimental evidence for its existence. See https://www.nature.com/articles/nprot.2009.203 for guidelines on the construction of metabolic models. The removal of e.g. the ATP synthase in Leaf257 would dramatically alter the simulation results, emphasizing the importance of curating the models used in this work.

   We thank the reviewer for this point and have updated the GPR rules in both models. As the reviewer notes, the genome-scale models used in this study are based on those previously generated in Schäfer et al.*, 2023 (DOI: 10.1126/science.adf512). In that study, 224 draft models were first generated using the tool CarveMe (Machado* et al.*, 2018, DOI: 10.1093/nar/gky537), and were then gap-filled using the tool NICEgame (Vayena* et al.*, 2022, DOI: 10.1073/pnas.2211197119) as part of a purpose-built pipeline that relied on extensive phenotypic data for each bacterial isolate. This experimental data – which determined the ability of each corresponding organism to utilize each of 44 carbon sources for growth – was critical for reproducing the carbon use capabilities of the strains, which had not been possible with models generated based solely on genomic information (Schäfer* et al.*, 2023, Fig. 3A).*

   We agree with the reviewer that reactions without genetic evidence should ideally not form part of a metabolic model. Indeed, in addition to relying on established tools, we had followed the

principles in Thiele et al.*, 2010 – as the reviewer suggests – during the reconstruction process to achieve a sufficient degree of physiological representativeness. Though the gap-filling process resulted in the addition of reactions unlinked to specific subunits in the genome, we emphasize that the function of the resulting reactions – which were necessary to recapitulate growth on each experimentally-tested substrate – is not impacted. We also undertook an additional validation step using the tool MEMOTE (Lieven* et al.*, 2020, DOI: 10.1038/s41587-020-0446-y) for all the models to ensure quality metrics like stoichiometric consistency, charge balance, and biomass reaction consistency were met. These reports are available at github.com/VorholtLab/i-At-LSPHERE/tree/v1.0/Models/Reports.*

In this study, we have further refined the models by incorporating information on vitamin production and auxotrophies – important aspects of bacterial physiology that remain difficult to determine from genomic data alone (see point 8 below). Additionally, we have now re-analysed the corresponding genomes and draft model reconstructions to map additional gene-reaction rules, as suggested. This has allowed us to, for example, link additional genes to the NADH dehydrogenase reactions mentioned by the reviewer and confirm that eight sequences for ATP synthase are present in the genome annotation for Leaf257 that we applied in our past and present study (WP_007406947.1, WP_056430567.1, WP_056430573.1, WP_056430575.1, WP_056431330.1, WP_056431333.1, WP_056431690.1, and WP_056431901.1 - they had initially been missed by CarveMe, shortcomings are documented, e.g., in DOI: 10.1038/s41540-024-00384-y, 10.1038/s41559-022-01939-0). The ATP synthase reaction had thus been added during gap filling based on functional information (Schäfer et al*. 2023), and we have now manually linked these specific genes.*

We have now also included the protein FASTA files that had been used in Schäfer et al. *to generate the corresponding draft model reconstructions in the GitHub repository (https://github.com/VorholtLab/PhyllosphereDynamics/tree/main/Models/Genomes).*

2. Next, I inspected the default bounds of reactions in the models. For the Leaf257 model the formate, H2O, and CO2 exchanges ("R_FORt2", "R_FORtex", "R_FORt", "R_H2Otex", "R_CO2tex") were artificially bounded. Please motivate why these bounds are necessary, how the actual bound values were calculated/estimated, and explain what the effect of removing them has on the simulation results.

The NMR measurements of the effluent of Leaf257 cultivated in the microfluidic device, as well as of the supernatant of Leaf68 cultivated in this effluent, allowed us to constrain the corresponding metabolic models to reflect the carbon uptake and secretion profiles observed experimentally. Importantly, this informed steps to limit the secretion of additional metabolic byproducts that were not observed, thereby constraining the solution space for the interaction simulations within COMETS.

This consideration influenced the constraint added for CO_2, which had been incorporated to ensure physiologically realistic rates of respiration (following Orth et al.*, 2011, DOI: 10.1038/msb.2011.65) and reduce the potential for secretion of additional fermentation byproducts not observed experimentally. We considered this factor to be especially important to control for the simulations in Figure 3d, which included cases of extreme rates of carbon secretion. Following the reviewer's suggestion, we carried out additional simulations without this constraint (as well as without the constraint on formate and H_2O, see below), which showed no impact on the interaction outcomes at rates of carbon and vitamin output corresponding to those estimated from experiments. We have therefore removed it from those simulations and updated the relevant figure.*

In addition to xylose, alanine, and acetate, the NMR results had also suggested the possible output of formate by Leaf257 (Supplementary Table 1). Though our measurements did not allow us to conclusively determine whether this detection of formate was indeed derived from Leaf257 (versus trace amounts present in the medium), we had adjusted the bounds of the metabolic model to

enable a basal secretion of this compound. Despite this, the simulations did not predict the exchange of formate between the strains.

The bound for H_2O had been previously set as part of the model curation process and has been removed from all simulations.

As noted above, re-running of the simulations without the bounds for these three metabolites (labelled as 'without additional constraints in the figure below) showed a minimal effect on the key dynamics and no effect on the endpoint relative abundances in the interaction between Leaf257 and Leaf68 compared to the simulations in the initial submission of this work (labelled as 'with additional constraints in the figure below). As the simulation framework was further modified (i.e. removing growth rate upper bounds for the organisms) in response to other points, the final simulations in the re-submitted manuscript differ slightly from those shown here. We have also expanded the Methods section to more clearly describe the model definition and simulation process.

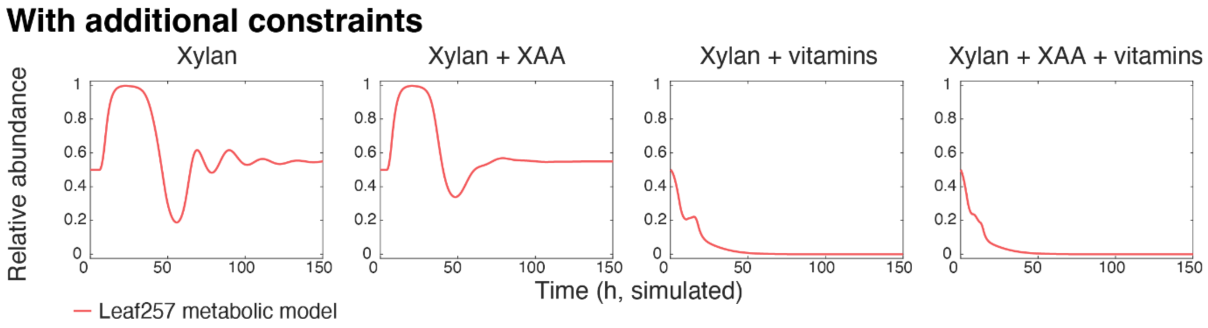

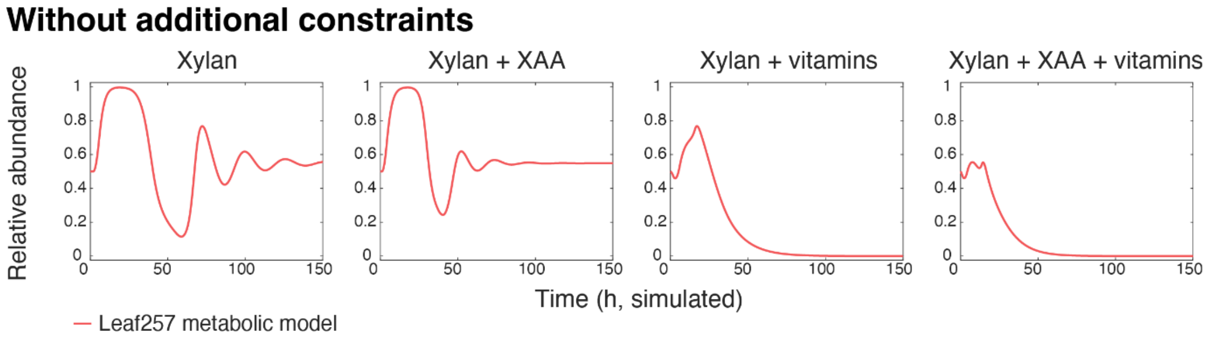

3. For the other manually set bounds in the model, please show the calculations used to find these values. It was not clear how the authors went from the results in Figure 2.b (concentration measurements) to bound limits (flux limits). Since all the simulations were generated by varying these bounds, it seems important that they are better explained.

   We thank the reviewer for pointing this out. We have now substantially expanded the Methods section on how these bounds were calculated (Line 543).

4. I had some problems connecting the actual genomes of the organisms with the accession numbers listed in the gene reaction rules. The manuscript references Schäfer et al., which links to BioProjects PRJNA297956 and PRJEB47672. I downloaded the predicted protein sequences for Leaf68 (https://www.ncbi.nlm.nih.gov/protein?term=txid1736231[Organism]) and Leaf257 (https://www.ncbi.nlm.nih.gov/protein?term=txid1736309[Organism]). There seems to be a mismatch between all of the model proteins in Leaf68 and the online resource, and some mismatches for Leaf257 (e.g. I could find "G_WP_056433836_1" but not "G_WP_007404217_1"). Was I looking at the wrong files, or are the models incorrect?

This is an important point that touches on another key consideration for the model generation process. The version of the annotation used to generate the model for Sphingomonas *Leaf257 indeed contains an entry for both WP_056433836.1 and WP_007404217.1, which correspond to a partial electron transfer flavoprotein subunit alpha/FixB family protein and subunit J of an NADH-quinone oxidoreductase, respectively. We have now included the protein FASTA files for both models in the GitHub repository (https://github.com/VorholtLab/PhyllosphereDynamics/tree/main/Models/Genomes).*

The models from Schäfer et al.*, 2023, which the models in this study directly build upon, were indeed generated using the genomes in BioProjects PRJNA297956 and PRJEB47672. However, NCBI carries out periodic re-annotation of prokaryotic genomes in RefSeq using an in-house pipeline (https://www.ncbi.nlm.nih.gov/refseq/about/prokaryotes/reannotation/), which can result in mismatched protein annotations from version to version. To ensure reproducibility as the curation process was being developed in Schäfer* et al.*, we made the decision to generate all models using a single version of each genome annotation, which in this case were downloaded from RefSeq in 2022. These genomes are available in https://github.com/VorholtLab/i-At-LSPHERE/tree/v1.0/Models/Genomes, together with the complete reconstruction pipeline and resulting models and reports.*

While we acknowledge that the continuous updating of protein annotations by NCBI can lead to incremental improvements in the completeness of resulting models, the work in Schäfer et al. *underscores the outsized importance of incorporating experimentally obtained phenotypic data to obtain a functionally representative reconstruction. In the present study, we incorporate additional information on vitamin auxotrophies and carbon conversion rates to more specifically investigate the interplay between these mechanisms in leading to different population dynamics, building on the knowledge of carbon utilization assessed previously.*

Lines 246 - 322 deal with the simulation results.

5. The authors made use of COMETS to simulate their models. This is a very sophisticated simulation tool, combining dFBA with, possibly spatial, differential equations. As such, I believe it is necessary that the authors mathematically describe (with equations) the exact system(s) they are integrating. Does it include spatial constraints, which equations are used to model uptake/secretion rates, diffusion, etc. Currently it is unclear, and the methods section does not provide enough detail. It is not enough to simply supply a settings file used to run COMETS, as this obscures the reasoning behind the simulation construction effort.

   We have expanded the section in the Methods that describes the COMETS simulations, including a mathematical description of how uptake rates were dynamically calculated (Line 564).

6. The authors describe that they constrained the growth rates of the organisms in their models. This is very unusual for constraint-based simulations, as typically a limiting nutrient uptake rate is fixed. Moreover, since they are using a dFBA-like tool to simulate their models, I would expect that it would be more natural to include xylan flux/concentration as the limiting constraint. This has the benefit that it can be directly measured in their setup. Why did the authors fix maximum growth rates? This might "bake in" the co-existence or oscillation results, and should be investigated.

   We acknowledge that the description of this constraint needed clarification and thank the reviewer for pointing this out. Importantly, we wish to note that the biomass reactions had not been constrained to single fixed values. Instead, we had only placed an upper bound on this reaction for each organism to avoid implausibly high growth rates.

However, we agree with the reviewer that it is more appropriate to rely on nutrient uptakes to compute growth rates dynamically in COMETS, based on the concentrations of external resources. We have therefore now re-run all simulations without any bounds on the biomass reactions, observing that the key predicted interaction outcomes between the models remain unchanged. Furthermore, we have clarified this aspect in the Methods (Line 554).

7. Further, the authors measured the maximum growth rate of each organism in Figure S7, but they did not use the same media. This is not a fair comparison. Since Leaf68 is necessarily growth limited by vitamins (e.g. Fig S4), it would never reach the maximum growth rate calculated in their results, because the media is not representative of the culture conditions (Fig S2 seems far more appropriate, where are the growth rates calculated from these plots?). For this reason, I don't think currently used growth rate constraints are appropriate for this system. A Michaelis-Menten-like description of the growth rate vs. vitamin concentration seems more reasonable, especially given that dFBA actually models extracellular metabolite concentrations. Please justify.

   We have now removed all growth rate constraints for the models in COMETS, as described in the point above, allowing the growth rates to be dynamically calculated using extracellular metabolite concentrations. We nonetheless note that we still utilize the growth rate measured in vitro *for* Sphingomonas *Leaf257 (Supplementary Figure 14) to set lower bounds of carbon and vitamin output from this model as now described in the Methods (Line 543).*

8. The authors also find that vitamin auxotrophies are the primary determinant of community formation (lines 284 - 290 + 310 - 317). Is this not obvious from the outset, especially given access to the metabolic models which can be directly probed for vitamin, amino acid, etc. auxotrophies (cf. lines 118 - 121 which points towards a vague "xylan-based metabolic interactions")? Since Leaf68 is auxotrophic for vitamins, how can its growth not depend on the secretion of vitamins by Leaf257? A more important question is, why does Leaf257 secrete these costly products, how does it benefit?

   We agree with the reviewer that the phrasing used when describing growth of the strains may have overemphasized the role of xylan itself. To clarify, our selection of Sphingomonas *Leaf257 and* Rhizobium *Leaf68 builds on previous knowledge of an interaction between them that was observed* in situ *(Hemmerle* et al.*, 2022, DOI: 10.1038/s41467-022-30469-3, Lines 64 and 410). That study determined that Leaf68 lacked the gene to produce the biotin precursor dethiobiotin and described a shift in the metabolism of Leaf257 toward the degradation of xylan. By studying this pair in the microfluidic device and with metabolic models, we now investigate how the different components of the interaction (carbon/vitamins) can contribute to population dynamics* in vitro*. We have removed the statement pointed out by the reviewer in favour of a simplified transition (Line 10) as well as a modified heading for that section (Line 62).*

   As the reviewer suggests, an investigation of the genome annotations of the four strains we focused on in this study provided some information on their auxotrophy status (Supplementary Figure 9). Though the specific genes we investigated had been previously determined to contribute to experimentally validated auxotrophies (Ryback et al.*, 2022, DOI: 10.1038/s41396-022-01303-x), their presence or absence can only provide an initial hypothesis on a biosynthetic deficiency. This gap is in part due to variations in – or incomplete annotations of – cofactor biosynthesis pathways, and is an area of active research and discussion within the metabolic modelling community (e.g., DOI: 10.1038/s41559-022-01939-0, DOI: 10.1038/s41467-023-43435-4, DOI: 10.1038/s41467-023-40360-4). These sources of uncertainty can lead to incorrect predictions of auxotrophies in metabolic models, as was the case for Leaf68 where it was necessary to manually incorporate the auxotrophies based on experimental evidence (despite having used CarveMe as an initial draft model generation tool, which has been reported to over-predict amino acid auxotrophies, DOI: 10.1038/s41559-022-01936-3). As such, we remain unable to confidently ascribe an auxotrophy status to an organism based solely on genomic or modelling evidence.*

The reviewer also brings up a very important point that raises broader questions about the benefits – if any – that Leaf257 may receive through its output of vitamins. Though rare, the existence of positive interactions across different phyla of Arabidopsis *leaf-associated bacteria (Carlström* et al.*, 2019, DOI: 10.1038/s41559-019-0994-z; Hemmerle* et al.*, 2022, DOI: 10.1038/s41467-022-30469-3; Schäfer* et al.*, 2022, DOI: 10.1038/s41564-022-01132-w), together with widespread vitamin and amino acid auxotrophies (Ryback* et al.*, 2022, DOI: 10.1038/s41396-022-01303-x), hints at instances of vitamin complementation that go beyond those derived from Leaf257. While a mechanism for this complementation has yet to be defined* in situ*, our experimental and computational results indicate that a relatively low concentration of vitamins (i.e., in the nM range) is sufficient to sustain the growth of a recipient strain, suggesting either leakage or lysis as sources of these molecules. This hypothesis is supported by recent work in marine communities (Gregor* et al.*, 2024, DOI: 10.1101/2023.10.16.562604) and from our group (Ryback and Vorholt, 2023, DOI: 10.1016/j.jbc.2023.104919), together implying a negligible metabolic cost to the providing organism. Sphingomonads such as Leaf257 are consistent colonizers of* Arabidopsis *leaves; however, they tend not to dominate in complex communities (> 0.1%, Pfeilmeier* et al.*, 2021, DOI: 10.1038/s41564-021-00929-5, Fig. 1e). This may suggest that a feedback effect similar to what we observe* in vitro *may also occur on plants. Here, fast-growing auxotrophic members like Leaf68 (which colonizes at >10% relative abundance) may quickly reach abundances higher than 'provider' strains like Leaf257 do, but are still dependent on the provided metabolic benefits, leading to coexistence.*

In addition to expanding on these points further in the Discussion (Line 369), we have carried out an additional flux variability analysis that quantifies the impact of secretion of the carbon sources and vitamins we studied on the predicted growth rate of Leaf257 (Supplementary Figure 12).

9. The authors claim their simulations demonstrate oscillations. However, this is difficult to ascertain from Fig 3c (which itself is too compact) as the oscillations seem much more like a differential equation solver encountering numerical issues. Did the authors substantially (e.g. 2 orders of magnitude) reduce the step size to check that this is not just a numerical artifact? The period of oscillation also seems very short (or long, it is impossible to see in the figure), is this biologically realistic? How does it compare to the oscillations of Fig 3a? Additionally, Fig 3a (Leaf257-Leaf68) shows that Leaf257 has a high/oscillating abundance, but the oscillating simulations show a low abundance for Leaf257. Do the models incorrectly predict the abundances?

We thank the reviewer for this important point. The predicted oscillations are not numerical artifacts, though we agree that the small panels in Figure 3 make it difficult to distinguish the patterns in detail. To address this, we have added a panel to Figure 3 that shows a representative simulated time course in more detail. As should now be visible, this step size is much smaller than the period of any oscillations that emerge between the two models. We have also now provided the data for all simulations as Source Data files. We utilized a step size of 0.05 h based on previous implementations of COMETS for simulating bacterial interaction dynamics (Dukovski et al.*, 2021, DOI: 10.1038/s41596-021-00593-3) and further inspected the simulated time courses to ensure that the predicted oscillations are not numerical artifacts.*

On a broader note, we wish to emphasize that our main goal in employing a modelling approach was to determine whether and how oscillations (either dampened or sustained) can emerge from bacterial interactions based on metabolic complementation, as well as how these dynamics can be altered by changing available resources (Lines 11, 233, 304, 351). As a key component of the interaction that we investigated hinges on cofactor complementation, we found that a methodology that leverages genome-scale modelling was especially useful as it more explicitly simulates the mechanisms of vitamin incorporation vis à vis carbon uptake.

To do this, we used previously gathered data on carbon source utilization (growth/no growth on 44 individual carbon sources) together with new knowledge on carbon output and vitamin leakage rates to extensively constrain the space of possible interactions that can emerge from the simulations. Nonetheless, substantial uncertainty remains within this solution space, due either to

features inherent to the microfluidic device or to physiological aspects of the organisms that remain unknown or unable to be represented using a constraint-based approach. This latter aspect is especially challenging for modelling environmental strains like Leaf257 and Leaf68, although models for organisms that have been extensively characterized also suffer from this issue (see, e.g., Bernstein et al.*, 2023, DOI: 10.15252/msb.202311566, for an example on* E. coli *and vitamin requirements). The variation of carbon/vitamin output rates by Leaf257 in Figure 3 is thus intended to help contextualize this uncertainty in the space of interactions that can emerge between simulated Leaf257 and Leaf68, which also allowed us to investigate the features underlying an emergent oscillatory or coexistence pattern.*

Following the reviewer's point on the impact of different starting abundances discussed below, we have now also carried out a new set of simulations that investigates the robustness of the fluctuating species abundance pattern. We nonetheless feel it is currently beyond the goal of this work to explicitly recapitulate the period/amplitude of any oscillations, and have added a point in the Discussion to clarify this limitation (Line 362).

10. Fig 4a and 4b aim to demonstrate concordance between simulation results and experimental observations. In the trivial cases where the faster growing organism has all the required nutrients the data match (sort of), but for the case where only carbon sources are present the data do not match at all. For example, the time-scale is completely different (by an order of magnitude), the abundance time course profile is not similar (the initial conditions do not even match the experimental data), and steady state is not reached for the experimental setup, making it hard to compare the results. Since the purpose of the work is to use models to investigate metabolic connection points in a co-culture, it stands to reason that the models should broadly match observations. Please justify the suitability of the simulations given this.

    We continue from our discussion in the previous point in emphasizing that, by varying the medium composition, we sought mainly to predict outcomes of coexistence or outcompetition that we validated experimentally. However, we note that there was an error in formatting Figure 4a that affected the scale of the x-axis (the axis should display as ranging from 0 to 150 h, not 500 h). Though the underlying simulations had the correct timescale, we have re-run them following the reviewer's recommendations in points 2, 6, and 7 and have corrected the corresponding axes.

11. Do the initial abundances make a difference for the predicted dynamics?

    To investigate this point, we have now run a new set of simulations in which we vary the initial abundances of the two models to quantify differences in dynamics. Here, we used the same simulation conditions and medium composition as in Figure 3 (i.e., a minimal medium with 10 mM xylan as sole carbon source and without vitamins), but varied the initial abundances of the Leaf257 and Leaf68 metabolic models from 0.005 to 10 times their initial abundances in Figure 3. The results, described in Supplementary Figure 11, highlight that an outcome of coexistence – following population abundance fluctuations – is largely robust to the initial population sizes. This is with the exception of very small initial concentrations of Leaf68, in which the population is unable to establish. Nonetheless, increasing the initial population of Leaf257 was predicted to alleviate this effect, which (similar to the predictions in Figure 3) points to a minimum amount of metabolic products that are necessary for the recipient strain to grow. Moreover, simulations with a lower initial population of Leaf68 predicted that Leaf257 would initially remain dominant for a longer amount of time, which was followed by the growth of Leaf68 and an eventual stabilization of the populations. We now discuss these results in the main text as well (Line 296).

12. The simulation output data supplied by the authors on Github is not presented in a format amenable to simple inspection. Could they please save their results as CSV files (e.g. biomassLog, fluxLog, etc.) that can be loaded into a tabular format? Additionally, could they please upload all the data used to make the figures (e.g. Fig 3c), and clearly explain how to reproduce their figures using the CSV files? The current state of the Github repo is not sufficient for this purpose.

We have submitted the logs (biomass, media, and flux) from all simulations as Source Data files in CSV format, grouped by each corresponding figure panel. We have also submitted the underlying data for all main figures as Source Data. The MATLAB script necessary to process and plot the simulation results has now also been added to the GitHub repository (github.com/VorholtLab/PhyllosphereDynamics).

Reviewer: 4

The authors have appropriately addressed my comments. For those points that could not be fully resolved within the current experimental framework, they have provided clarifications. I would like to emphasize that xylan decorations and sidechains are critical determinants of xylan's chemicophysical properties and represent a key distinguishing feature among xylans from different plant species. The additional clarifications related to xylan sidechains included in the revised manuscript are therefore important.

We thank the reviewer for the important points raised and are glad to have been able to satisfactorily address them in our previous revision.

Reviewer: 5

Summary

The reviewer would like to thank the authors for addressing the raised concerns. The additions in the methods section really improved the clarity of the work, and the caveats added to the discussion, explaining a possible origin of the vitamin leakage, helps ground the simulations. However, there are some remaining fundamental/major questions that I would like to see addressed.

We appreciate the positive feedback on the clarifications and new simulations we performed, and thank the reviewer for the detailed assessment of our work.

Major issues

- Model curation

  In the manuscript, as well as the rebuttal, the authors state that their work uses curated models (e.g. line 236) and these models "rely on detailed knowledge of the metabolic capabilities and requirements of each strain". Unfortunately this is not a fair or accurate characterization of the models, instead the models are automatically reconstructed (CarveMe) and gap filled using experimental data (NICEgame) – no meaningful curation was performed.

  Amongst other steps, curation generally entails verifying the gene-protein-reaction rules suggested by automatic tools that generated the draft models (see steps 13 & 18 of the protocol by Thiele et al., 2010 that the authors used to build their models). This is an especially important step due to the numerous and well known problems with automatic tools, as stated by the authors themselves in their rebuttal.

  In the first review I drew attention to the fact that the gene-reaction-rules seem largely uncurated. I pointed out that the ATP synthase and the NADH dehydrogenase assignments were physiologically impossible. The authors fixed the problems with the ATP synthase, but not with the NADH dehydrogenase (reaction IDs: R_NADH5, R_NADH9, R_NADH10). In the rebuttal the authors state that these reactions were added during the gap filling process. However, these reactions are *not* gaps in the model. The proteins are present in the annotation files supplied by the authors, and by simply searching for "NADHquinone oxidoreductase" one may find the subunits of the NADH dehydrogenase for example.

  Metabolic gaps occur when you cannot assign an enzyme to a reaction (due to a lack of annotations) but experimental evidence suggests the reaction is present, not when the the model is insufficiently curated. While omissions like this in secondary metabolism are more understandable, these problems occur in central/core metabolism, and reduce the confidence I have in trusting any model predictions.

  Further examples (randomly chosen) demonstrating that the models are not curated include:

  a. the five isozymes listed under fructose-bisphosphatase (R_FBP) in Leaf257:
     - G_WP_056430038_1: class 1 fructose-bisphosphatase
     - G_WP_056430890_1: class II fructose-bisphosphatase
     - G_WP_056434344_1: Cof-type HAD-IIB family hydrolase (?)
     - G_WP_201780611_1: 3′(2′),5′-bisphosphate nucleotidase CysQ (?)
     - G_WP_236696918_1: 3′(2′),5′-bisphosphate nucleotidase CysQ (?)
  b. the single protein assigned to pyruvate dehydrogenase for Leaf68:
     - G_WP_062424881_1: dihydrolipoyl dehydrogenase

- this reaction occurs through a complex, and I could find at least 3 more proteins that should be associated with this reaction

c. the pyruvate dehydrogenase reaction (R_PDHcr) in Leaf257:
   - three monomeric isozymes are listed under its gene-reaction-rule, despite the reaction being catalyzed by a complex
   - I easily found 4 other proteins associated with this complex (search for "pyruvate dehydrogenase" in the protein annotation files the authors included in the github repo)
   - it seems that PDH is present in Leaf257, but the overall reaction "coa_c + nad_c + pyr_c ⇌ accoa_c + co2_c + nadh_c" is not what R_PDHcr catalyzes. Instead, R_PDHcr seems to break the overall reaction into substeps, but I am not sure if the overall reaction is then present in the final model?

The authors heavily rely on a phenotypic screen used in Schäfer et al. 2023 to justify the inclusion of reactions where e.g. only one protein was assigned to a reaction known to require multiple subunits (e.g. R_PDHcr in Leaf257). While useful, such screens are not a replacement for manually making sure the modeled metabolism reflects the current state of knowledge of the organism. It is easy to think of examples where a core enzyme can be removed from the model, but the model still predicts viability on all the tested substrates. This is particularly important because the authors simulate the entire metabolism of each organism in the current work to make predictions about growth rate, substrate consumption and production etc (e.g. the results discussed in lines 273 – 288).

I believe the authors need to either curate (at least) the primary metabolism of the models they use, or make it explicit in the description of the models that they are largely uncurated and the genetic evidence for reactions was deemed not important for this work. If they opt for the latter, it also raises the question of why genome-scale models were used in the first place.

We thank the reviewer for the thorough evaluation and have incorporated additional gene-protein reaction rules throughout the metabolic models, as requested. We have also verified that all transformations related to central carbon metabolism in both models (i.e., glycolysis, the pentose-phosphate pathway, and the TCA cycle) have valid GPR rules, as determined by manually inspecting genome annotations.

We acknowledge that our previous use of the term "curated" was not precise enough. As the reviewer noted, we initially generated the models using CarveMe (Machado et al., Nucleic Acids Res.*, 2018), a draft reconstruction tool. We then refined them using phenotypic data and the gap-filling tool NICEgame (Vayena* et al., PNAS*, 2022) to achieve high functional accuracy (98% balanced accuracy across 224 models and 45 carbon sources; Schäfer* et al., Science*, 2023) and phylogenetic representativeness (Schäfer et al., Fig. 3D). In the present work, we incorporated vitamin requirements and secretion capabilities based on our experimental observations (original submitted version) and performed additional manual curation of GPR rules for central metabolism, following the reviewer's recommendations.*

We have now revised the wording in the Results section (line 261) and the Methods (line 586) to more precisely capture these steps, and have updated the models on the GitHub repository to reflect the additional GPR rule curation.

To the reviewer's specific points:

a. This has been corrected, with the updated gene-reaction rule for R_FBP now as follows: "(WP_056430038_1 or WP_056430890_1)". These annotations correspond to a class 1 fructose-bisphosphatase and a class II fructose-bisphosphatase, respectively. The spurious annotations (HAD-IIB family hydrolase and CysQ) have been removed.

b. *The overall reaction coa_c + nad_c + pyr_c ⇌ accoa_c + co2_c + nadh_c is carried out by R_PDH, which oxidizes pyruvate and transfers the acetyl group to CoA. We have thus linked the following genes to the reaction: WP_062427379 (pyruvate dehydrogenase (acetyl-transferring) E1 component subunit alpha), WP_062437398 (pyruvate dehydrogenase complex E1 component subunit beta), and WP_062437399 (pyruvate dehydrogenase complex dihydrolipoamide acetyltransferase). The separate reaction R_PDHcr regenerates lipoamide for the pyruvate dehydrogenase complex and corresponds to the annotations WP_062424881 or WP_062437403 (each a dihydrolipoyl dehydrogenase).*

c. *Similar to the model for* Rhizobium *Leaf68 above, we have applied the corrected GPR rule for R_PDH in the model for* Sphingomonas *Leaf257. This reaction comprises WP_056432896 (pyruvate dehydrogenase (acetyl-transferring) E1 component subunit alpha), WP_056432899 (pyruvate dehydrogenase complex E1 component subunit beta), and WP_056434262 (pyruvate dehydrogenase complex dihydrolipoamide acetyltransferase). The separate reaction R_PDHcr corresponds to the annotations WP_056434257 or WP_056437006 (each a dihydrolipoyl dehydrogenase).*

Beyond the examples highlighted by the reviewer, we have also corrected GPR rules for NADH dehydrogenase by linking these reactions to the appropriate subunit annotations identified in the genome annotation files. We have additionally reviewed and corrected GPR associations for other central metabolic enzymes to ensure they accurately reflect the known complex structures.

- Simulation parameters

a. Dynamic uptake rates

The authors used a Michaelis-Menten like equation to simulate metabolite uptake rates through COMETS:

$$v_{max} = v_{max}\left[\frac{M}{M + K_M \cdot W^3}\right]$$

where v_{max} = 1 000 mmol g_{DW}^{-1} h^{-1}, K_M = 20 mmol cm^{-3} = 20 M, and W^3 = 10^{-6} cm^{-3} from their methods section. Figure 2 reports concentration measurements the authors found in their device, e.g. xylose around 0.2 mM, alanine around 0.05 mM, vitamins around 10-100 nM. Thus, for the concentration measurements in the experimentally observed range the uptake flux never saturates since $K_M \gg$ all reasonable metabolite concentrations. Is this reasonable? If so, why bother with the Michaelis-Menten kinetics?

Additionally, the references used to justify these parameter choices (line 574, references 28 and 29) are at odds with the content of those papers. For example, Harcombe 2014 uses v_{max} = 10 mmol gDW^{-1} h^{-1}, K_M = 10 µM = 10 × 10^{-6} mmol cm^{-3} and the chemostat simulation in Dukovski et al. 2021 uses v_{max}= 15 mmol gDW^{-1} h^{-1}, K_M = 0.1 mM = 10^{-4} mmol cm^{-3}. Why are the parameter choices here so different from the papers the authors reference to justify their choices? I suspect changing the metabolite uptake kinetics would have a dramatic effect on their results.

This is an excellent point, and we realize that the way the equation is presented can lead to confusion. We selected these parameters to ensure that we were operating within the linear regime of Michaelis-Menten kinetics (where M << K_M), and to achieve physiologically realistic uptake rates (on the order of 10^0 – 10^1 mmol/gDW/hr for xylose, in line with values for sugar monomers applied in Harcombe et al. *and Dukovski* et al.*) while avoiding assumptions about strain- and*

metabolite-specific K_M values that are unavailable. This was necessary because COMETS does not have an explicit linear implementation. We have now updated the relevant section of the Methods to clarify this (line 620).

b. Measured fluxes

Why was the production flux of the various metabolites used in the study not directly calculated based on the data the authors have? Since the authors measured the concentration of each metabolite (Fig 2 A), knew the flow rate in their device (and its dimensions), and have images to estimate biomass (even cell counts), why not just directly calculate the secretion fluxes? This would be very useful to narrow down the 4 orders of magnitude the parameters cover in their simulations.

Additionally, the authors state on lines 266 – 268: "This regime, characterized in part by carbon and vitamin exchange at similar orders of magnitude to those estimated from experimental data…" needs to be clarified do the authors mean the estimates described by lines 543 –551? These fluxes are not measured but computationally predicted by constraining a flux balance analysis model such that the ratio of predicted fluxes match the ratio of metabolite concentrations (based on fig 2). In this simulation, the maximum growth rate is both upper bounded and the objective of the simulation at the same time (why not just run pFBA to get a unique solution?).

Additionally, these simulations to estimate the fluxes will only be accurate if parameters like the ATPM (NGAM coefficient) and growth associated ATP maintenance (GAM) function are correct for the organism being modeled. In the protocol by Thiele et al. 2010, steps 24 – 34, this is detailed and highlighted as an important component of generating a high quality metabolic model. Did the authors attempt to estimate this for their organisms? I would expect these parameters to also be relatively important for community simulations, as the energy budget would directly affect the biomass yield of each community member.

We appreciate the reviewer pointing out this area for improvement to increase clarity. Although we indeed measured the concentration of each metabolite, we did so on the scale of entire microfluidic devices, each of which consists of 200 microchambers. Technically, it was not possible to image all the microchambers simultaneously, which prevented us from determining the total biomass within the devices. Even if we could extrapolate based on the data from the representative microchambers we imaged, we could not account for the cells in the connecting channels adjacent to the microchambers (Supplementary Figure 2). However, aggregating the metabolic efflux stemming from an entire device made it possible to identify differences in metabolite concentrations that would not have been detectable at the scale of individual microchambers. This approach enabled us to estimate population-level ratios of metabolic conversion, as described in the Methods (line 590).

We relied on the aforementioned approach to calculate the lower bounds of secretion for each of the exchanged metabolites. In this approach, we enforced experimentally estimated metabolite conversion ratios with a bounded biomass flux that was informed by an in vitro *growth assay (Supplementary Figure 15). We consider this an appropriate approach in light of the goal of simulating whether and how oscillation patterns can occur in relation to alternative outcomes of coexistence or outcompetition.*

We recognize that the wording in the Results section should have been more precise, and have amended it accordingly (line 293): "This regime, characterized by rates of carbon and vitamin output at similar orders of magnitude to those used to constrain the model based on experimental data."

- Analysis of simulations

The authors make use of a very sophisticated modeling platform, COMETS, to run their simulations. A major advantage of this approach is that it should predict extracellular metabolite concentrations, which can be compared to the measurements of Fig 2 to validate the physiological realism of their simulations. How do the extracellular metabolite concentrations across each simulation in Fig 3. D compare to what the authors measured? Does this change if the Michaelis constant (discussed above) is changed to a more reasonable value?

Thank you for this suggestion. Indeed, our analysis of the cross-correlations between the uptake flux of Rhizobium *Leaf68 to its biomass flux in the COMETS simulations recapitulated key elements observed in the experiments: its dependence on the vitamins produced by* Sphingomonas *Leaf257, its uptake of the exchanged xylose and alanine, its degradation of the xylan oligomers, and its lack of acetate uptake (Figure 3f). To further illustrate these interaction features, we have now included the media logs corresponding to the three environmental conditions shown in Figure 4 – as well as the xylan-only condition shown in Figure 3e – as a new Supplementary Figure (Supplementary Figure 12) and incorporated them into the text (line 349).*

We nonetheless refrained from directly comparing the results presented in Figure 2b with metabolite concentrations predicted by the COMETS simulations for two primary reasons. First, the metabolomic measurements for the interactor strains were obtained after their independent cultivation on effluent derived from the growth of Sphingomonas *Leaf257 in the microfluidic device, rather than during simultaneous co-culture within the same chambers. Second, the COMETS simulations were not intended to explicitly predict metabolite abundances within the microfluidic system. This is partly due to experimental constraints on accurately estimating total biomass, as discussed in response to the reviewer's previous point.*

We undertook a metabolic modeling approach primarily due to the aforementioned experimental limitations to better understand how different population dynamics could arise based on the measurable physiological characteristics of individual strains.

The authors use Leaf68 in their simulations to show the different possible outcomes in coculture. Do the same trends hold if the other organisms are modeled like this (e.g. Leaf203 or Leaf265 that are also auxotrophic for vitamins)? I suspect the exact same dynamics would occur since the models are likely extremely similar, yet we see the oscillations only in the Leaf68 community experimentally (Fig 3 A). Here, I think the simulations and modeling reported by the authors can be used to generate (alternative) hypotheses that experimentalists can test to understand/validate the mechanism being proposed here.

I appreciate the discussion on lines 273 – 288 about the cause of the oscillations, but more details and precision can be added here. What simulation conditions predict the onset of sustained vs dampened oscillations? From Fig 3 D it seems that higher vitamin leakage causes the oscillations, but experimentally the only vitamin secretion comes from Leaf257, which likely does not adjust its vitamin secretion based on the partner microbe (or does it?). Why do we not see the same oscillations for Leaf203 or Leaf265? Is there another mechanism generating the oscillations? Do any other parameter variations cause oscillations? Do the dampened oscillation communities "need less" vitamins? Are they slower growing? Are they more energy efficient (GAM or NGAM different)? What would be very enlightening is a "bifurcation" plot of the parameters vs. type of oscillation. Additionally, my current understanding of the model is that growth rate bounds have been removed, but on lines 357 – 358 the authors posit that "species-specific growth rate"

differences contribute to the modeled oscillations. How does this follow given their models and how was it tested computationally?

Indeed, the oscillations in our experiments were precisely why we adopted a metabolic modeling approach: to better understand whether and how they could emerge from metabolic complementation. This finding forms the basis for the discussion starting on line 270, in which we analyzed the uptake fluxes of the exchanged metabolites by Rhizobium *Leaf68 to determine their contribution to the population dynamics. This analysis allowed us to propose that the higher growth rate of* Rhizobium *Leaf68, together with its ability to utilize xylan forms the basis of the oscillations with* Sphingomonas *Leaf257. To clarify this point, we have now added the estimated growth rates of the populations in Supplementary Figure 3. The reviewer is also correct that the growth rates are unconstrained in the simulations and that the statement in the Discussion can lead to confusion. Therefore, we have removed the 'species-specific' wording (line 391).*

With regards to the reviewer's specific question regarding Microbacterium *Leaf203 and* Pseudorhodoferax *Leaf265, our evidence suggests that the oscillatory dynamics observed with* Rhizobium *Leaf68 stem from a specific combination of factors that differ across strains:*

1. *Xylan utilization capability: Unlike* Pseudorhodoferax *Leaf265, which lacks the necessary glycosyl hydrolase (Supplementary Figure 1),* Rhizobium *Leaf68 can degrade xylan once it is provided with vitamins. This creates a competitive dynamic for the primary carbon source.*
2. *Growth rate differences:* Rhizobium *Leaf68 exhibits a higher growth rate than* Microbacterium *Leaf203 when paired with* Sphingomonas *Leaf257 (Supplementary Figure 3). This enables* Rhizobium *Leaf68 to rapidly expand once vitamins become available.*
3. *Vitamin dependency stringency: The complete auxotrophy of* Rhizobium *Leaf68 for three vitamins creates tight coupling to* Sphingomonas *Leaf257's metabolic output, establishing the feedback loop necessary for oscillations.*

Moreover, Figure 3 and the associated discussion serve to systematically compare the contributions of varying carbon and vitamin output fluxes to the simulations. The reviewer brings up a very interesting suggestion for further analyzing the emergence of sustained vs. dampened oscillations. Our simulation results in Figure 3d suggest that greater vitamin output could lead to sustained oscillations, though too high of an output would lead to the collapse of both populations (line 280). However, due to technical limitations, such as the inability to run the microfluidic devices over longer time periods (the devices fail after ~50 h, line 498), and a lack of tools to modulate vitamin output, we refrain from commenting in detail on this possibility in the text.

I am also interested to see the effect uptake kinetics have on the predictions. I suspect the currently used K_M might also be behind the observed oscillations, and that saturation in uptake kinetics may dampen all the predicted oscillations. This would also be interesting as that would suggest transporter effects may also contribute to the different predicted phenotypes. The strength of modeling is that all these hypotheses can be simply tested by tweaking the existing simulations — greatly aiding our understanding of the system.

This is also a great point, and we would indeed expect the predicted dynamics to change by varying K_M values across different orders of magnitude. However, since we lack knowledge on the K_M values for each strain and relevant metabolite, we opted for a parsimonious approach, setting a global K_M value that applied to both interacting pairs, as previously applied (Dukovski et al., Nature Protocols*, 2021). This limitation also underlies our reasoning to operate within the linear range of Michaelis-Menten kinetics as described in the point above, while ensuring that the models exhibited physiologically reasonable rates of metabolite uptake (line 622).*

Moreover, setting a single K_M value enabled us to directly compare simulations in which oscillations emerged to those in which we examined the impact of altering the medium components. In doing so, we were able to assess how oscillations can emerge and make experimentally validated predictions about how interaction outcomes can be modulated or broken (Figure 4).

Minor issues

- please add the estimated growth rates to Supplementary Figure 3

  Thank you for this suggestion. The estimated growth rates have been added.

- the uptake flux in Supplementary Figure 12 needs units

  Thank you, this has been corrected. Please note that, due to the addition of the additional Supplementary Figure discussed above, these flux variability analysis results are now in Supplementary Figure 13.

Reviewer: 6

At the request of the editor, I have reviewed the authors' responses to Reviewer #1's comments and re-evaluated the manuscript.

We thank Reviewer 6 for agreeing to review our paper, as well as for carefully assessing our responses to Reviewer 1's comments.

a. First, the authors have addressed each of the points raised by Reviewer #1, particularly those related to experimental details. However, other aspects still require improvement. For instance, points six, nine, and ten concerning the rationale for strain selection and their ecological significance could be elaborated further in the Introduction.

   We have now added a new paragraph to the Introduction that addresses the scientific premise of xylan availability (line 53).

b. While the authors adopt the phyllosphere as their research focus, the experiments only use leaf metabolites as a nutrient medium. This raises the question: Do healthy plant leaves genuinely secrete oligosaccharides? The scientific premise should clarify whether the study aims to explore microbial interactions in the healthy phyllosphere or the dynamics during leaf senescence and pathogen infection. This distinction is critical, as it determines whether the study aligns more with plant microbiome research on leaf health or ecological studies on litter decomposition. The authors should explicitly define and discuss this scientific context, as this point—previously highlighted by Reviewer #1—remains unaddressed in the current revision.

   We thank the reviewer for raising this point. Our study is primarily focused on microbial interactions within the context of a healthy phyllosphere. Intact plant leaves release carbohydrates, and passive cuticular leaching of metabolites – including sugars, amino acids, and organic acids – to the leaf surface through diffusion across the cuticle has been documented in healthy leaves (Mercier & Lindow, Appl Environ Microbiol*, 2000; Leveau & Lindow,* PNAS*, 2001). Quantitative studies have demonstrated that 0.2–10 µg of sugar per leaf – sufficient to support 10^7–10^8 bacterial cells – can be recovered from uncolonized bean leaves (Lindow & Brandl,* AEM*, 2003). Oligomers are necessary for cell wall synthesis and remodeling, but the extent to which they leach has not been quantified. Also, phyllosphere bacteria have been shown to enhance nutrient availability by secreting biosurfactants that increase cuticular permeability. For example,* Pseudomonas syringae *releases syringafactin, which increases the availability of sugars for epiphytic growth (Van der Wal & Leveau,* Environ Microbiol*, 2011). The induction of cell wall-degrading enzymes, including xylanases has been shown on healthy* Arabidopsis *leaves (Hemmerle* et al., Nat Commun*, 2022). We have revised the Introduction to better introduce the evidence for xylan degradation in the phyllosphere (lines 53-66). Note that we did not include all the above references for conciseness.*

c. The experimental design, which relies solely on metabolites rather than living leaves, suggests that the authors may be leaning toward the latter scenario (litter decomposition). However, as Reviewer #1 noted, the study currently lacks a coherent scientific background to support this approach, creating a disconnect between the stated research framework and the actual experiments conducted.

   Our study is not intended to model litter decomposition specifically. Rather, our use of plant-derived xylan as a substrate reflects the known capability of phyllosphere bacteria, including the strains used in our study, to produce xylan-degrading enzymes in planta *(Supplementary Figure 1). Additionally, xylan is ecologically relevant as a substrate that structures bacterial interactions*

on leaves (Hemmerle et al., Nat Commun*, 2022). The microfluidic approach necessarily abstracts features of the natural environment in order to enable controlled observation and quantification. However, the metabolic interactions we identified – carbon cross-feeding and vitamin complementation – are based on the physiological capabilities of bacteria observed on healthy* Arabidopsis *leaves, from which the bacteria were originally isolated.*

d. I recommend relocating the background information presented in Result 1 to the Introduction. This would help streamline the Results section and improve its focus.

We agree and have moved the background information from the Results section to the Introduction.

e. In Lines 144–146, the authors state: "Due to the likely overrepresentation of undegraded xylan in the medium stemming from the continuous flow inherent to the microfluidic device, we carried out a separate experiment in a batch culture system that further confirmed this degradation." However, the results related to the undegraded xylan are not presented. The authors should include these findings.

We thank the reviewer for noting this. The referenced statement explains why we performed additional batch culture experiments – namely, to confirm xylan degradation that might be underestimated in the flow-through microfluidic system due to continuous xylan replenishment. The results of this batch culture experiment are presented in Supplementary Figure 6, which demonstrates xylan degradation by Sphingomonas *Leaf257 in a closed system (reduction in xylan peak area with corresponding increase in xylose). We have clarified this cross-reference in the revised text (line 167): "Due to the possible underestimation of xylan degradation in the effluent stemming from the continuous replenishment inherent to the microfluidic device, we carried out a separate experiment in a batch culture system that confirmed this degradation (Supplementary Figure 6)."*

f. Additionally, Sections 1, 2, and 4 of the Results could be condensed and summarized more effectively to enhance readability.

We appreciate the feedback and have condensed the Results sections 1, 2, and 4, as requested, to improve conciseness while maintaining scientific rigor. Specifically: (1) In Section 1, we streamlined the description of the interaction screen. We did this by focusing on the key finding – the identification of three Sphingomonas *Leaf257-dependent strains – and reducing redundant details about strains that showed independent growth on xylan. We also moved information to the Introduction section (in response to comment d). (2) In Section 2, we consolidated the metabolomic analysis narrative. We did this by integrating the NMR and LC-MS findings more cohesively. We also removed repetitive descriptions of experimental procedures that were already detailed in the Methods. (3) In Section 4, we tightened the description of simulation outcomes. We did this by focusing on the mechanistic insights rather than extensively describing each simulation condition. We believe these revisions improve readability while preserving the essential findings and their interpretation.*

Reviewer: 7

The authors responded appropriately to the comments raised.

We thank the reviewer for reviewing our revised manuscript, and we are glad to have appropriately addressed the previous comments.

Summary
The reviewer would like to thank the authors for addressing the raised concerns. The additions in the methods section really improved the clarity of the work, and the caveats added to the discussion, explaining a possible origin of the vitamin leakage, helps ground the simulations. However, there are some remaining fundamental/major questions that I would like to see addressed.

Major issues

Model curation
In the manuscript, as well as the rebuttal, the authors state that their work uses curated models (e.g. line 236) and these models "rely on detailed knowledge of the metabolic capabilities and requirements of each strain". Unfortunately this is not a fair or accurate characterization of the models, instead the models are automatically reconstructed (CarveMe) and gap filled using experimental data (NICEgame) – no meaningful curation was performed.

Amongst other steps, curation generally entails verifying the gene-protein-reaction rules suggested by automatic tools that generated the draft models (see steps 13 & 18 of the protocol by Thiele et al., 2010 that the authors used to build their models). This is an especially important step due to the numerous and well known problems with automatic tools, as stated by the authors themselves in their rebuttal.

In the first review I drew attention to the fact that the gene-reaction-rules seem largely uncurated. I pointed out that the ATP synthase and the NADH dehydrogenase assignments were physiologically impossible. The authors fixed the problems with the ATP synthase, but not with the NADH dehydrogenase (reaction IDs: R_NADH5, R_NADH9, R_NADH10). In the rebuttal the authors state that these reactions were added during the gap filling process. However, these reactions are *not* gaps in the model. The proteins are present in the annotation files supplied by the authors, and by simply searching for "NADH-quinone oxidoreductase" one may find the subunits of the NADH dehydrogenase for example.

Metabolic gaps occur when you cannot assign an enzyme to a reaction (due to a lack of annotations) but experimental evidence suggests the reaction is present, not when the the model is insufficiently curated. While omissions like this in secondary metabolism are more understandable, these problems occur in central/core metabolism, and reduce the confidence I have in trusting any model predictions.

Further examples (randomly chosen) demonstrating that the models are not curated include:

- the five isozymes listed under fructose-bisphosphatase (R_FBP) in Leaf257:
  - G_WP_056430038_1: class 1 fructose-bisphosphatase
  - G_WP_056430890_1: class II fructose-bisphosphatase
  - G_WP_056434344_1: Cof-type HAD-IIB family hydrolase (?)
  - G_WP_201780611_1: 3′(2′),5′-bisphosphate nucleotidase CysQ (?)
  - G_WP_236696918_1: 3′(2′),5′-bisphosphate nucleotidase CysQ (?)
- the single protein assigned to pyruvate dehydrogenase for Leaf68:
  - G_WP_062424881_1: dihydrolipoyl dehydrogenase
  - this reaction occurs through a complex, and I could find at least 3 more proteins that should be associated with this reaction
- the pyruvate dehydrogenase reaction (R_PDHcr) in Leaf257:
  - three monomeric isozymes are listed under its gene-reaction-rule, despite the reaction being catalyzed by a complex
  - I easily found 4 other proteins associated with this complex (search for "pyruvate dehydrogenase" in the protein annotation files the authors included in the github repo)

- it seems that PDH is present in Leaf257, but the overall reaction "coa_c + nad_c + pyr_c ⇌ accoa_c + co2_c + nadh_c" is not what R_PDHcr catalyzes. Instead, R_PDHcr seems to break the overall reaction into sub-steps, but I am not sure if the overall reaction is then present in the final model?

The authors heavily rely on a phenotypic screen used in Schäfer et al. 2023 to justify the inclusion of reactions where e.g. only one protein was assigned to a reaction known to require multiple subunits (e.g. R_PDHcr in Leaf257). While useful, such screens are not a replacement for manually making sure the modeled metabolism reflects the current state of knowledge of the organism. It is easy to think of examples where a core enzyme can be removed from the model, but the model still predicts viability on all the tested substrates. This is particularly important because the authors simulate the entire metabolism of each organism in the current work to make predictions about growth rate, substrate consumption and production etc (e.g. the results discussed in lines 273 – 288).

I believe the authors need to either curate (at least) the primary metabolism of the models they use, or make it explicit in the description of the models that they are largely uncurated and the genetic evidence for reactions was deemed not important for this work. If they opt for the latter, it also raises the question of why genome-scale models were used in the first place.

Simulation parameters

Dynamic uptake rates

The authors used a Michaelis-Menten like equation to simulate metabolite uptake rates through COMETS:

$$v = v_{\text{max}} \frac{M}{M + K_{\text{M}} \cdot W^3}$$

where $v_{\text{max}} = 1\,000\,\text{mmol}\,\text{g}_{\text{DW}}{}^{-1}\,\text{h}^{-1}$, $K_{\text{M}} = 20\,\text{mmol}\,\text{cm}^{-3} = 20\,\text{M}$, and $W^3 = 10^{-6}\ \text{cm}^{-3}$ from their methods section. Figure 2 reports concentration measurements the authors found in their device, e.g. xylose around 0.2 mM, alanine around 0.05 mM, vitamins around 10 - 100 nM. Thus, for the concentration measurements in the experimentally observed range the uptake flux never saturates since $K_{\text{M}} \gg$ all reasonable metabolite concentrations. Is this reasonable? If so, why bother with the Michaelis-Menten kinetics?

Additionally, the references used to justify these parameter choices (line 574, references 28 and 29) are at odds with the content of those papers. For example, Harcombe 2014 uses $v_{\text{max}} = 10\,\text{mmol}\,\text{g}_{\text{DW}}{}^{-1}\,\text{h}^{-1}$, $K_{\text{M}} = 10\,\mu\text{M} = 10 \times 10^{-6}\,\text{mmol}\,\text{cm}^{-3}$ and the chemostat simulation in Dukovski et al. 2021 uses $v_{\text{max}} = 15\,\text{mmol}\,\text{g}_{\text{DW}}{}^{-1}\,\text{h}^{-1}$, $K_{\text{M}} = 0.1\,\text{mM} = 10^{-4}\,\text{mmol}\,\text{cm}^{-3}$. Why are the parameter choices here so different from the papers the authors reference to justify their choices? I suspect changing the metabolite uptake kinetics would have a dramatic effect on their results.

Measured fluxes

Why was the production flux of the various metabolites used in the study not directly calculated based on the data the authors have? Since the authors measured the concentration of each metabolite (Fig 2 A), knew the flow rate in their device (and its dimensions), and have images to estimate biomass (even cell counts), why not just directly calculate the secretion fluxes? This would be very useful to narrow down the 4 orders of magnitude the parameters cover in their simulations.

Additionally, the authors state on lines 266 - 268: "This regime, characterized in part by carbon and vitamin exchange at similar orders of magnitude to those estimated from experimental data…" needs to be clarified - do the authors mean the estimates described by lines 543 – 551? These fluxes are not measured but computationally predicted by constraining a flux balance analysis model such that the ratio of predicted fluxes match the ratio of metabolite concentrations (based on fig 2). In this simulation,

the maximum growth rate is both upper bounded and the objective of the simulation at the same time (why not just run pFBA to get a unique solution?).

Additionally, these simulations to estimate the fluxes will only be accurate if parameters like the ATPM (NGAM coefficient) and growth associated ATP maintenance (GAM) function are correct for the organism being modeled. In the protocol by Thiele et al. 2010, steps 24 – 34, this is detailed and highlighted as an important component of generating a high quality metabolic model. Did the authors attempt to estimate this for their organisms? I would expect these parameters to also be relatively important for community simulations, as the energy budget would directly affect the biomass yield of each community member.

Analysis of simulations

The authors make use of a very sophisticated modeling platform, COMETS, to run their simulations. A major advantage of this approach is that it should predict extracellular metabolite concentrations, which can be compared to the measurements of Fig 2 to validate the physiological realism of their simulations. How do the extracellular metabolite concentrations across each simulation in Fig 3. D compare to what the authors measured? Does this change if the Michaelis constant (discussed above) is changed to a more reasonable value?

The authors use Leaf68 in their simulations to show the different possible outcomes in co-culture. Do the same trends hold if the other organisms are modeled like this (e.g. Leaf203 or Leaf265 that are also auxotrophic for vitamins)? I suspect the exact same dynamics would occur since the models are likely extremely similar, yet we see the oscillations only in the Leaf68 community experimentally (Fig 3 A). Here, I think the simulations and modeling reported by the authors can be used to generate (alternative) hypotheses that experimentalists can test to understand/validate the mechanism being proposed here.

I appreciate the discussion on lines 273 – 288 about the cause of the oscillations, but more details and precision can be added here. What simulation conditions predict the onset of sustained vs dampened oscillations? From Fig 3 D it seems that higher vitamin leakage causes the oscillations, but experimentally the only vitamin secretion comes from Leaf257, which likely does not adjust its vitamin secretion based on the partner microbe (or does it?). Why do we not see the same oscillations for Leaf203 or Leaf265? Is there another mechanism generating the oscillations? Do any other parameter variations cause oscillations? Do the dampened oscillation communities "need less" vitamins? Are they slower growing? Are they more energy efficient (GAM or NGAM different)? What would be very enlightening is a "bifurcation" plot of the parameters vs. type of oscillation.

Additionally, my current understanding of the model is that growth rate bounds have been removed, but on lines 357 – 358 the authors posit that "species-specific growth rate" differences contribute to the modeled oscillations. How does this follow given their models and how was it tested computationally?

I am also interested to see the effect uptake kinetics have on the predictions. I suspect the currently used K_M might also be behind the observed oscillations, and that saturation in uptake kinetics may dampen all the predicted oscillations. This would also be interesting as that would suggest transporter effects may also contribute to the different predicted phenotypes. The strength of modeling is that all these hypotheses can be simply tested by tweaking the existing simulations — greatly aiding our understanding of the system.

Minor issues

- please add the estimated growth rates to Supplementary Figure 3
- the uptake flux in Supplementary Figure 12 needs units
